# Supplementary material for: Analyzing the interaction of human ACE2 and RBD of spike protein of SARS-CoV-2 in perspective of Omicron variant
Source: EXCLI J. 2022 Mar 10;21:610–20. doi: 10.17179/excli2022-4721 (PMC9149974; doi:10.17179/excli2022-4721)
Supplement: Supplementary information [file EXCLI-21-610-s-001.pdf]

EXCLI Journal 2022;21:610-620 – ISSN 1611-2156
Received: January 26, 2022, accepted: February 25, 2022, published: March 10, 2022

Supplementary material to:

Original article:

ANALYZING THE INTERACTION OF HUMAN ACE2 AND RBD OF SPIKE PROTEIN OF SARS-COV-2 IN PERSPECTIVE OF OMICRON VARIANT

Arijit Samanta[a], Syed Sahajada Mahafujul Alam[a], Safdar Ali[b], Mehboob Hoque[a]*

[a] Applied Bio-Chemistry Laboratory, Department of Biological Sciences, Aliah University, Kolkata 700160, India
[b] Clinical and Applied Genomics (CAG) Laboratory, Department of Biological Sciences, Aliah University, Kolkata 700160, India

* **Corresponding author:** Dr. Mehboob Hoque, Applied Bio-Chemistry Laboratory, Department of Biological Sciences, Aliah University, Kolkata 700160, India.
E-mails: m_hoque@aliah.ac.in, hoquemehboob@gmail.com

https://dx.doi.org/10.17179/excli2022-4721

This is an Open Access article distributed under the terms of the Creative Commons Attribution License (http://creativecommons.org/licenses/by/4.0/).

EXCLI Journal 2022;21:610-620 – ISSN 1611-2156
Received: January 26, 2022, accepted: February 25, 2022, published: March 10, 2022

Supplementary Table 1: Details of the SARS-CoV-2 variants. The first submitted complete genome sequences were retrieved from GISAID

Sl No	Variants	Virus name	Accession ID	Submission Date	First detected in	Genome size (nucle-otide)
**Reference**						
1	(**Wuhan Variant)**	WIV04	EPI_ISL_402124	11.01.2020	Wuhan	29891
**Variants of Concerns (VOCs)**						
2	B.1.1.7 GR/501Y.V1 (Alpha)	hCoV-19/England/CAMC-A58BA4/2020	EPI_ISL_596982	2020-10-27	United King-dom	29763
3	B.1.351 GH/501Y.V2 (Beta)	hCoV-19/South Africa/KRISP-K004599/2020	EPI_ISL_660629	2020-11-27	South Africa	29848
4	B.1.1.28.1, alias P.1 GR/501Y.V3 (Gamma)	hCoV-19/Brazil/AM-FIOCRUZ-20143138FN-R2/2020	EPI_ISL_811149	2021-01-13	Brazil	29593
5	B.1.617 G/452R.V3 (Delta)	hCoV-19/India/MH-NEERI-NGP-29787/2021	EPI_ISL_1360328	2021-03-25	India	29839
6	B.1.1.529 (Omicron)	hCoV19/Botswana/R40B60_BHP_ 3321001247/2021	EPI_ISL_6640917	2021-11-23	Botswana / Hong Kong / South Africa	29684
**Variants of Interest (VOIs)**						
7	C.37, GR/452Q.V1 (Lambda)	hCoV-19/Peru/LIM-INS-436/2021	EPI_ISL_1111334	2021-03-01	Peru	29901
8	B.1.621, GH (Mu)	hCoV-19/Colombia/RIS-VG-3857/2021	EPI_ISL_3369952	2021-08-12	Colombia	29902
**Variants Under Monitoring (VUMs)**						
9	C.1.2	hCoV-19/South Africa/KRISP-K020275/2021	EPI_ISL_3447714	2021-08-17	South Africa	29803
10	B.1.617.1 (Kappa)	hCoV-19/India/WB-1931300244219/2021	EPI_ISL_1357699	2021-03-25	India	29781
11	B.1.630	hCoV-19/Dominican Republic/33884/2021	EPI_ISL_3045385	2021-07-22	Dominican Republic	29799
12	B.1.640	hCoV-19/Congo/FCRM-100-A32.28.09.21/2021	EPI_ISL_5592661	2021-10-27	Republic of Congo	29683
**Formerly Under Monitoring**						
13	B.1.427 B.1.429 (Epsilon)	hCoV-19/USA/CA-CZB-12872/2020	EPI_ISL_648527	2020-11-21	United States of America	29816
14	R.1	hCoV-19/Japan/IC-0386/2020	EPI_ISL_736897	2020-12-26	Asia / Japan	29770
15	B.1.466.2	hCoV-19/Indonesia/JI-ITDua-33281NT/2020	EPI_ISL_877419	2021-01-27	Indonesia	29890
16	B.1.1.318	hCoV-19/England/ALDP-115DB1E/2021	EPI_ISL_937654	2021-02-05	Europe / United Kingdom / England	29753
17	B.1.1.519	hCoV-19/Japan/IC-0377/2020	EPI_ISL_721617	2020-12-21	Asia / Japan	29768
18	C.36.3	hCoV-19/USA/TX-HMH-MCoV-27355/2021	EPI_ISL_1237137	2021-03-13	North America / USA / Texas / Houston	29804
19	B.1.214.2	hCoV-19/England/ALDP-CB0759/2020	EPI_ISL_760951	2021-01-04	Europe / United Kingdom / England	29750

EXCLI Journal 2022;21:610-620 – ISSN 1611-2156
Received: January 26, 2022, accepted: February 25, 2022, published: March 10, 2022

Supplementary Table 1 (cont.): Details of the SARS-CoV-2 variants. The first submitted complete genome sequences were retrieved from GISAID

Sl No	Variants	Virus name	Accession ID	Submission Date	First detected in	Genome size (nucle-otide)
20	B.1.1.523	hCoV-19/Switzerland/GE-33615516/2021	EPI_ISL_1448584	2021-04-02	Europe / Swit-zerland / Ge-neva	29856
21	B.1.619	hCoV-19/Germany/BE-RKI-I-020766/2021	EPI_ISL_1150929	2021-03-04	Europe / Ger-many / Berlin	29809
22	B.1.620	hCoV-19/Lithua-nia/LTU000_NMVRI55624/2021	EPI_ISL_1579527	2021-04-13	Europe / Lithu-ania / Vilniaus apskritis	29840
23	B.1.526 (Lota)	hCoV-19/USA/NY-Wadsworth-290357-01/2020	EPI_ISL_765494	2021-01-04	United States of America	29782
24	B.1.525 (Eta)	hCoV-19/England/CAMC-C769B3/2020	EPI_ISL_760883	2021-01-04	United Kingdom	29709
25	AV.1	hCoV-19/England/QEUH-148F257/2021	EPI_ISL_1595332	2021-04-15	United Kingdom	29764
26	AT.1	hCoV-19/Russia/PSK-16/2021	EPI_ISL_1259283	2021-03-16	Russian Federation	29803

EXCLI Journal 2022;21:610-620 – ISSN 1611-2156
Received: January 26, 2022, accepted: February 25, 2022, published: March 10, 2022

Supplementary Table 2: SWISS Model parameters of the predicted 3D structures of RBDs of SARS-CoV-2 variants

Variants	Accession ID	Template Swiss model ID	Features of template used in generating S Protein model					Quality assessment of the S Protein model	
			Sequence Identity	Method	Resolution	Sequence similarity	Coverage	QMEAN	GMQE
B.1.1.7 GR/501Y.V1 (Alpha)	EPI_ISL_596982	7lwv.1.A	100.00%	EM	-	0.63	1.00	0.78	0.84
B.1.351 GH/501Y.V2 (Beta)	EPI_ISL_660629	7lww.1.A	100.00%	EM	-	0.63	1.00	0.77	0.84
B.1.1.28.1, alias P.1 GR/501Y.V3 (Gamma)	EPI_ISL_811149	7v78.1.A	100.00%	EM	-	0.63	1.00	0.74	0.81
B.1.617 G/452R.V3 (Delta)	EPI_ISL_1360328	7cn4.1.A	89.69%	EM	-	0.59	1.00	0.70	0.77
B.1.1.529 (Omicron)	EPI_ISL_6640917	7lyq.1.A	94.17%	EM	-	0.61	1.00	0.74	0.81
C.37, GR/452Q.V1 (Lambda)	EPI_ISL_1111334	6zgf.1.A	89.69%	EM	-	0.59	1.00	0.72	0.78
B.1.621, GH (Mu)	EPI_ISL_3369952	6xc4.1.A	99.55%	X-ray	2.34 Å	0.63	1.00	0.80	0.80
B.1.427 B.1.429 (Epsilon)	EPI_ISL_648527	7cn4.1.A	89.69%	EM	-	0.59	1.00	0.70	0.77
R.1	EPI_ISL_736897	6xc4.1.A	99.55%	X-ray	2.34 Å	0.63	1.00	0.80	0.80
B.1.466.2	EPI_ISL_877419	6xc4.1.A	99.55%	X-ray	2.34 Å	0.63	1.00	0.80	0.80
B.1.1.318	EPI_ISL_937654	6xc4.1.A	99.55%	X-ray	2.34 Å	0.63	1.00	0.80	0.80
B.1.1.519	EPI_ISL_721617	6xc4.1.A	99.55%	X-ray	2.34 Å	0.63	1.00	0.80	0.80
C.36.3	EPI_ISL_1237137	7lwv.1.A	100.00%	EM	-	0.63	1.00	0.78	0.84
B.1.214.2	EPI_ISL_760951	6xc4.1.A	99.55%	X-ray	2.34 Å	0.63	1.00	0.80	0.80
B.1.1.523	EPI_ISL_1448584	6xc4.1.A	99.10%	X-ray	2.34 Å	0.63	1.00	0.80	0.80
B.1.619	EPI_ISL_1150929	6xc4.1.A	99.55%	X-ray	2.34 Å	0.63	1.00	0.80	0.80
B.1.620	EPI_ISL_1579527	6xc4.1.A	99.10%	X-ray	2.34 Å	0.63	1.00	0.80	0.80
C.1.2	EPI_ISL_3447714	7lwv.1.A	99.10%	EM	-	0.63	1.00	0.77	0.84
B.1.617.1 (Kappa)	EPI_ISL_1357699	7cn4.1.A	89.69%	EM	-	0.59	1.00	0.70	0.77
B.1.526 (Lota)	EPI_ISL_765494	6xc4.1.A	99.55%	X-ray	2.34 Å	0.63	1.00	0.80	0.80
B.1.525 (Eta)	EPI_ISL_760883	6xc4.1.A	99.55%	X-ray	2.34 Å	0.63	1.00	0.80	0.80
B.1.630	EPI_ISL_3045385	7sbl.1.A	99.10%	EM	3.44 Å	0.63	1.00	0.73	0.10
B.1.640	EPI_ISL_5592661	7lwv.1.A	98.21%	EM	-	0.63	1.00	0.78	0.84
AV.1	EPI_ISL_1595332	6xc4.1.A	99.10%	X-ray	2.34 Å	0.63	1.00	0.81	0.80
AT.1	EPI_ISL_1259283	6xc4.1.A	99.55%	X-ray	2.34 Å	0.63	1.00	0.80	0.80

EXCLI Journal 2022;21:610-620 – ISSN 1611-2156
Received: January 26, 2022, accepted: February 25, 2022, published: March 10, 2022

Supplementary Table 3: S protein RBD model validation parameters obtained in SAVES and ProSA

Variants	Accession ID	ERRAT	% of the residues have averaged 3D-1D score >= 0.2	Ramachandran plot			G-factors	*Z*-Score
				core	allow	gener		
B.1.1.7 GR/501Y.V1 (Alpha)	EPI_ISL_596982	**94.5364**	93.12%	88.4%	11.2%	0.3%	-0.19	**-5.93**
B.1.351 GH/501Y.V2 (Beta)	EPI_ISL_660629	**94.0594**	91.18%	89.3%	10.5%	0.2%	-0.20	**-5.62**
B.1.1.28.1, alias P.1 GR/501Y.V3 (Gamma)	EPI_ISL_811149	**81.1287**	87.89%	87.0%	12.4%	0.3%	-0.14	**-5.48**
B.1.617 G/452R.V3 (Delta)	EPI_ISL_1360328	**93.6594**	93.72%	78.6%	21.2%	0.2%	-0.21	**-5.58**
B.1.1.529 (Omicron)	EPI_ISL_6640917	**96.6443**	95.37%	87.7%	11.8%	0.5%	-0.24	**-5.56**
C.37, GR/452Q.V1 (Lambda)	EPI_ISL_1111334	**80**	96.71%	83.2%	16.2%	0.5%	-0.22	**-5.52**
B.1.621, GH (Mu)	EPI_ISL_3369952	**97.076**	100.00%	90.6%	9.4%	0.0	-0.14	**-5.96**
B.1.427 B.1.429 (Epsilon)	EPI_ISL_648527	**93.8739**	93.57%	78.4%	20.9%	0.7%	-0.22	**-5.6**
R.1	EPI_ISL_736897	**96.5116**	97.99%	90.6%	9.4%	0.0%	-0.13	**-5.88**
B.1.466.2	EPI_ISL_877419	**96.5116**	96.98%	90.6%	9.4%	0.0%	-0.14	**-5.87**
B.1.1.318	EPI_ISL_937654	**96.5116**	97.99%	90.6%	9.4%	0.0%	-0.13	**-5.88**
B.1.1.519	EPI_ISL_721617	**97.0588**	97.99%	90.6%	9.4%	0.0%	-0.15	**-6.01**
C.36.3	EPI_ISL_1237137	**94.5364**	93.12%	88.4%	11.2%	0.3%	-0.19	**-5.93**
B.1.214.2	EPI_ISL_760951	**96.5116**	97.99%	90.6%	9.4%	0.0%	-0.14	**-5.99**
B.1.1.523	EPI_ISL_1448584	**96.5116**	98.49%	90.6%	9.4%	0.0%	-0.14	**-5.84**
B.1.619	EPI_ISL_1150929	**96.5116**	97.99%	90.6%	9.4%	0.0%	-0.13	**-5.88**
B.1.620	EPI_ISL_1579527	**97.076**	97.99%	90.6%	9.4%	0.0%	-0.13	**-5.92**
C.1.2	EPI_ISL_3447714	**94.4816**	93.12%	88.4%	11.2%	0.3%	-0.18	**-6**
B.1.617.1 (Kappa)	EPI_ISL_1357699	**93.6594**	93.72%	78.6%	21.2%	0.2%	-0.21	**-5.58**
B.1.526 (Lota)	EPI_ISL_765494	**96.5116**	97.99%	90.6%	9.4%	0.0%	-0.13	**-5.88**
B.1.525 (Eta)	EPI_ISL_760883	**96.5116**	97.99%	90.6%	9.4%	0.0%	-0.13	**-5.88**
B.1.630	EPI_ISL_3045385	**89.2857**	93.27%	86.5%	13.1%	0.3%	-0.25	**-5.28**
B.1.640	EPI_ISL_5592661	**94.01**	92.97%	88.3%	11.4%	0.3%	-0.19	**-6.08**
AV.1	EPI_ISL_1595332	**96.5116**	96.98%	90.6%	9.4%	0.0%	-0.13	**-5.87**
AT.1	EPI_ISL_1259283	**96.5116**	97.99%	90.6%	9.4%	0.0%	-0.13	**-5.88**

Supplementary Table 4: Comparative analysis of interacting amino acid residues between hACE and Omicron RBD with all other variant RBD

Variants	S Protein RBD			hACE2 Receptor		
	**No. of Total Interacting Amino Acid(s)**	**No. of Amino Acid residues identical to Omicron**	**% of identical residues with Omicron**	**No. of Total Interacting Amino Acid(s)**	**No. of Amino Acid residues identical to Omicron**	**% of identical residues with Omicron**
EPI ISL 402124(WIV04)	17	0	**0**	20	3	15
EPI ISL 596982(B.1.1.7)	29	26	89.66	47	22	46.81
EPI ISL 660629(B.1.351)	32	28	**90.32**	56	33	58.93
EPI ISL 811149(B.1.1.28.1)	25	13	52	34	1	2.94
EPI ISL 1360328(B.1.617)	33	27	81.82	58	25	43.10
EPI ISL 1111334(C.37)	21	21	**100**	46	14	30.43
EPI ISL 3369952(B.1.621)	33	0	**0**	32	5	15.63
EPI ISL 648527(B.1.427)	33	27	81.82	58	24	41.38
EPI ISL 736897(R.1)	23	0	**0**	34	2	5.88
EPI ISL 877419(B.1.466.2)	36	0	**0**	32	5	15.63
EPI ISL 937654(B.1.1.318)	32	0	**0**	34	2	5.88
EPI ISL 721617(B.1.1.519)	32	0	**0**	33	5	15.15
EPI ISL 1237137(C.36.3)	31	26	83.87	47	22	46.81
EPI ISL 760951(B.1.214.2)	33	0	**0**	30	5	16.67
EPI ISL 1448584 (B.1.1.523)	31	0	**0**	35	2	5.71
EPI ISL 1150929(B.1.619)	34	0	**0**	34	2	5.88
EPI ISL 1579527(B.1.620)	23	0	**0**	27	0	**0**
EPI ISL 3447714(C.1.2)	28	26	**92.86**	45	23	51.11
EPI ISL 1357699(B.1.617.1)	33	27	81.82	58	25	43.10
EPI ISL 765494(B.1.526)	32	0	**0**	34	2	5.88
EPI ISL 760883(B.1.525)	32	0	**0**	34	2	5.88
EPI ISL 3045385(B.1.630)	23	19	82.61	49	11	22.45
EPI ISL 5592661(B.1.640)	29	26	89.66	47	22	46.81
EPI ISL 1595332(AV.1)	34	0	**0**	33	2	6.06
EPI ISL 1259283(AT.1)	32	0	**0**	34	2	5.89

EXCLI Journal 2022;21:610-620 – ISSN 1611-2156
Received: January 26, 2022, accepted: February 25, 2022, published: March 10, 2022

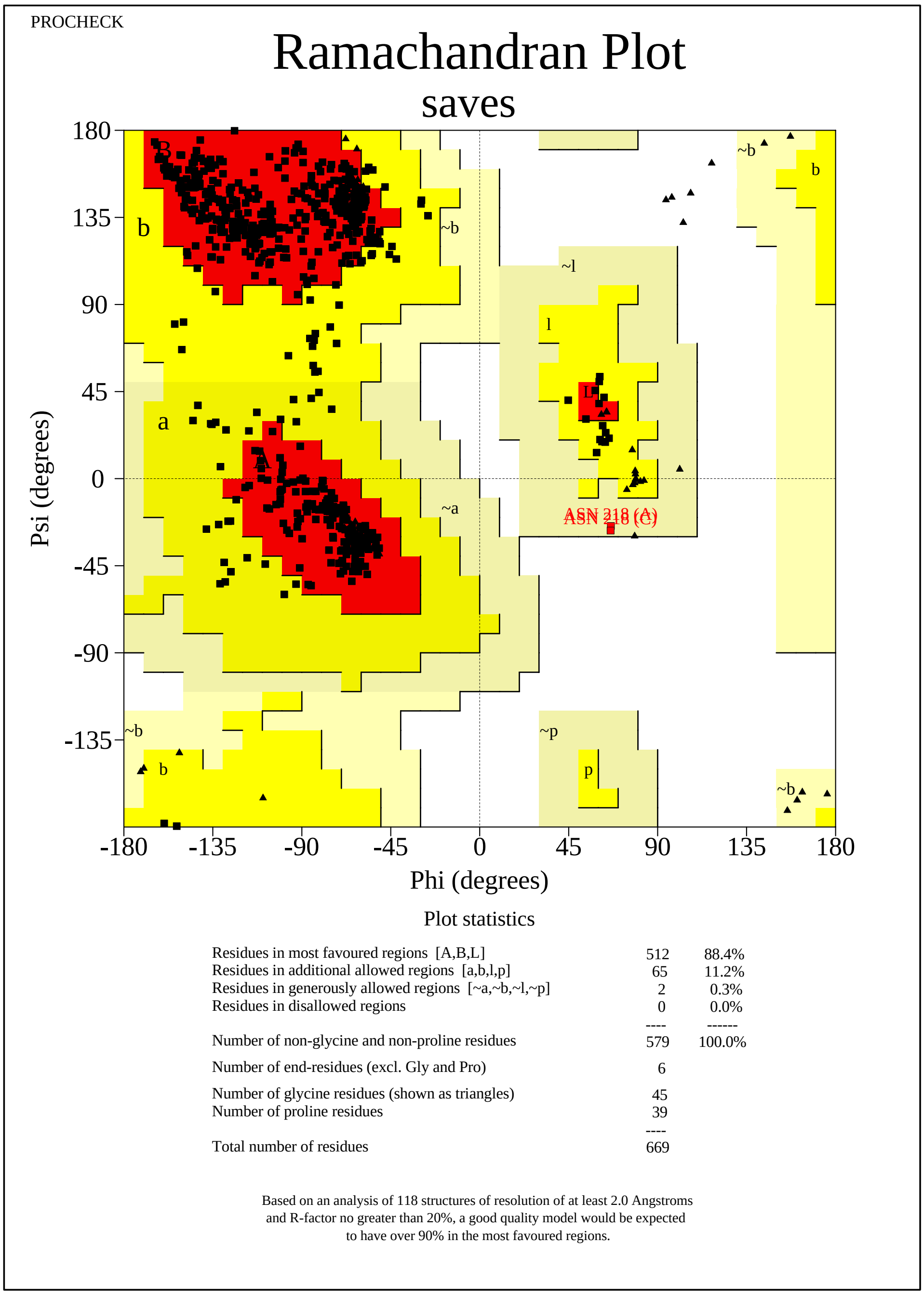

saves_01.ps

Supplementary Figure1(A): The PROCHECK Ramachandran plot for the validation of the 3D model of Spike RBD of SARS-CoV-2 B.1.1.7(Alpha) variant (EPI_ISL_596982)

Received: January 26, 2022, accepted: February 25, 2022, published: March 10, 2022

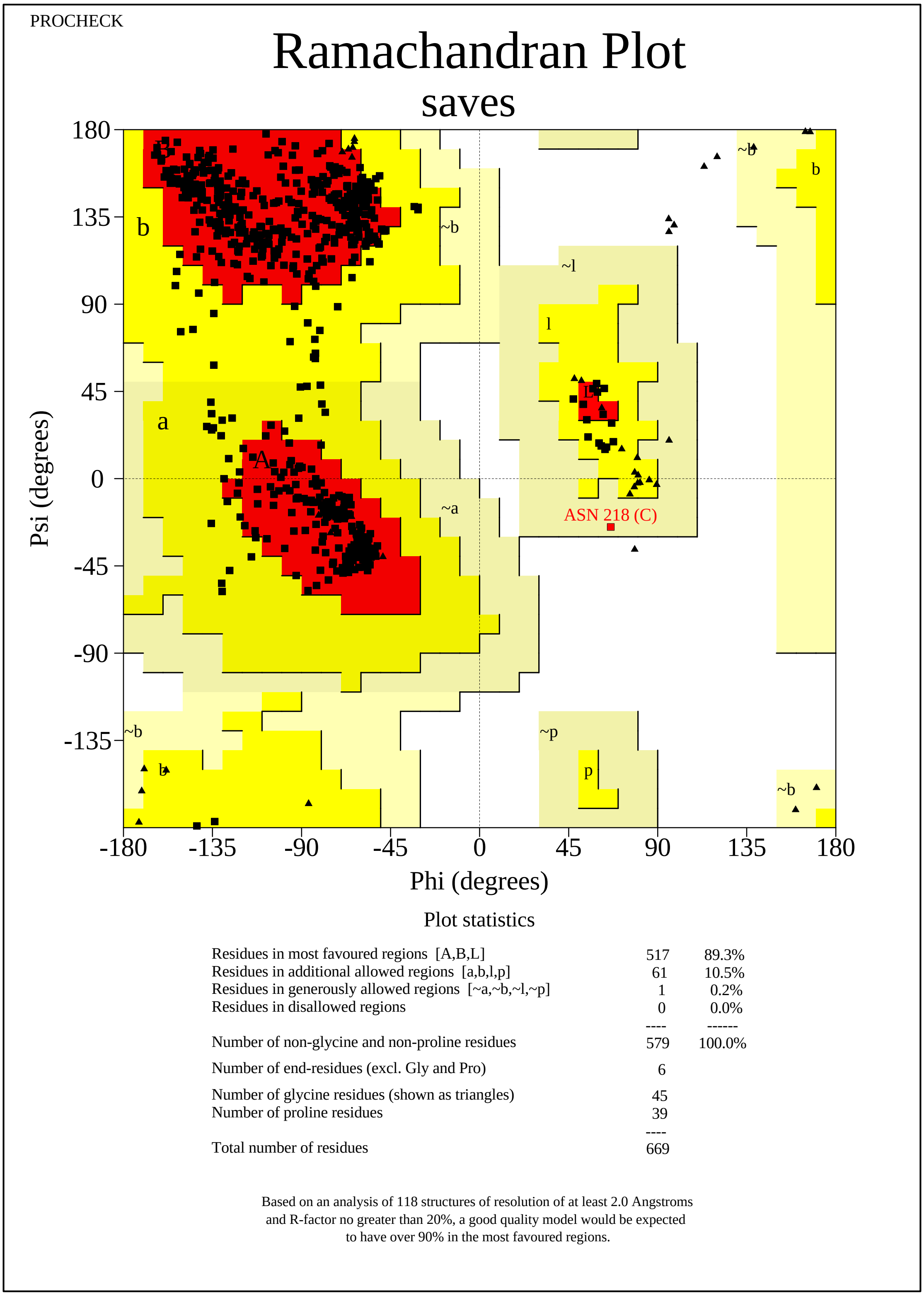

saves_01.ps

Supplementary Figure 1(B): The PROCHECK Ramachandran plot for the validation of the 3D model of Spike RBD of SARS-CoV-2 B.1.351(Beta) variant (EPI_ISL_660629)

EXCLI Journal 2022;21:610-620 – ISSN 1611-2156
Received: January 26, 2022, accepted: February 25, 2022, published: March 10, 2022

PROCHECK

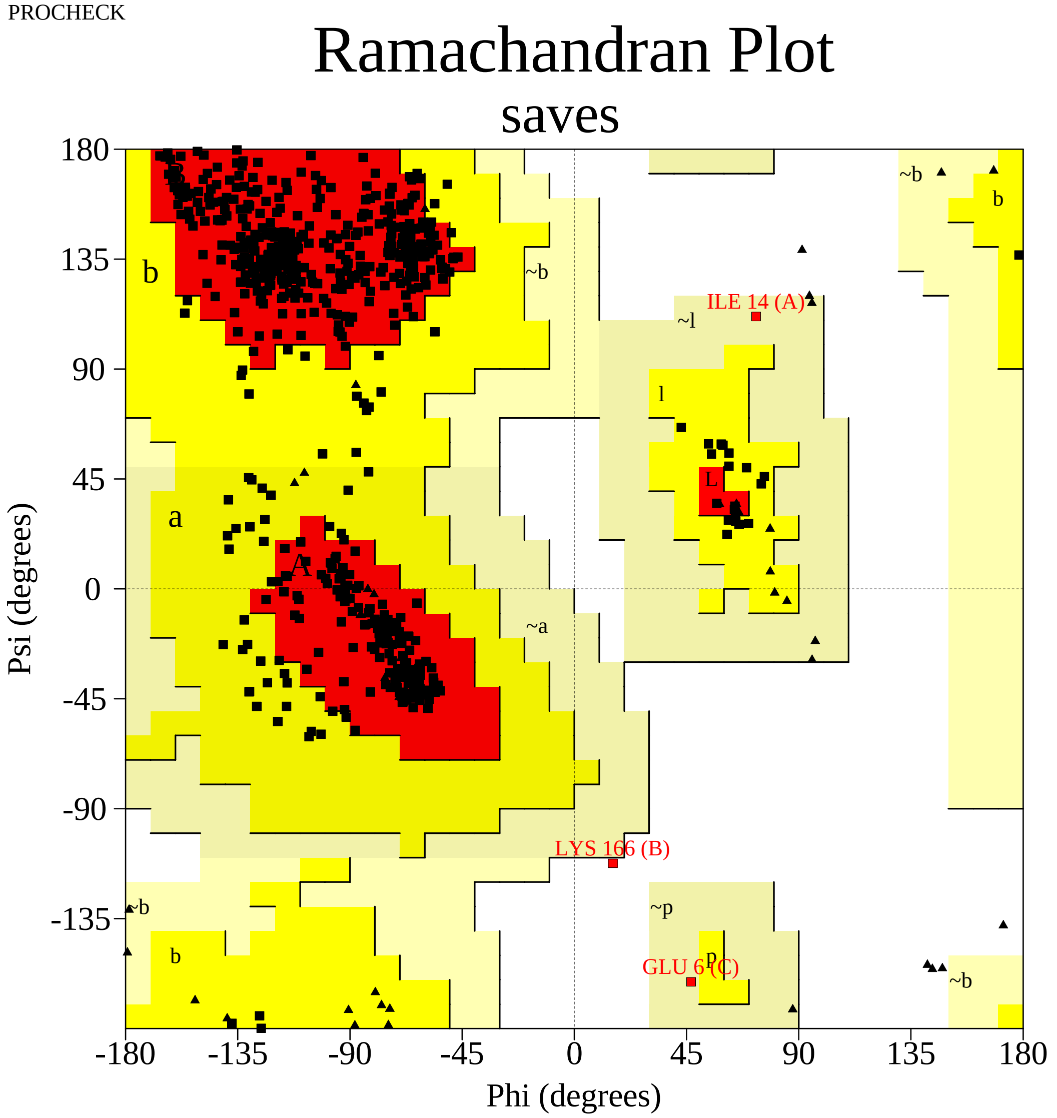

Plot statistics

Residues in most favoured regions [A,B,L]	504	87.0%
Residues in additional allowed regions [a,b,l,p]	72	12.4%
Residues in generously allowed regions [~a,~b,~l,~p]	2	0.3%
Residues in disallowed regions	1	0.2%
	----	------
Number of non-glycine and non-proline residues	579	100.0%
Number of end-residues (excl. Gly and Pro)	6	
Number of glycine residues (shown as triangles)	45	
Number of proline residues	39	

Total number of residues	669	

Based on an analysis of 118 structures of resolution of at least 2.0 Angstroms and R-factor no greater than 20%, a good quality model would be expected to have over 90% in the most favoured regions.

saves_01.ps

Supplementary Figure 1(C): The PROCHECK Ramachandran plot for the validation of the 3D model of Spike RBD of SARS-CoV-2 B.1.1.28.1(Gamma) variant EPI_ISL_811149

Received: January 26, 2022, accepted: February 25, 2022, published: March 10, 2022

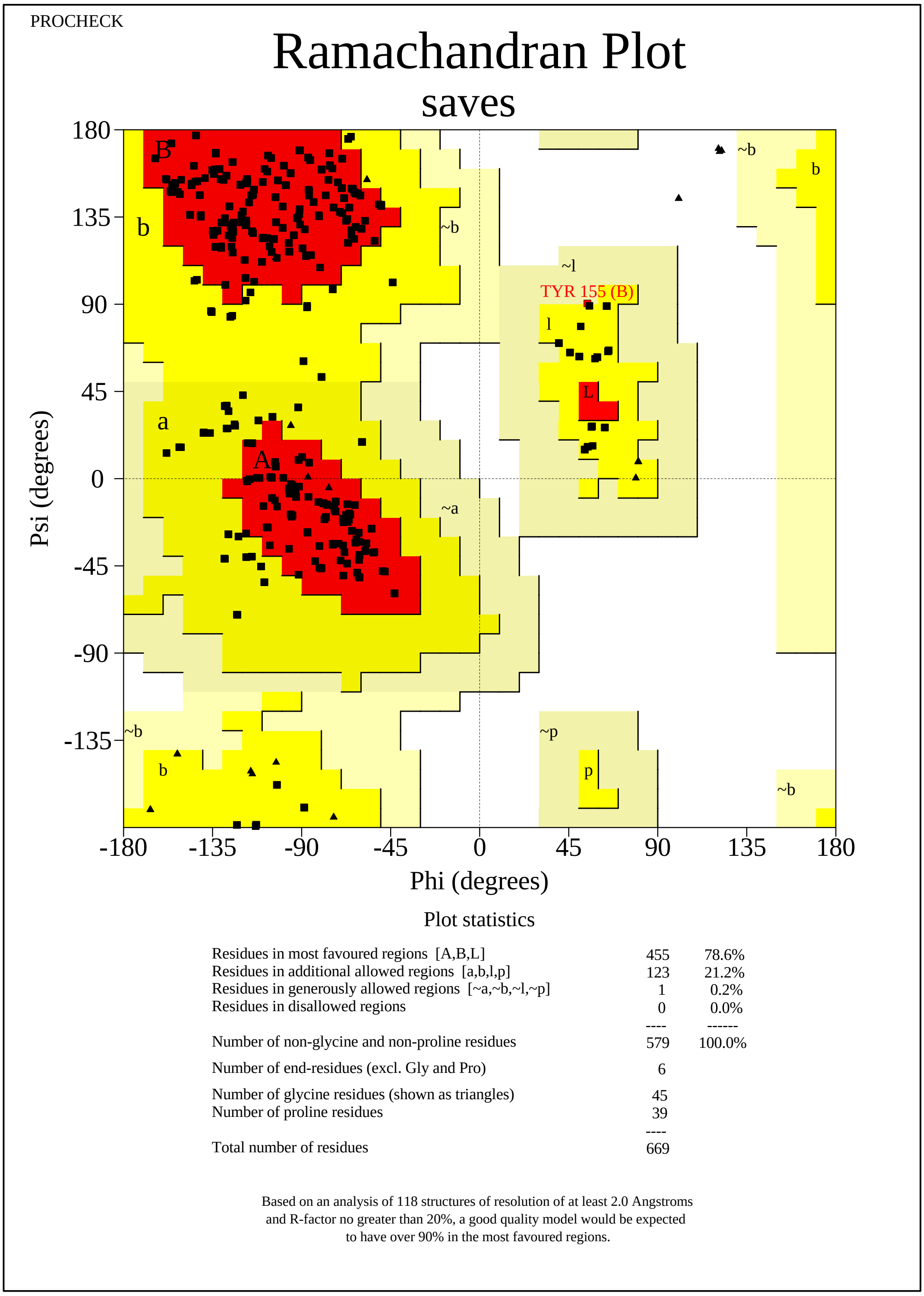

saves_01.ps

Supplementary Figure 1(D): The PROCHECK Ramachandran plot for the validation of the 3D model of Spike RBD of SARS-CoV-2 B.1.617(Delta) variant (EPI_ISL_1360328)

EXCLI Journal 2022;21:610-620 – ISSN 1611-2156
Received: January 26, 2022, accepted: February 25, 2022, published: March 10, 2022

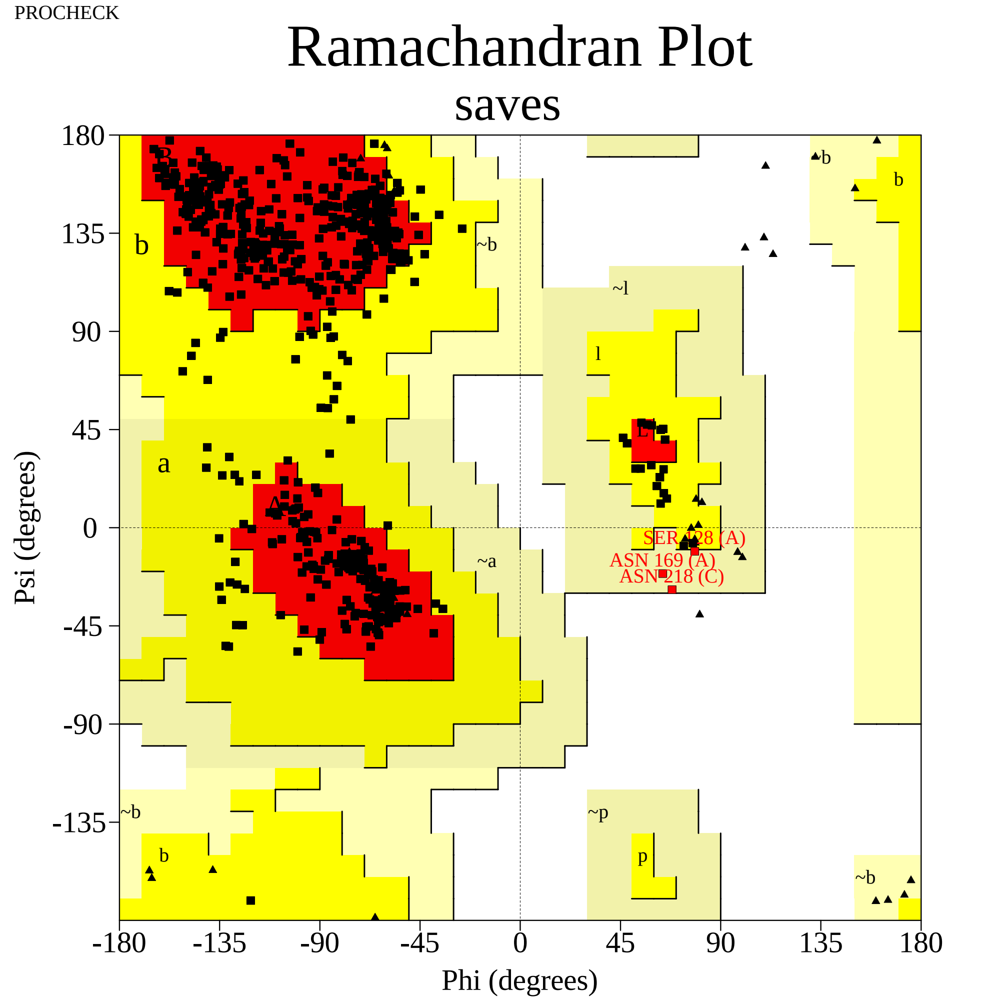

Plot statistics

Residues in most favoured regions [A,B,L]	513	87.7%
Residues in additional allowed regions [a,b,l,p]	69	11.8%
Residues in generously allowed regions [~a,~b,~l,~p]	3	0.5%
Residues in disallowed regions	0	0.0%
	----	------
Number of non-glycine and non-proline residues	585	100.0%
Number of end-residues (excl. Gly and Pro)	6	
Number of glycine residues (shown as triangles)	36	
Number of proline residues	42	

Total number of residues	669	

Based on an analysis of 118 structures of resolution of at least 2.0 Angstroms and R-factor no greater than 20%, a good quality model would be expected to have over 90% in the most favoured regions.

saves_01.ps

Supplementary Figure 1(E): The PROCHECK Ramachandran plot for the validation of the 3D model of Spike RBD of SARS-CoV-2 B.1.1.529(Omicron) variant (EPI_ISL_6640917)

EXCLI Journal 2022;21:610-620 – ISSN 1611-2156
Received: January 26, 2022, accepted: February 25, 2022, published: March 10, 2022

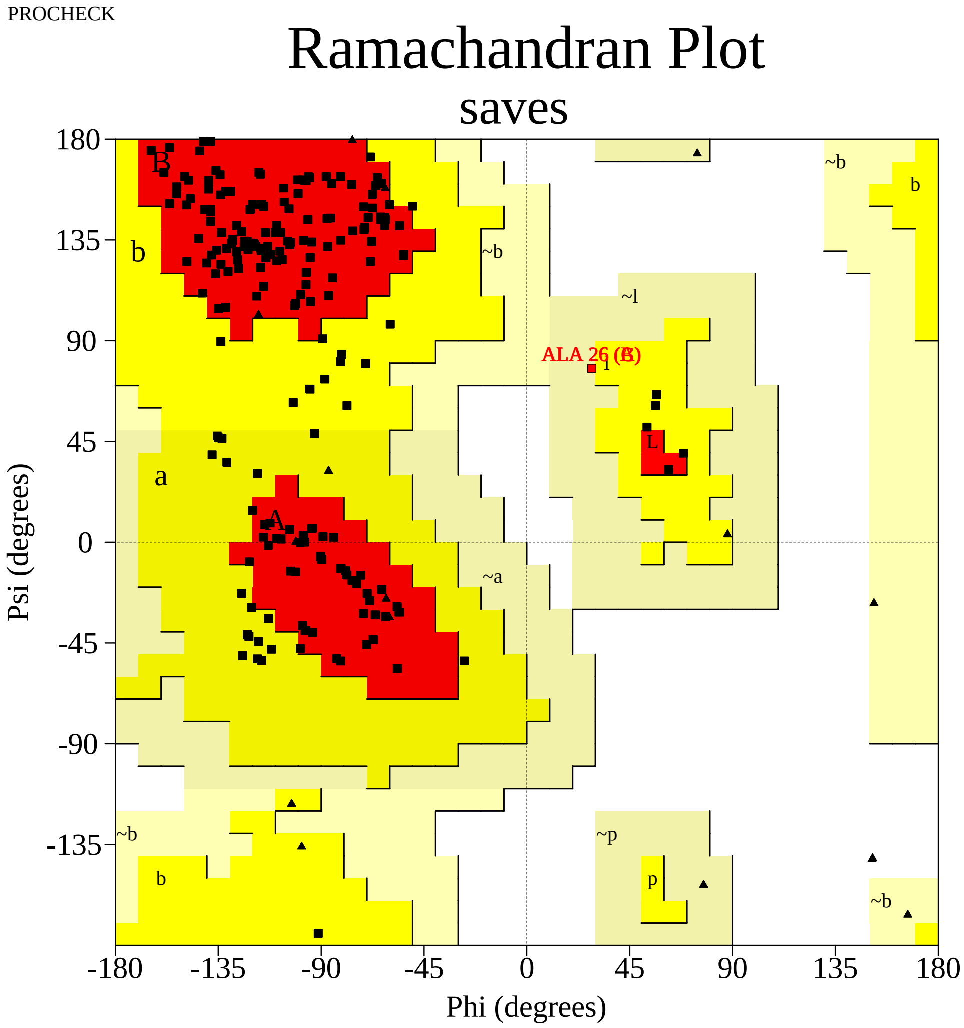

Plot statistics

Residues in most favoured regions [A,B,L]	482	83.2%
Residues in additional allowed regions [a,b,l,p]	94	16.2%
Residues in generously allowed regions [~a,~b,~l,~p]	3	0.5%
Residues in disallowed regions	0	0.0%
	----	------
Number of non-glycine and non-proline residues	579	100.0%
Number of end-residues (excl. Gly and Pro)	6	
Number of glycine residues (shown as triangles)	45	
Number of proline residues	39	

Total number of residues	669	

Based on an analysis of 118 structures of resolution of at least 2.0 Angstroms and R-factor no greater than 20%, a good quality model would be expected to have over 90% in the most favoured regions.

saves_01.ps

Supplementary Figure 1(F): The PROCHECK Ramachandran plot for the validation of the 3D model of Spike RBD of SARS-CoV-2 C.37(Lambda) variant (EPI_ISL_1111334)

EXCLI Journal 2022;21:610-620 – ISSN 1611-2156
Received: January 26, 2022, accepted: February 25, 2022, published: March 10, 2022

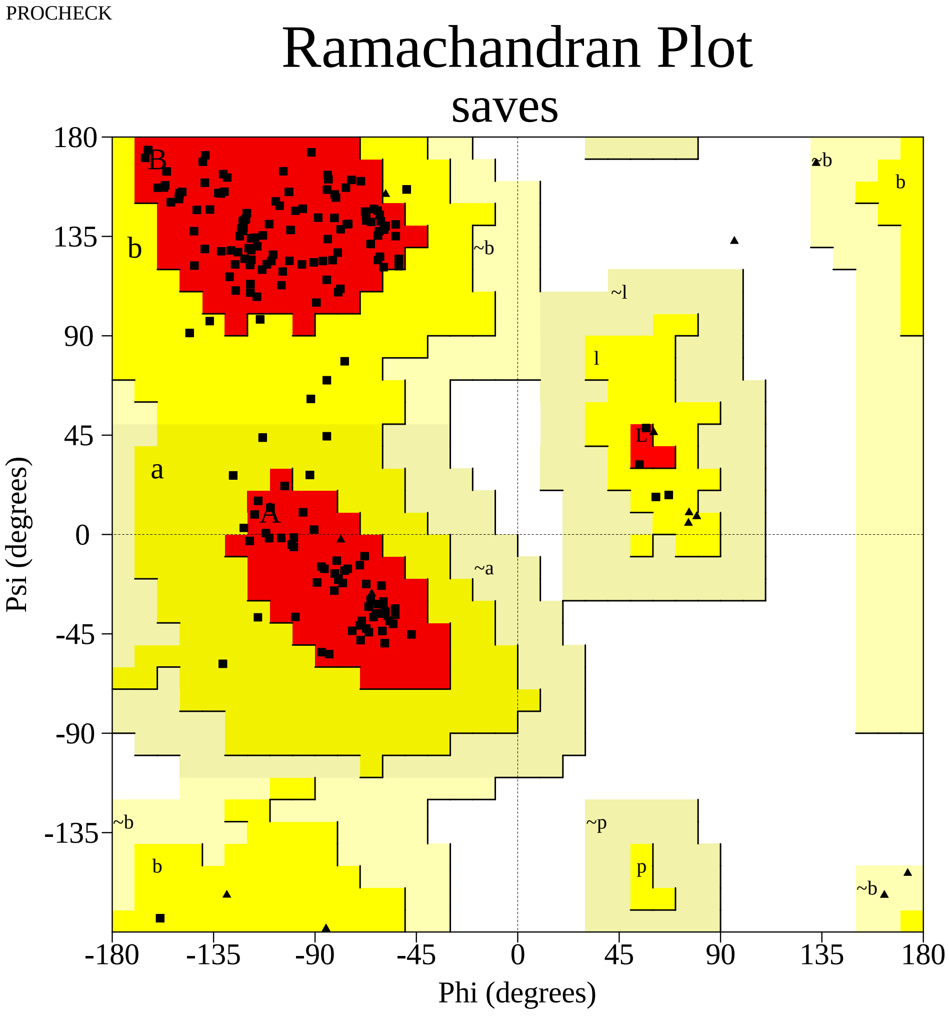

Plot statistics

Residues in most favoured regions [A,B,L]	155	90.6%
Residues in additional allowed regions [a,b,l,p]	16	9.4%
Residues in generously allowed regions [~a,~b,~l,~p]	0	0.0%
Residues in disallowed regions	0	0.0%
	----	------
Number of non-glycine and non-proline residues	171	100.0%
Number of end-residues (excl. Gly and Pro)	2	
Number of glycine residues (shown as triangles)	15	
Number of proline residues	11	

Total number of residues	199	

Based on an analysis of 118 structures of resolution of at least 2.0 Angstroms
and R-factor no greater than 20%, a good quality model would be expected
to have over 90% in the most favoured regions.

saves_01.ps

Supplementary Figure 1(G): The PROCHECK Ramachandran plot for the validation of the 3D model of Spike RBD of SARS-CoV-2 B.1.621(Mu) variant (EPI_ISL_3369952)

EXCLI Journal 2022;21:610-620 – ISSN 1611-2156
Received: January 26, 2022, accepted: February 25, 2022, published: March 10, 2022

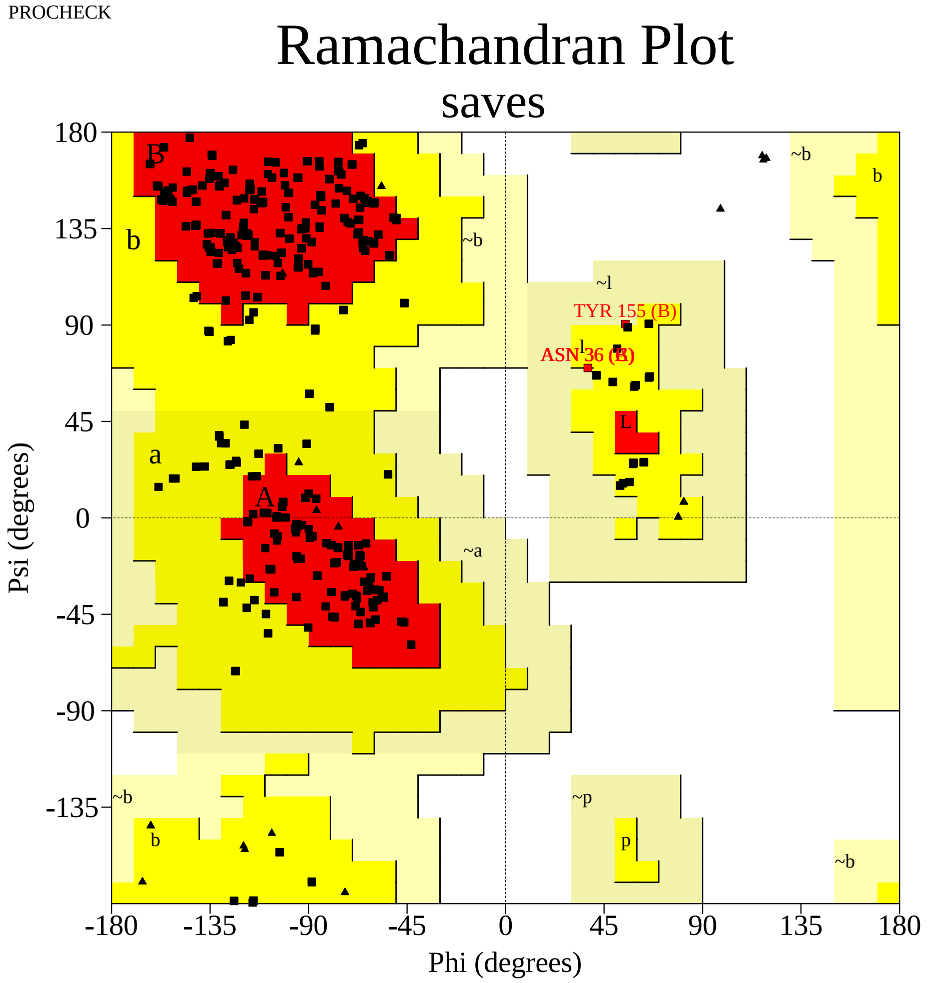

Plot statistics

Residues in most favoured regions [A,B,L]	454	78.4%
Residues in additional allowed regions [a,b,l,p]	121	20.9%
Residues in generously allowed regions [~a,~b,~l,~p]	4	0.7%
Residues in disallowed regions	0	0.0%
	----	------
Number of non-glycine and non-proline residues	579	100.0%
Number of end-residues (excl. Gly and Pro)	6	
Number of glycine residues (shown as triangles)	45	
Number of proline residues	39	

Total number of residues	669	

Based on an analysis of 118 structures of resolution of at least 2.0 Angstroms and R-factor no greater than 20%, a good quality model would be expected to have over 90% in the most favoured regions.

saves_01.ps

Supplementary Figure 1(H): The PROCHECK Ramachandran plot for the validation of the 3D model of Spike RBD of SARS-CoV-2 B.1.427/B.1.429(Epsilon) variant (EPI_ISL_648527)

EXCLI Journal 2022;21:610-620 – ISSN 1611-2156
Received: January 26, 2022, accepted: February 25, 2022, published: March 10, 2022

PROCHECK

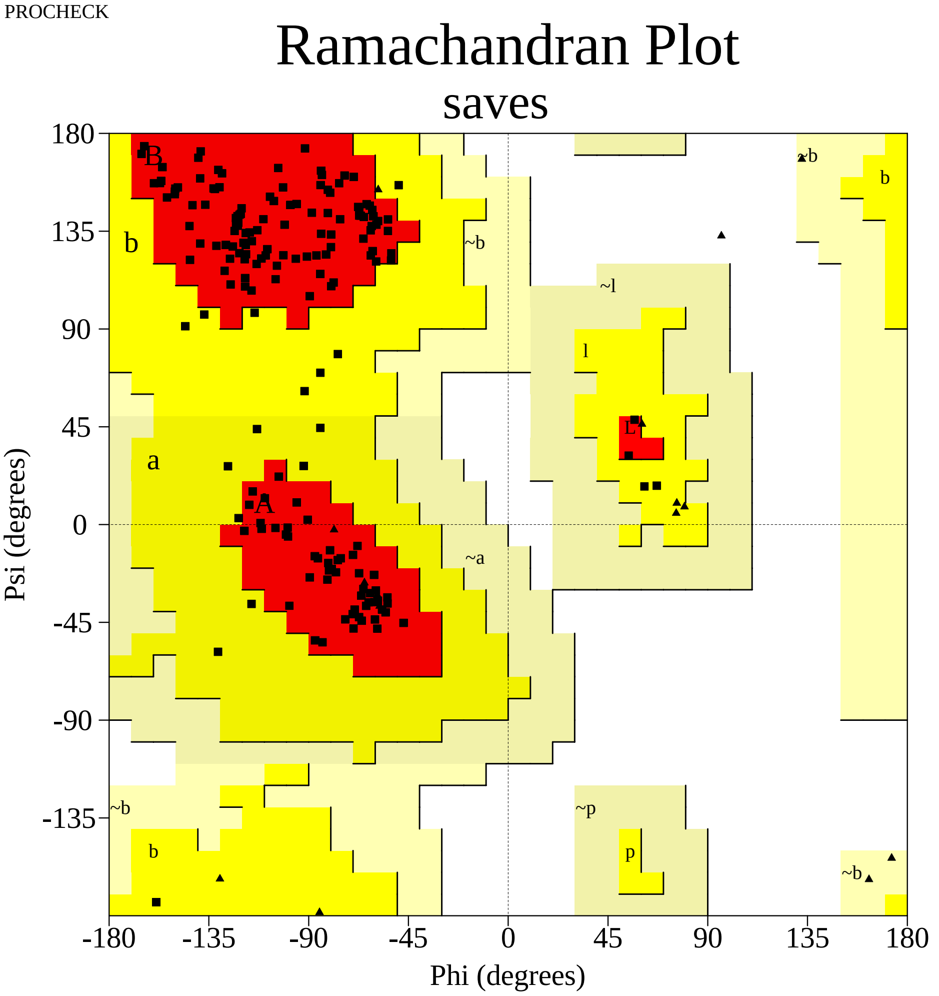

Plot statistics

Residues in most favoured regions [A,B,L]	155	90.6%
Residues in additional allowed regions [a,b,l,p]	16	9.4%
Residues in generously allowed regions [~a,~b,~l,~p]	0	0.0%
Residues in disallowed regions	0	0.0%
	----	------
Number of non-glycine and non-proline residues	171	100.0%
Number of end-residues (excl. Gly and Pro)	2	
Number of glycine residues (shown as triangles)	15	
Number of proline residues	11	

Total number of residues	199	

Based on an analysis of 118 structures of resolution of at least 2.0 Angstroms
and R-factor no greater than 20%, a good quality model would be expected
to have over 90% in the most favoured regions.

saves_01.ps

Supplementary Figure 1(I): The PROCHECK Ramachandran plot for the validation of the 3D model of Spike RBD of SARS-CoV-2 B.1.1.519 variant (EPI_ISL_721617)

EXCLI Journal 2022;21:610-620 – ISSN 1611-2156
Received: January 26, 2022, accepted: February 25, 2022, published: March 10, 2022

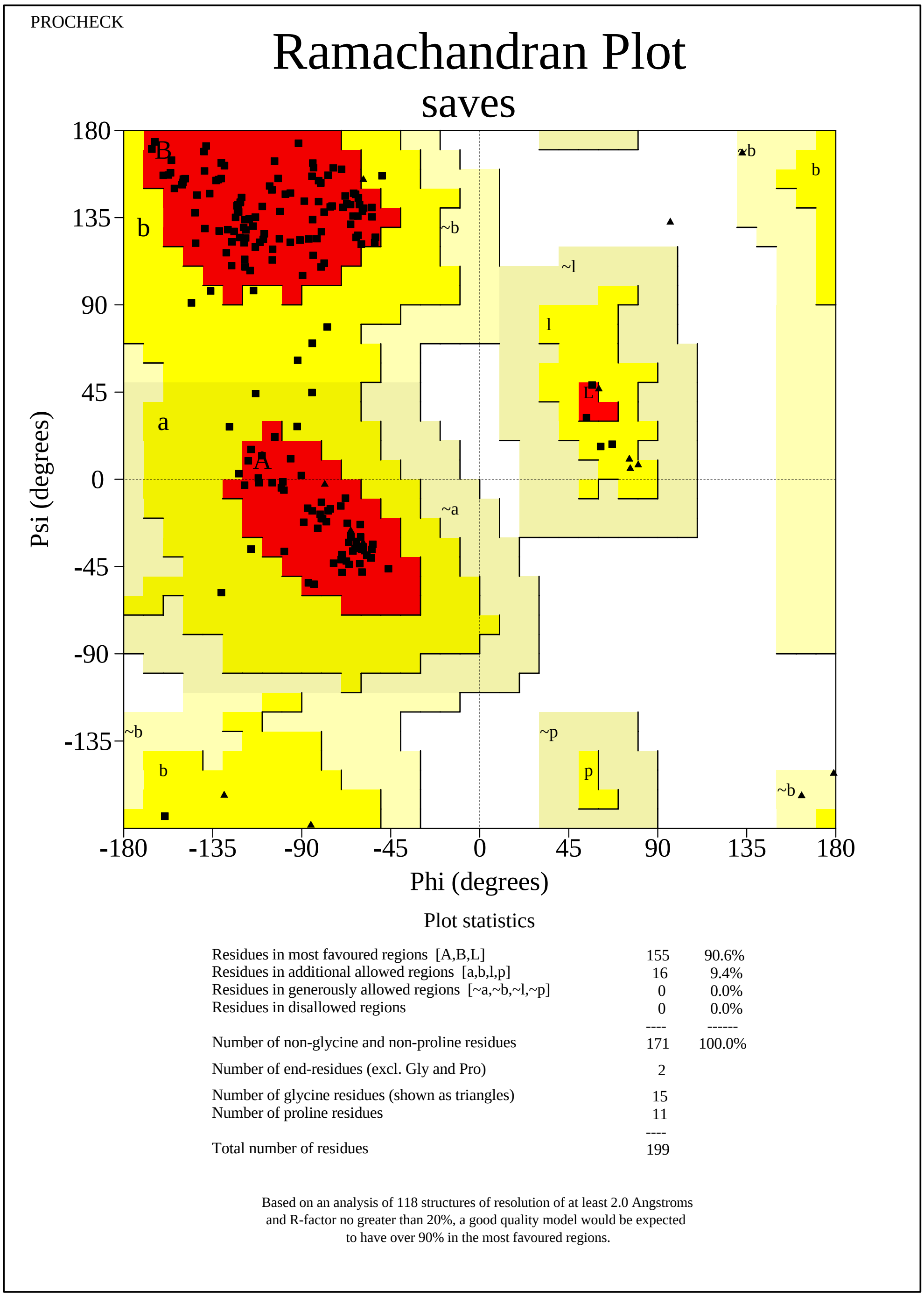

saves_01.ps

Supplementary Figure 1(J): The PROCHECK Ramachandran plot for the validation of the 3D model of Spike RBD of SARS-CoV-2 R.1 variant (EPI_ISL_736897)

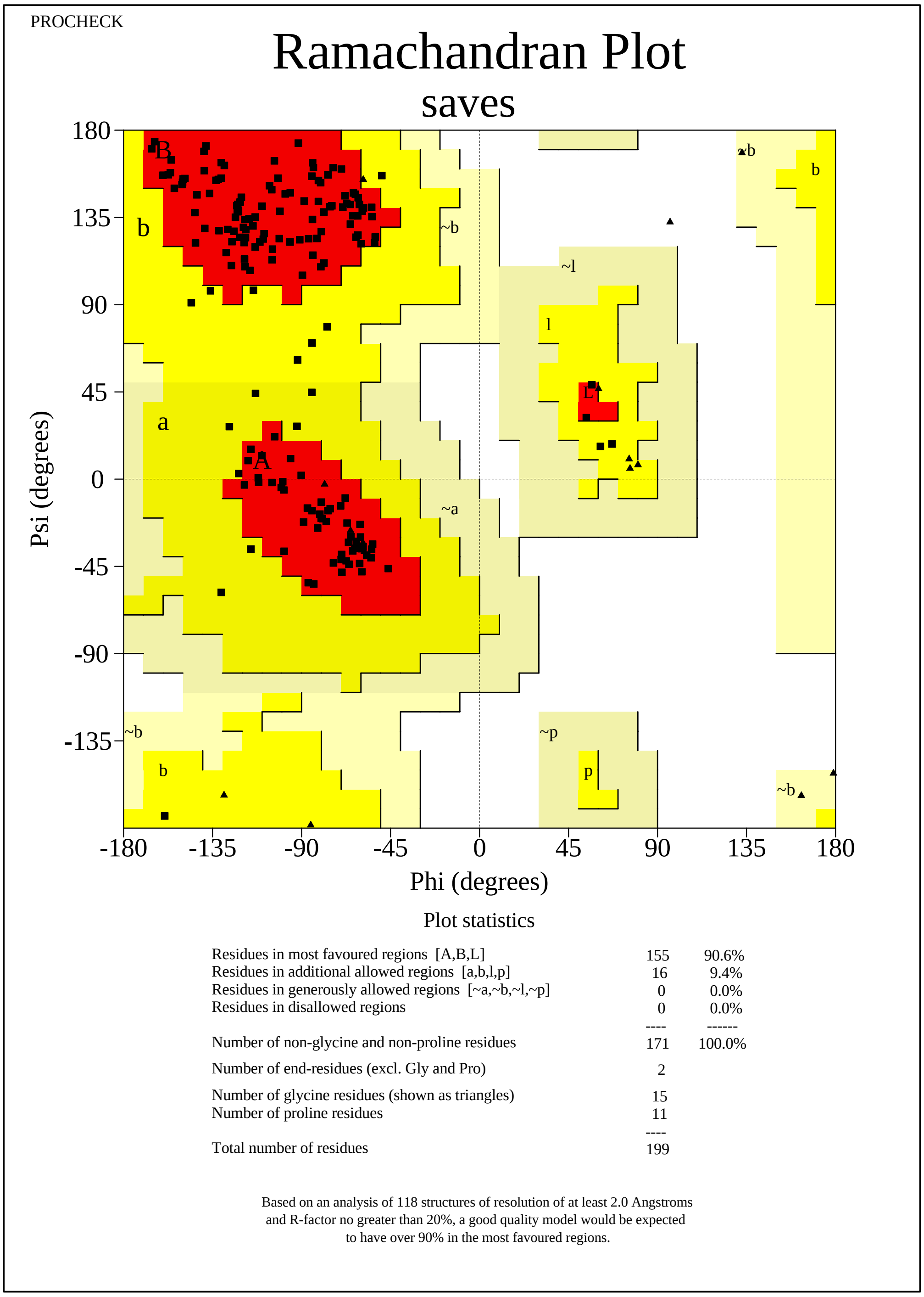

saves_01.ps

Supplementary Figure 1(K): The PROCHECK Ramachandran plot for the validation of the 3D model of Spike RBD of SARS-CoV-2 B.1.525(Eta) variant (EPI_ISL_760883)

EXCLI Journal 2022;21:610-620 – ISSN 1611-2156
Received: January 26, 2022, accepted: February 25, 2022, published: March 10, 2022

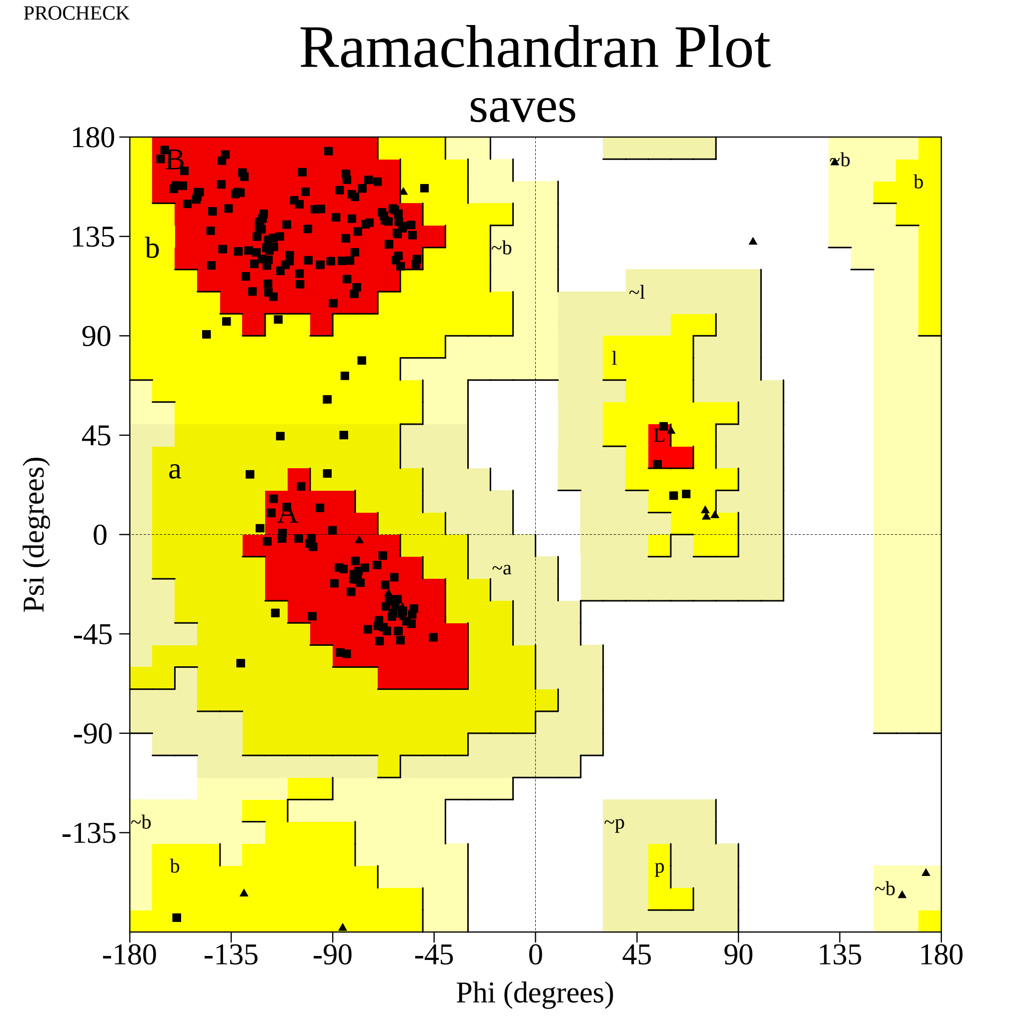

Plot statistics

Residues in most favoured regions [A,B,L]	155	90.6%
Residues in additional allowed regions [a,b,l,p]	16	9.4%
Residues in generously allowed regions [~a,~b,~l,~p]	0	0.0%
Residues in disallowed regions	0	0.0%
	----	------
Number of non-glycine and non-proline residues	171	100.0%
Number of end-residues (excl. Gly and Pro)	2	
Number of glycine residues (shown as triangles)	15	
Number of proline residues	11	

Total number of residues	199	

Based on an analysis of 118 structures of resolution of at least 2.0 Angstroms and R-factor no greater than 20%, a good quality model would be expected to have over 90% in the most favoured regions.

saves_01.ps

Supplementary Figure 1(L): The PROCHECK Ramachandran plot for the validation of the 3D model of Spike RBD of SARS-CoV-2 B.1.214.2 variant (EPI_ISL_760951)

EXCLI Journal 2022;21:610-620 – ISSN 1611-2156
Received: January 26, 2022, accepted: February 25, 2022, published: March 10, 2022

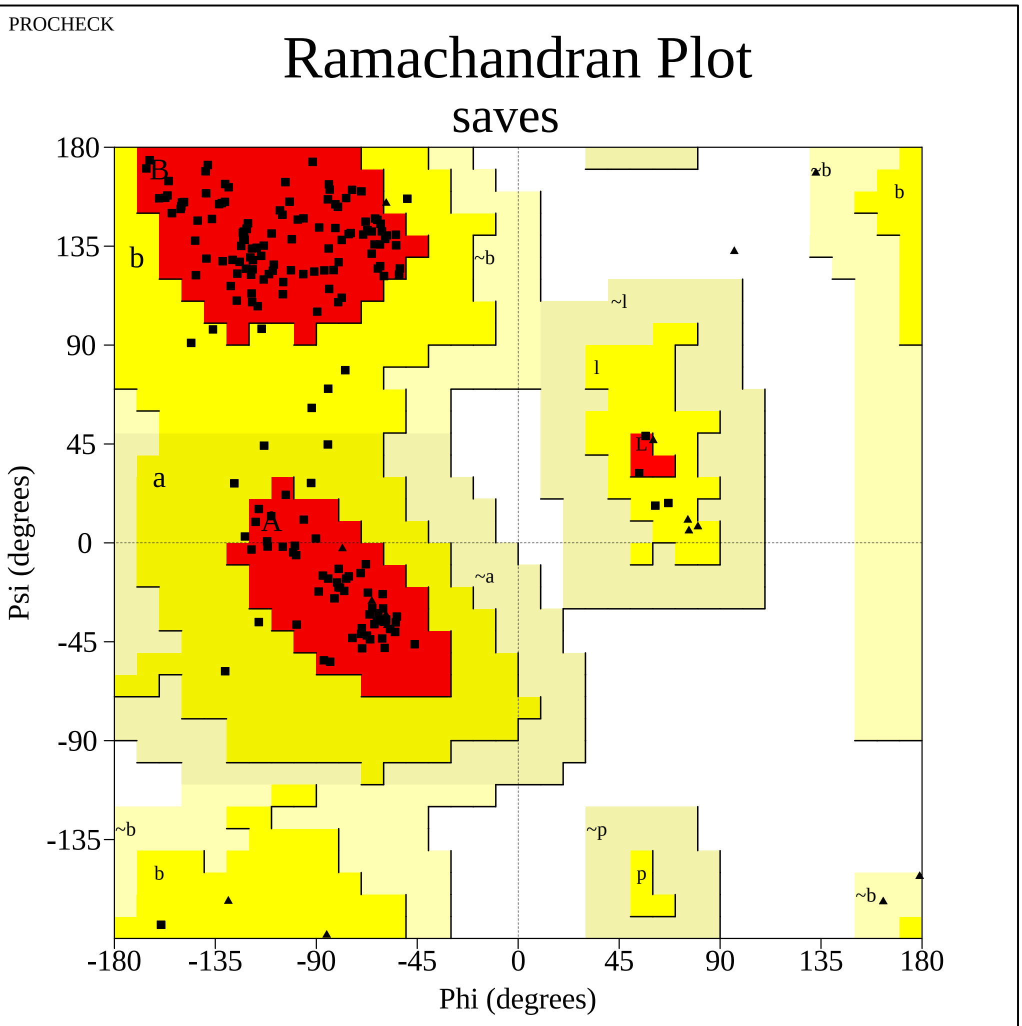

Plot statistics

Residues in most favoured regions [A,B,L]	155	90.6%
Residues in additional allowed regions [a,b,l,p]	16	9.4%
Residues in generously allowed regions [~a,~b,~l,~p]	0	0.0%
Residues in disallowed regions	0	0.0%
	----	------
Number of non-glycine and non-proline residues	171	100.0%
Number of end-residues (excl. Gly and Pro)	2	
Number of glycine residues (shown as triangles)	15	
Number of proline residues	11	

Total number of residues	199	

Based on an analysis of 118 structures of resolution of at least 2.0 Angstroms
and R-factor no greater than 20%, a good quality model would be expected
to have over 90% in the most favoured regions.

saves_01.ps

Supplementary Figure 1(M): The PROCHECK Ramachandran plot for the validation of the 3D model of Spike RBD of SARS-CoV-2 B.1.526(Lota) variant (EPI_ISL_765494)

EXCLI Journal 2022;21:610-620 – ISSN 1611-2156
Received: January 26, 2022, accepted: February 25, 2022, published: March 10, 2022

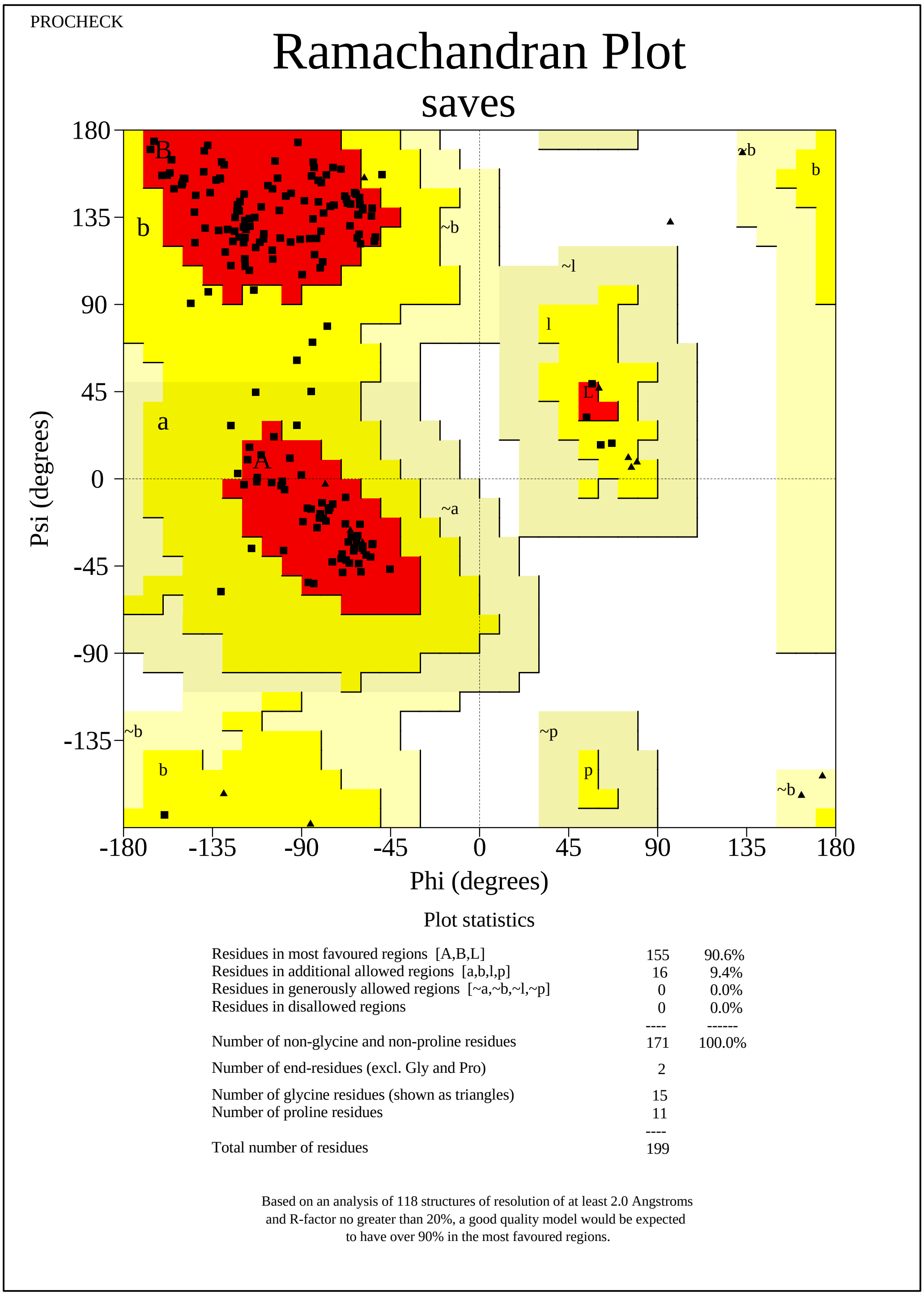

saves_01.ps

Supplementary Figure 1(N): The PROCHECK Ramachandran plot for the validation of the 3D model of Spike RBD of SARS-CoV-2 B.1.466.2 variant (EPI_ISL_877419)

EXCLI Journal 2022;21:610-620 – ISSN 1611-2156
Received: January 26, 2022, accepted: February 25, 2022, published: March 10, 2022

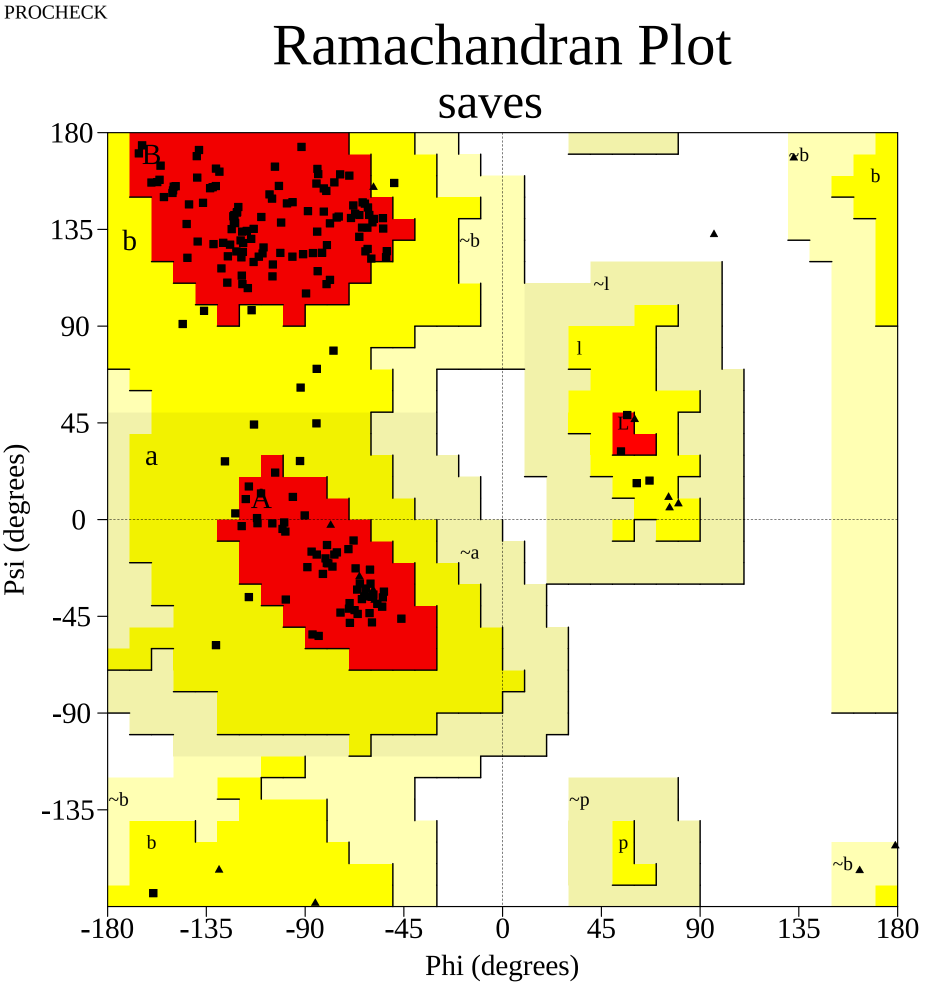

Plot statistics

Residues in most favoured regions [A,B,L]	155	90.6%
Residues in additional allowed regions [a,b,l,p]	16	9.4%
Residues in generously allowed regions [~a,~b,~l,~p]	0	0.0%
Residues in disallowed regions	0	0.0%
	----	------
Number of non-glycine and non-proline residues	171	100.0%
Number of end-residues (excl. Gly and Pro)	2	
Number of glycine residues (shown as triangles)	15	
Number of proline residues	11	

Total number of residues	199	

Based on an analysis of 118 structures of resolution of at least 2.0 Angstroms
and R-factor no greater than 20%, a good quality model would be expected
to have over 90% in the most favoured regions.

saves_01.ps

Supplementary Figure 1(O): The PROCHECK Ramachandran plot for the validation of the 3D model of Spike RBD of SARS-CoV-2 B.1.1.318 variant (EPI_ISL_937654)

EXCLI Journal 2022;21:610-620 – ISSN 1611-2156
Received: January 26, 2022, accepted: February 25, 2022, published: March 10, 2022

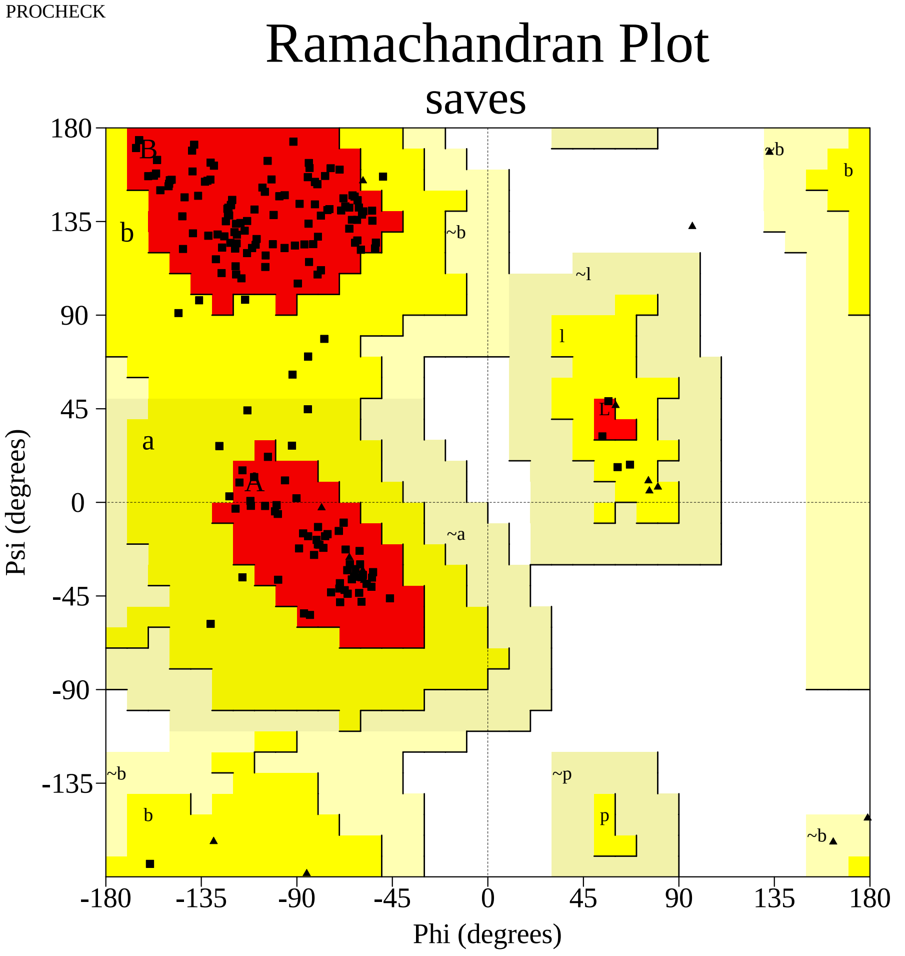

Plot statistics

Residues in most favoured regions [A,B,L]	155	90.6%
Residues in additional allowed regions [a,b,l,p]	16	9.4%
Residues in generously allowed regions [~a,~b,~l,~p]	0	0.0%
Residues in disallowed regions	0	0.0%
	----	------
Number of non-glycine and non-proline residues	171	100.0%
Number of end-residues (excl. Gly and Pro)	2	
Number of glycine residues (shown as triangles)	15	
Number of proline residues	11	

Total number of residues	199	

Based on an analysis of 118 structures of resolution of at least 2.0 Angstroms and R-factor no greater than 20%, a good quality model would be expected to have over 90% in the most favoured regions.

saves_01.ps

Supplementary Figure 1(P): The PROCHECK Ramachandran plot for the validation of the 3D model of Spike RBD of SARS-CoV-2 B.1.619 variant (EPI_ISL_1150929)

EXCLI Journal 2022;21:610-620 – ISSN 1611-2156
Received: January 26, 2022, accepted: February 25, 2022, published: March 10, 2022

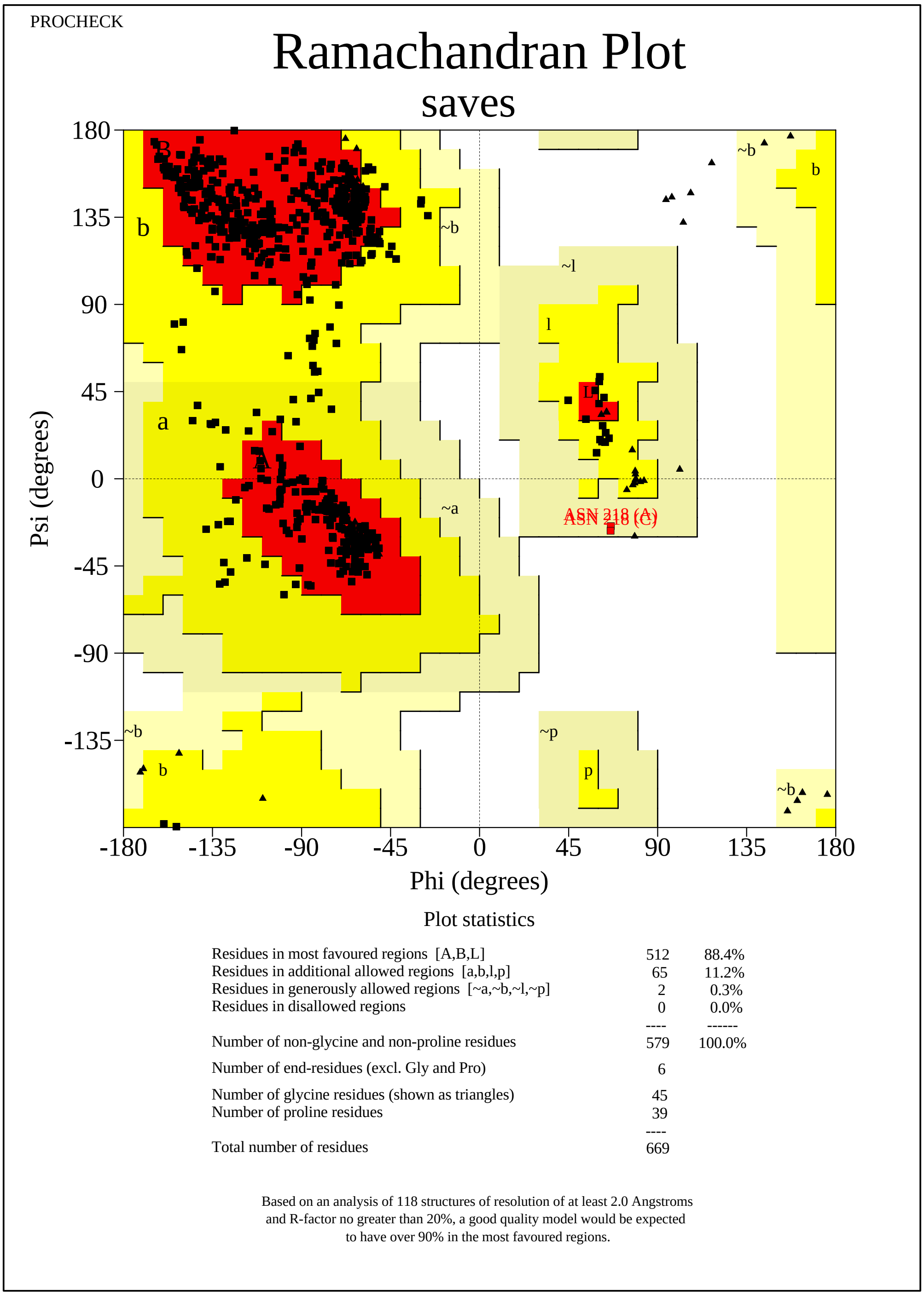

saves_01.ps

Supplementary Figure 1(Q): The PROCHECK Ramachandran plot for the validation of the 3D model of Spike RBD of SARS-CoV-2 C.36.3 variant (EPI_ISL_1237137)

EXCLI Journal 2022;21:610-620 – ISSN 1611-2156
Received: January 26, 2022, accepted: February 25, 2022, published: March 10, 2022

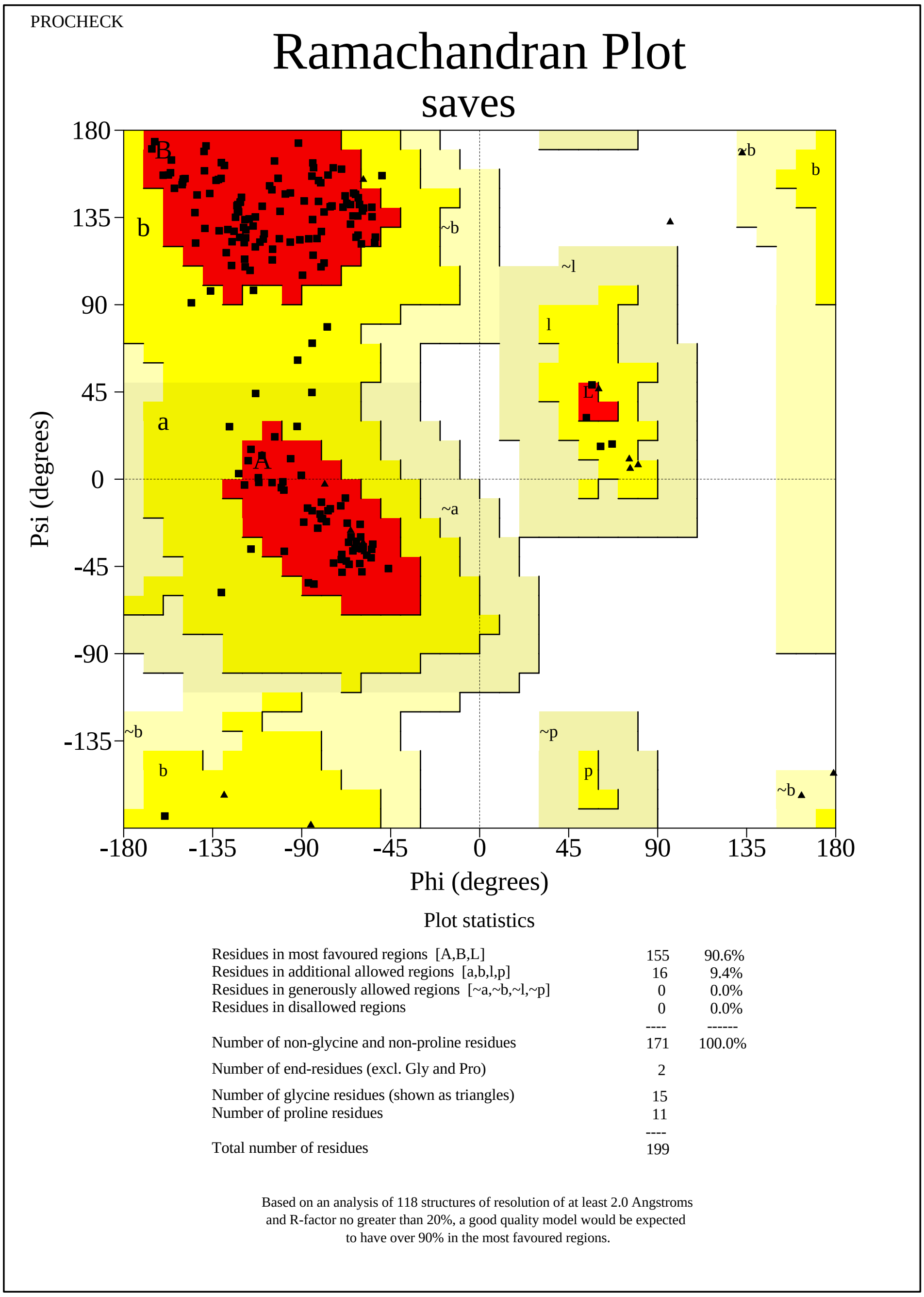

saves_01.ps

Supplementary Figure 1(R): The PROCHECK Ramachandran plot for the validation of the 3D model of Spike RBD of SARS-CoV-2 AT.1 variant (EPI_ISL_1259283)

EXCLI Journal 2022;21:610-620 – ISSN 1611-2156
Received: January 26, 2022, accepted: February 25, 2022, published: March 10, 2022

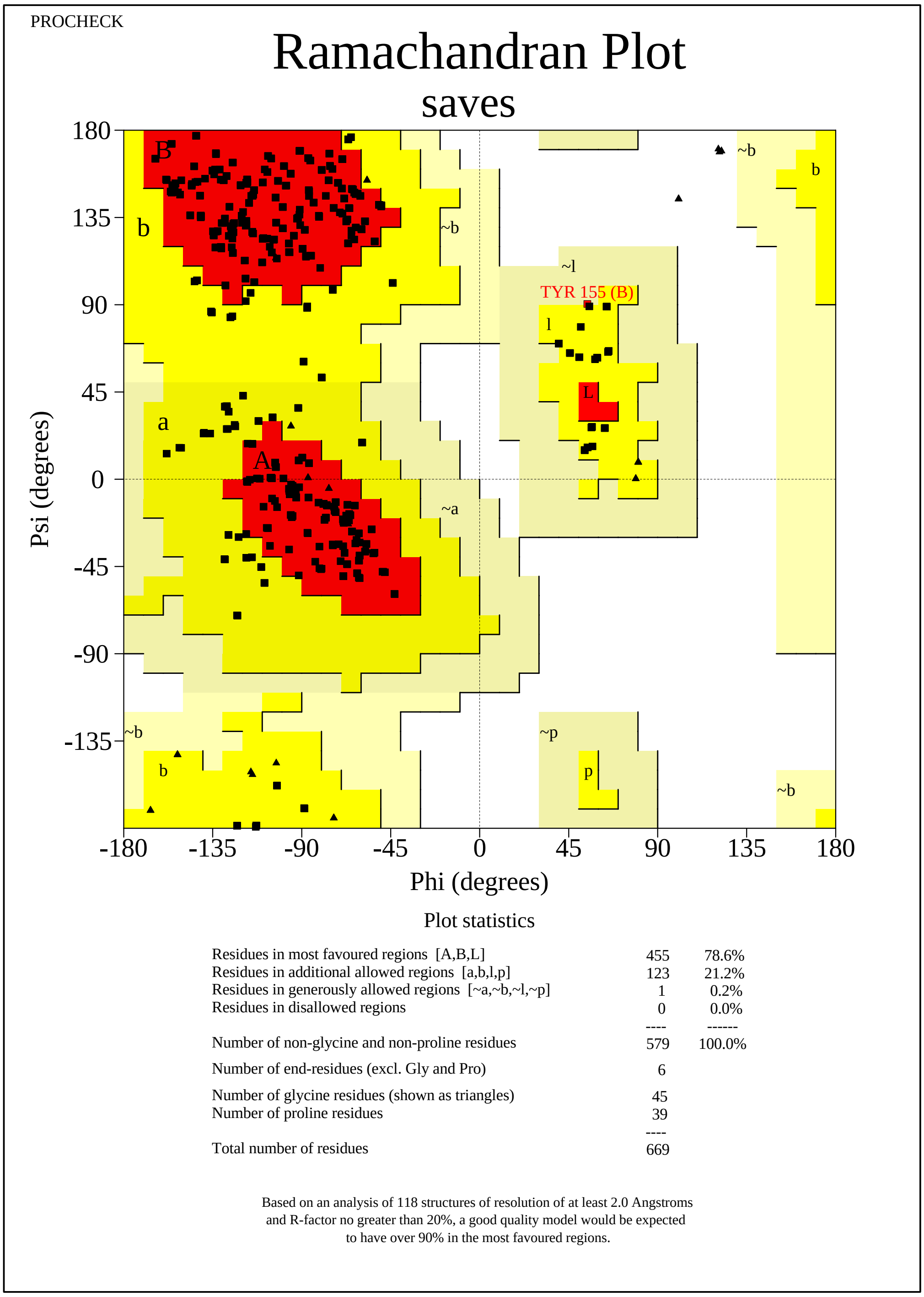

saves_01.ps

Supplementary Figure 1(S): The PROCHECK Ramachandran plot for the validation of the 3D model of Spike RBD of SARS-CoV-2 B.1.617.1(Kappa) variant (EPI_ISL_1357699)

EXCLI Journal 2022;21:610-620 – ISSN 1611-2156
Received: January 26, 2022, accepted: February 25, 2022, published: March 10, 2022

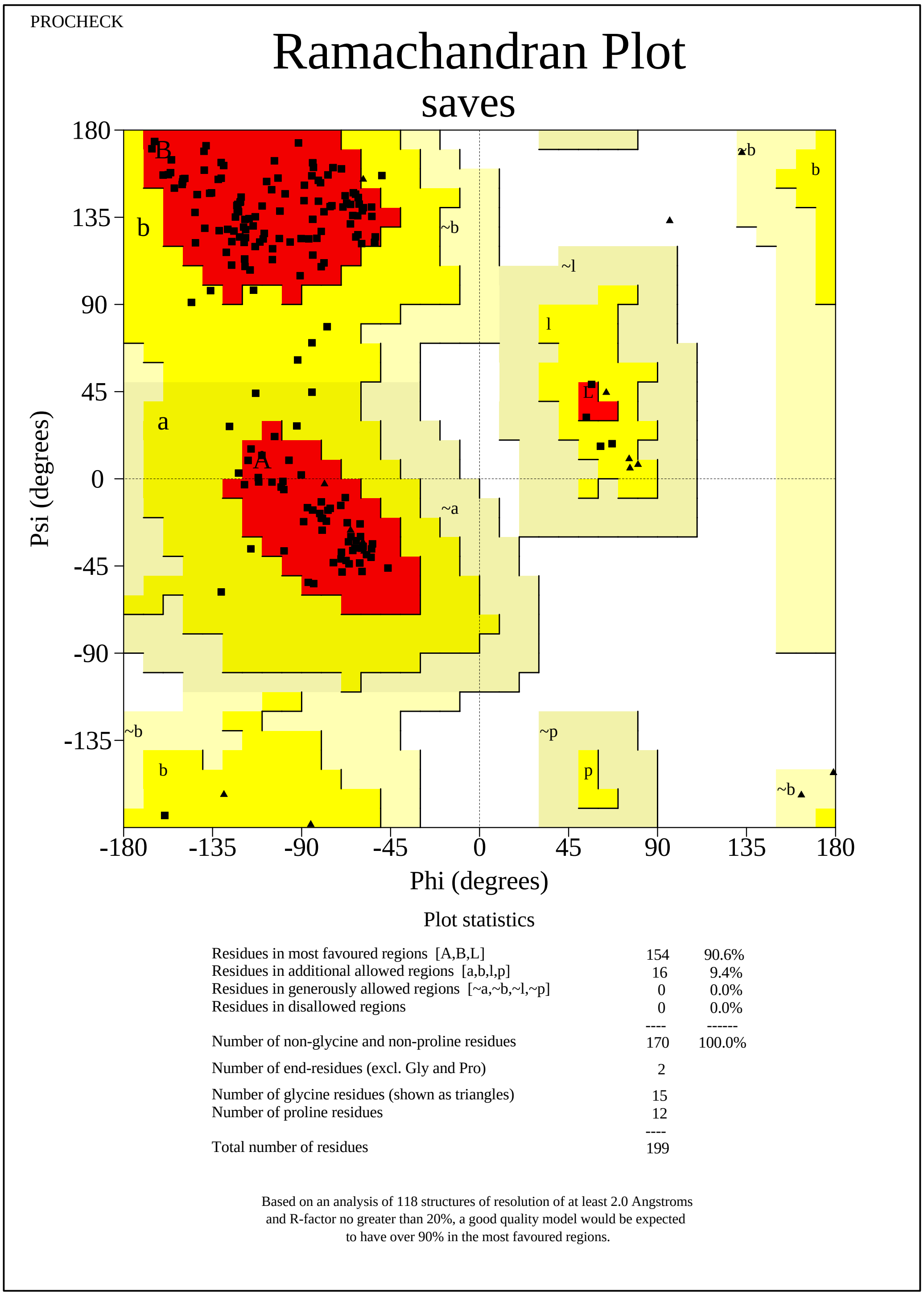

saves_01.ps

Supplementary Figure 1(T): The PROCHECK Ramachandran plot for the validation of the 3D model of Spike RBD of SARS-CoV-2 B.1.1.523 variant (EPI_ISL_1448584)

EXCLI Journal 2022;21:610-620 – ISSN 1611-2156
Received: January 26, 2022, accepted: February 25, 2022, published: March 10, 2022

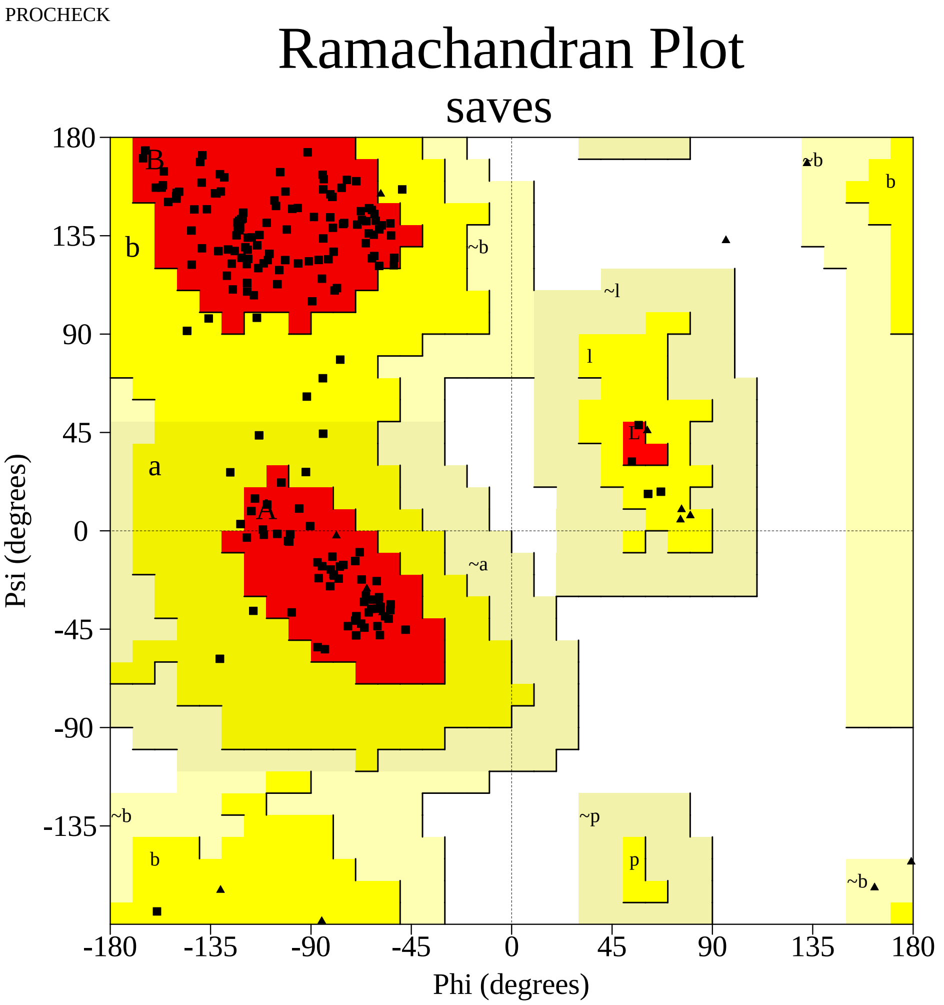

Plot statistics

Residues in most favoured regions [A,B,L]	155	90.6%
Residues in additional allowed regions [a,b,l,p]	16	9.4%
Residues in generously allowed regions [~a,~b,~l,~p]	0	0.0%
Residues in disallowed regions	0	0.0%
	----	------
Number of non-glycine and non-proline residues	171	100.0%
Number of end-residues (excl. Gly and Pro)	2	
Number of glycine residues (shown as triangles)	15	
Number of proline residues	11	

Total number of residues	199	

Based on an analysis of 118 structures of resolution of at least 2.0 Angstroms
and R-factor no greater than 20%, a good quality model would be expected
to have over 90% in the most favoured regions.

saves_01.ps

Supplementary Figure 1(U): The PROCHECK Ramachandran plot for the validation of the 3D model of Spike RBD of SARS-CoV-2 B.1.620 variant (EPI_ISL_1579527)

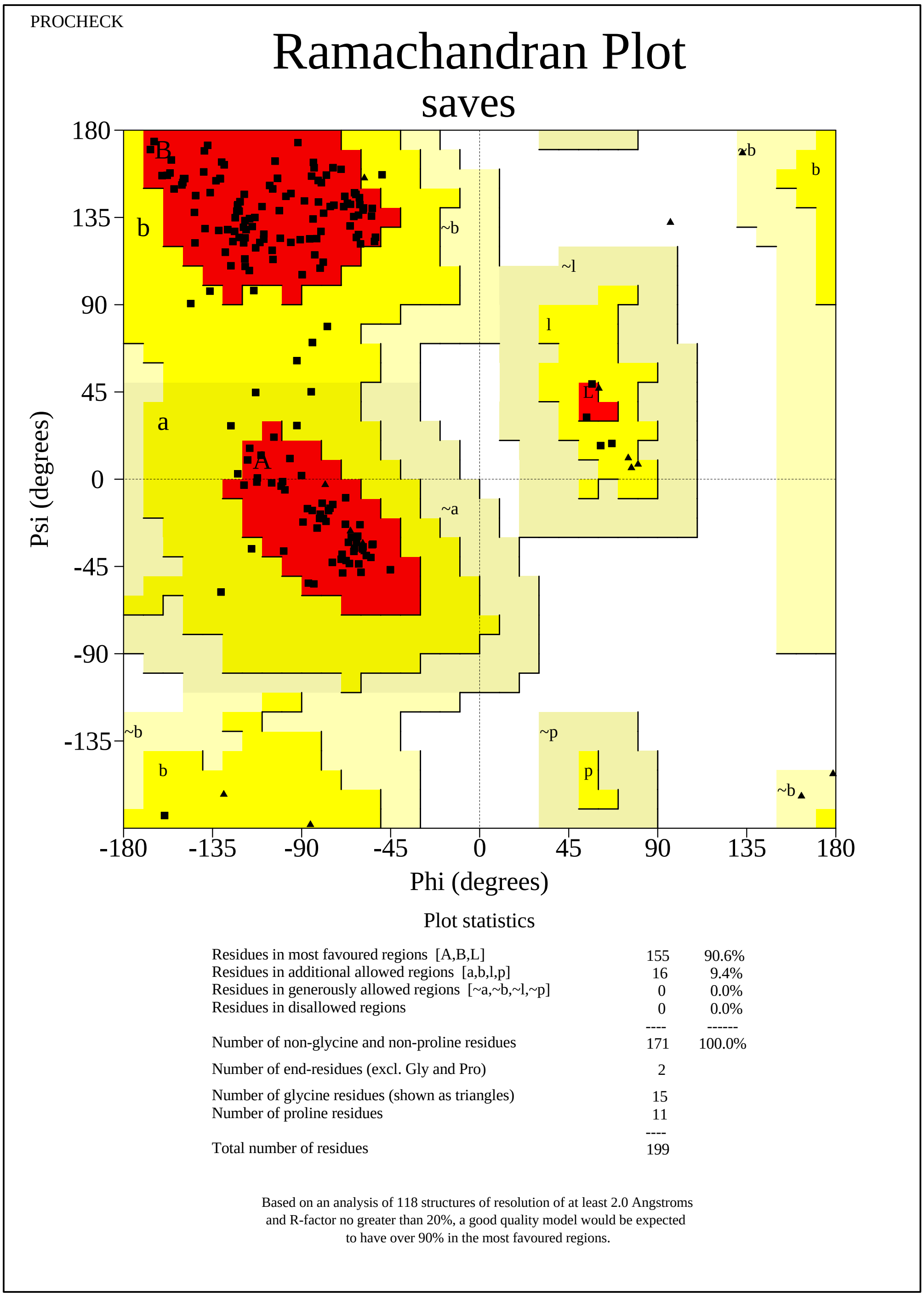

saves_01.ps

Supplementary Figure 1(V): The PROCHECK Ramachandran plot for the validation of the 3D model of Spike RBD of SARS-CoV-2 AV.1 variant (EPI_ISL_1595332)

EXCLI Journal 2022;21:610-620 – ISSN 1611-2156
Received: January 26, 2022, accepted: February 25, 2022, published: March 10, 2022

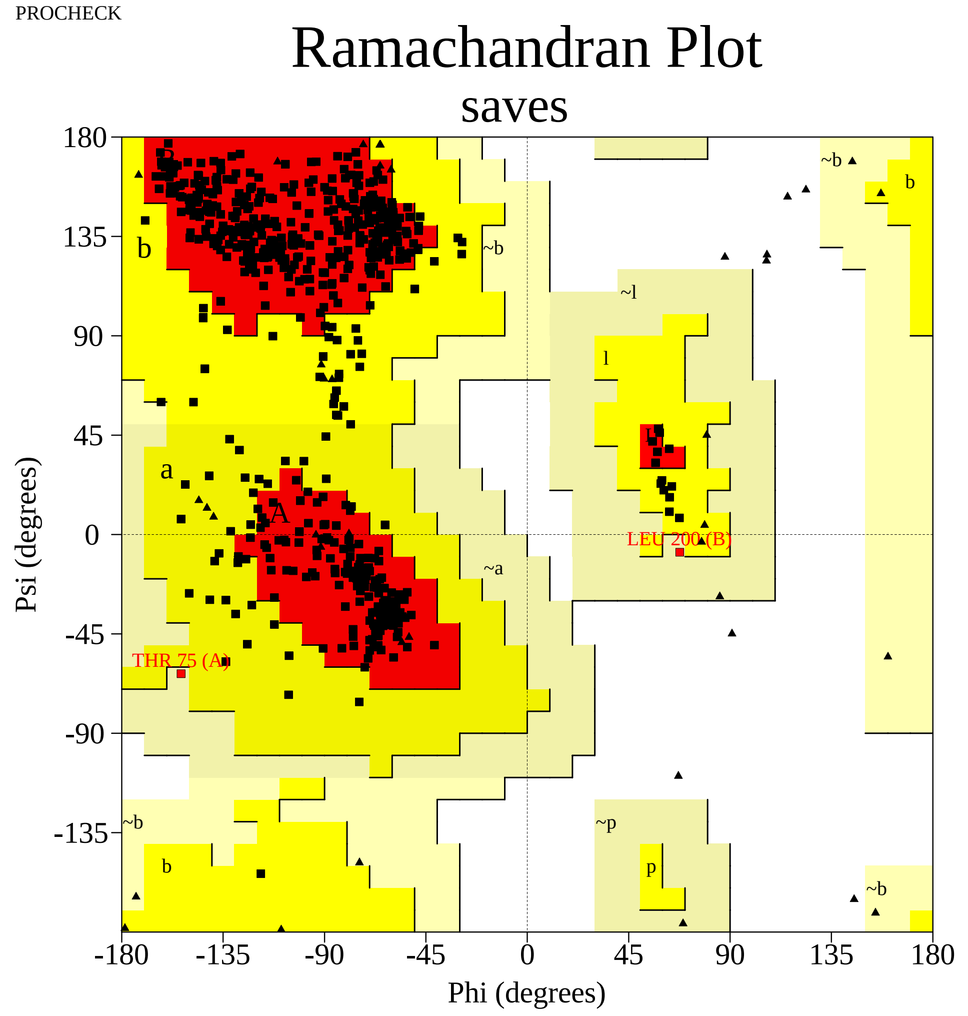

Plot statistics

Residues in most favoured regions [A,B,L]	501	86.5%
Residues in additional allowed regions [a,b,l,p]	76	13.1%
Residues in generously allowed regions [~a,~b,~l,~p]	2	0.3%
Residues in disallowed regions	0	0.0%
	----	------
Number of non-glycine and non-proline residues	579	100.0%
Number of end-residues (excl. Gly and Pro)	6	
Number of glycine residues (shown as triangles)	45	
Number of proline residues	39	

Total number of residues	669	

Based on an analysis of 118 structures of resolution of at least 2.0 Angstroms and R-factor no greater than 20%, a good quality model would be expected to have over 90% in the most favoured regions.

saves_01.ps

Supplementary Figure 1(W): The PROCHECK Ramachandran plot for the validation of the 3D model of Spike RBD of SARS-CoV-2 B.1.630 variant (EPI_ISL_3045385)

EXCLI Journal 2022;21:610-620 – ISSN 1611-2156
Received: January 26, 2022, accepted: February 25, 2022, published: March 10, 2022

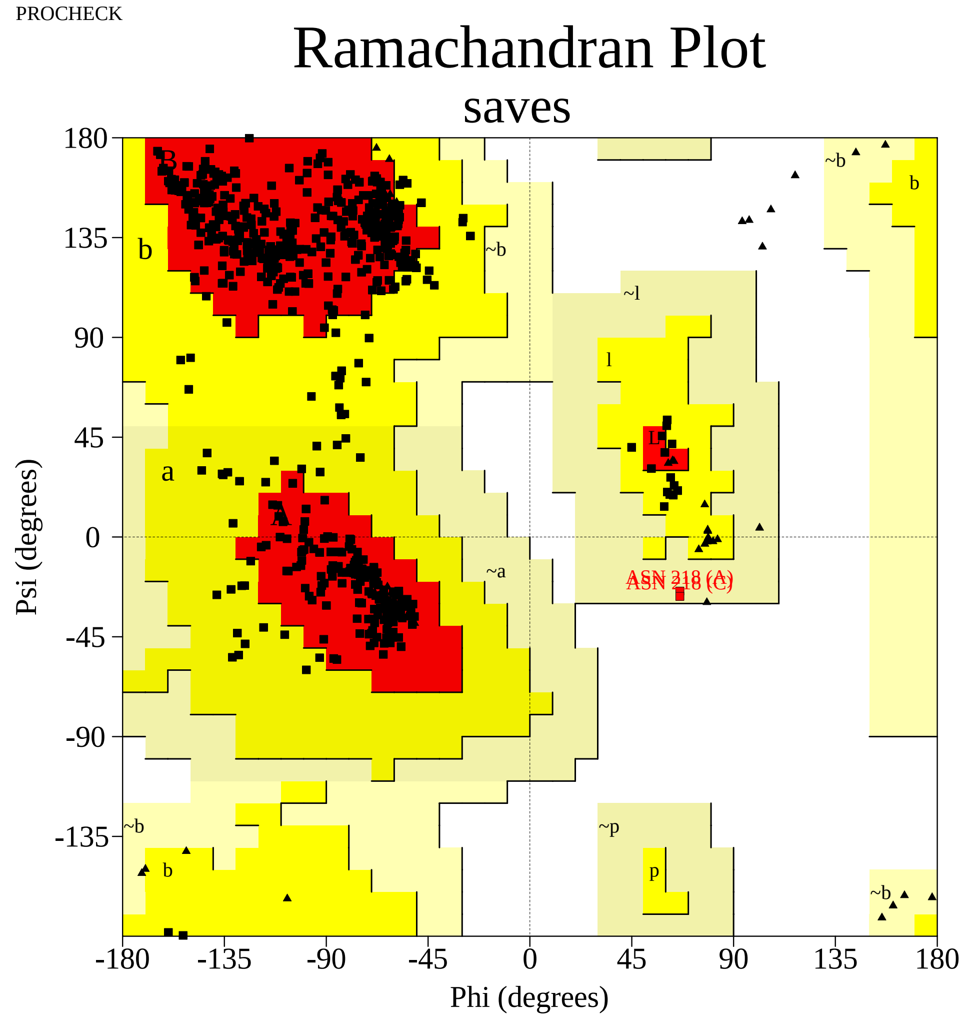

Plot statistics

Residues in most favoured regions [A,B,L]	512	88.4%
Residues in additional allowed regions [a,b,l,p]	65	11.2%
Residues in generously allowed regions [~a,~b,~l,~p]	2	0.3%
Residues in disallowed regions	0	0.0%
	----	------
Number of non-glycine and non-proline residues	579	100.0%
Number of end-residues (excl. Gly and Pro)	6	
Number of glycine residues (shown as triangles)	45	
Number of proline residues	39	

Total number of residues	669	

Based on an analysis of 118 structures of resolution of at least 2.0 Angstroms and R-factor no greater than 20%, a good quality model would be expected to have over 90% in the most favoured regions.

saves_01.ps

Supplementary Figure 1(X): The PROCHECK Ramachandran plot for the validation of the 3D model of Spike RBD of SARS-CoV-2 C.1.2 variant (EPI_ISL_3447714)

EXCLI Journal 2022;21:610-620 – ISSN 1611-2156
Received: January 26, 2022, accepted: February 25, 2022, published: March 10, 2022

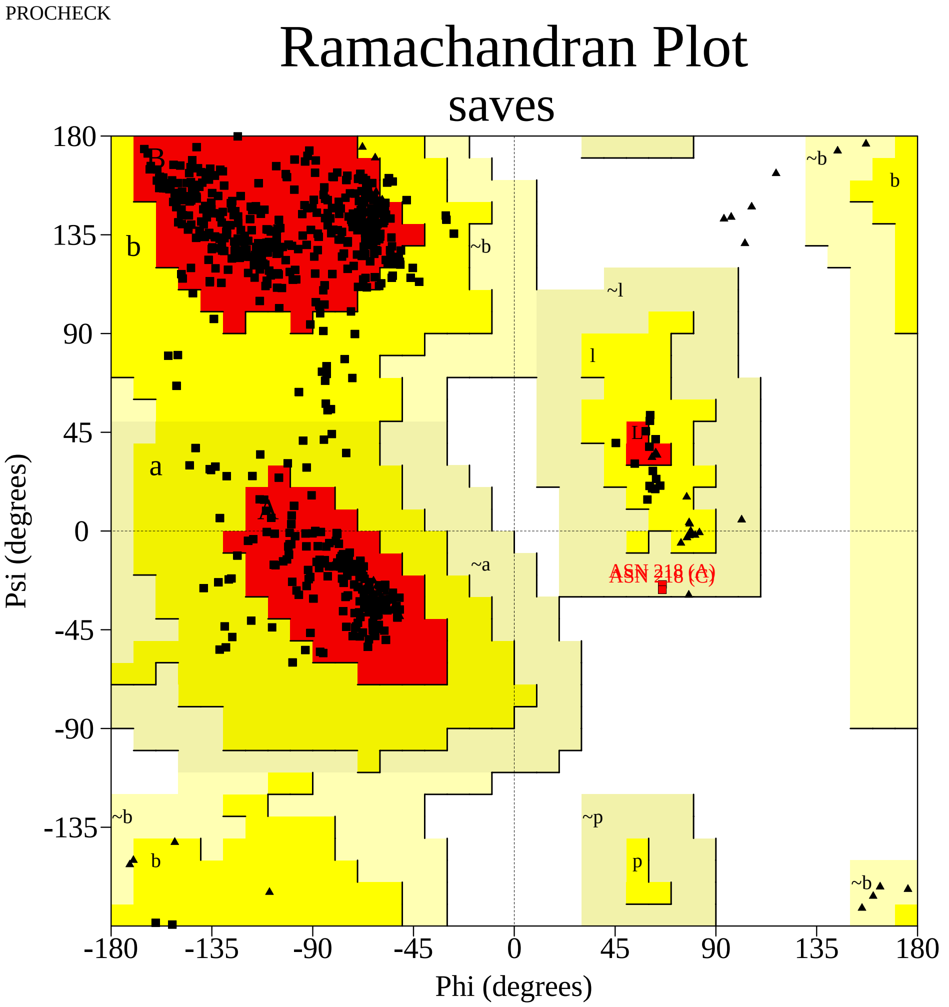

Plot statistics

Residues in most favoured regions [A,B,L]	511	88.3%
Residues in additional allowed regions [a,b,l,p]	66	11.4%
Residues in generously allowed regions [~a,~b,~l,~p]	2	0.3%
Residues in disallowed regions	0	0.0%
	----	------
Number of non-glycine and non-proline residues	579	100.0%
Number of end-residues (excl. Gly and Pro)	6	
Number of glycine residues (shown as triangles)	45	
Number of proline residues	39	

Total number of residues	669	

Based on an analysis of 118 structures of resolution of at least 2.0 Angstroms and R-factor no greater than 20%, a good quality model would be expected to have over 90% in the most favoured regions.

saves_01.ps

Supplementary Figure 1(Y): The PROCHECK Ramachandran plot for the validation of the 3D model of Spike RBD of SARS-CoV-2 B.1.640 variant (EPI_ISL_5592661)

EXCLI Journal 2022;21:610-620 – ISSN 1611-2156
Received: January 26, 2022, accepted: February 25, 2022, published: March 10, 2022

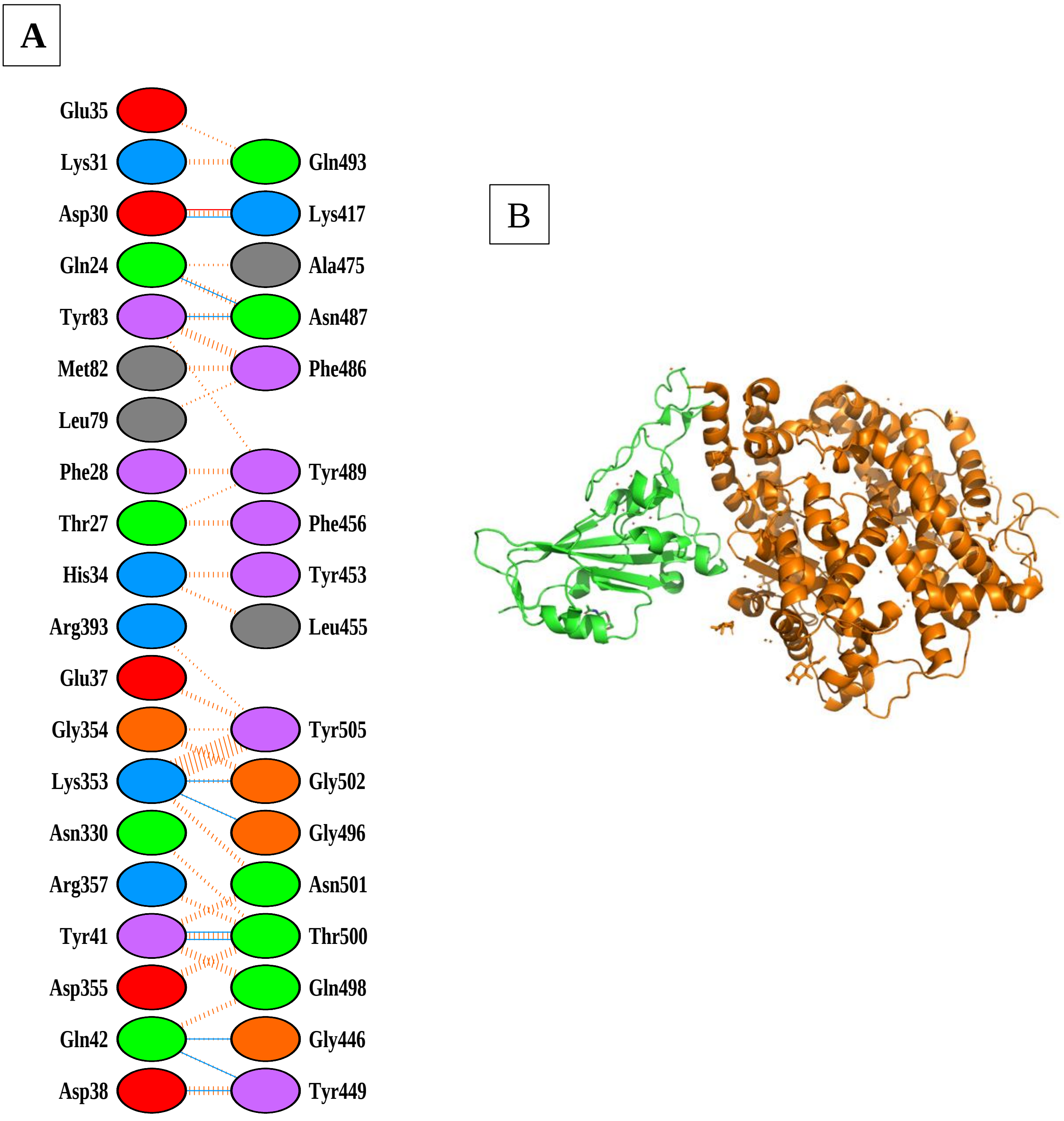

Supplementary Figure 2A: Protein–protein docking representation of hACE2 and RBD of WIV04(EPI_ISL_402124) . (**A**) The interaction representation, which includes hydrogen (▬▬), salt bridges (▬▬), and non-bonded (IIIIIII) interactions. Chain A represents hACE2 and chain B represents RBD. Residue colors: positive (blue): H,K,R; negative (red): D,E; neutral (green): S,T,N,Q; aliphatic (grey): A,V,L,I,M; aromatic (purple): F,Y,W; Pro & Gly (orange): P,G. (**B**) The binding interface of hACE2:RBD complex

EXCLI Journal 2022;21:610-620 – ISSN 1611-2156
Received: January 26, 2022, accepted: February 25, 2022, published: March 10, 2022

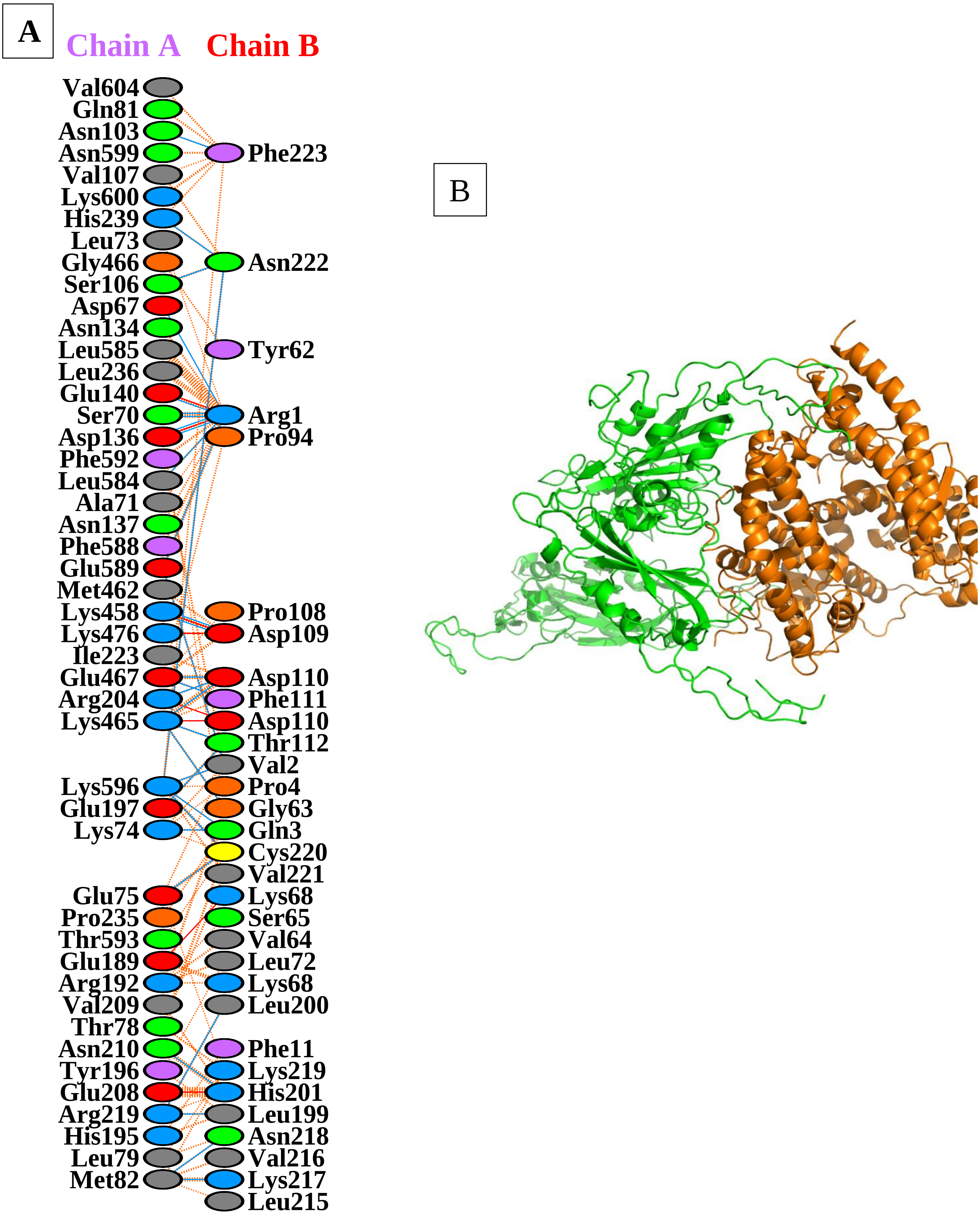

Supplementary Figure 2B: Protein–protein docking representation of hACE2 and RBD of B.1.1.7(Alpha) (EPI_ISL_596982). (**A**) The interaction representation, which includes hydrogen ( ), salt bridges ( ), and non-bonded ( ) interactions. Chain A represents hACE2 and chain B represents RBD. Residue colors: positive (blue): H,K,R; negative (red): D,E; neutral (green): S,T,N,Q; aliphatic (grey): A,V,L,I,M; aromatic (purple): F,Y,W; Pro & Gly (orange): P,G. (**B**) The binding interface of hACE2:RBD complex

EXCLI Journal 2022;21:610-620 – ISSN 1611-2156
Received: January 26, 2022, accepted: February 25, 2022, published: March 10, 2022

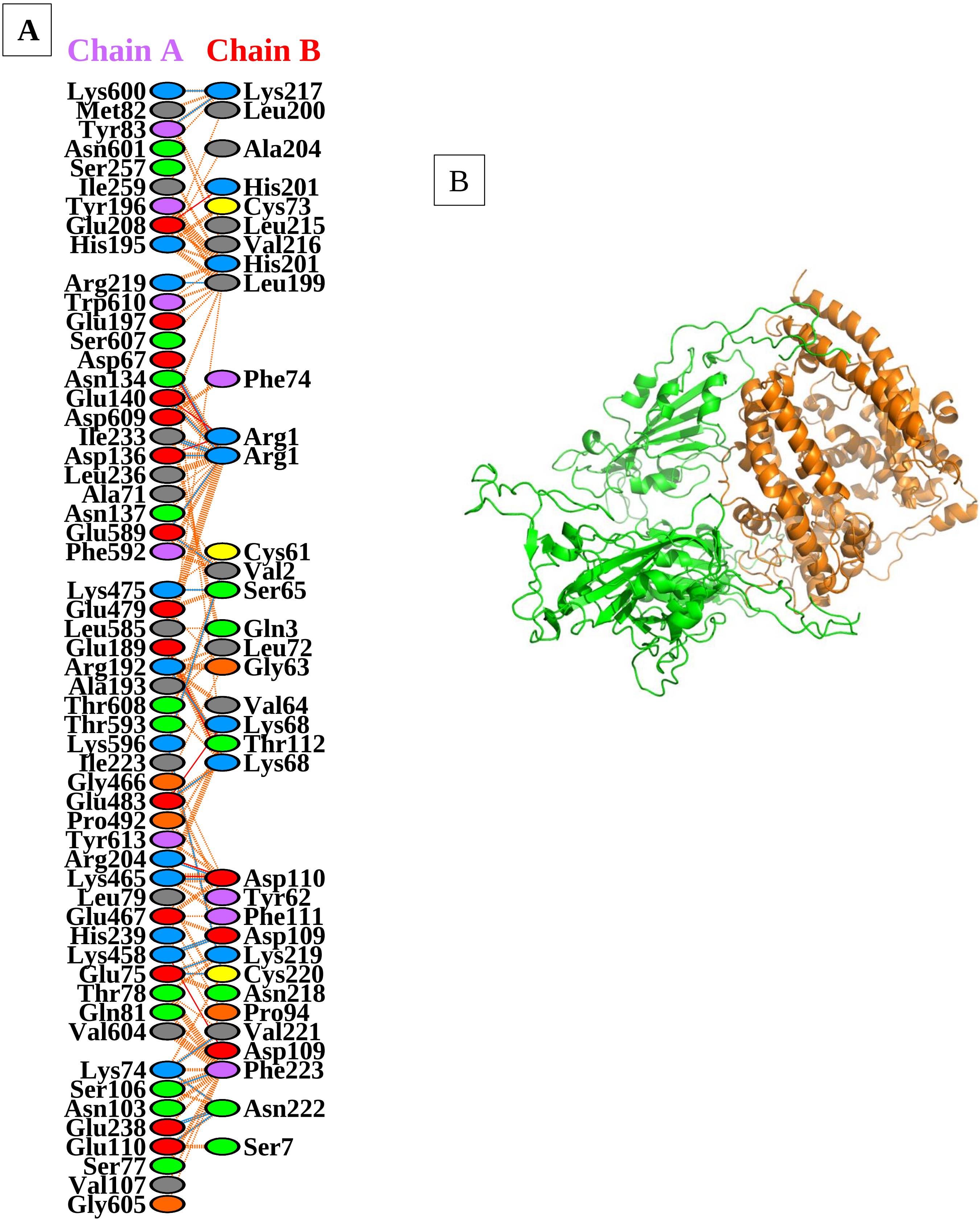

Supplementary Figure 2C: Protein–protein docking representation of hACE2 and RBD of B.1.351(Beta) (EPI_ISL_660629). (**A**) The interaction representation, which includes hydrogen ( ), salt bridges ( ), and non-bonded ( ) interactions. Chain A represents hACE2 and chain B represents RBD. Residue colors: positive (blue): H,K,R; negative (red): D,E; neutral (green): S,T,N,Q; aliphatic (grey): A,V,L,I,M; aromatic (purple): F,Y,W; Pro & Gly (orange): P,G. (**B**) The binding interface of hACE2:RBD complex

EXCLI Journal 2022;21:610-620 – ISSN 1611-2156
Received: January 26, 2022, accepted: February 25, 2022, published: March 10, 2022

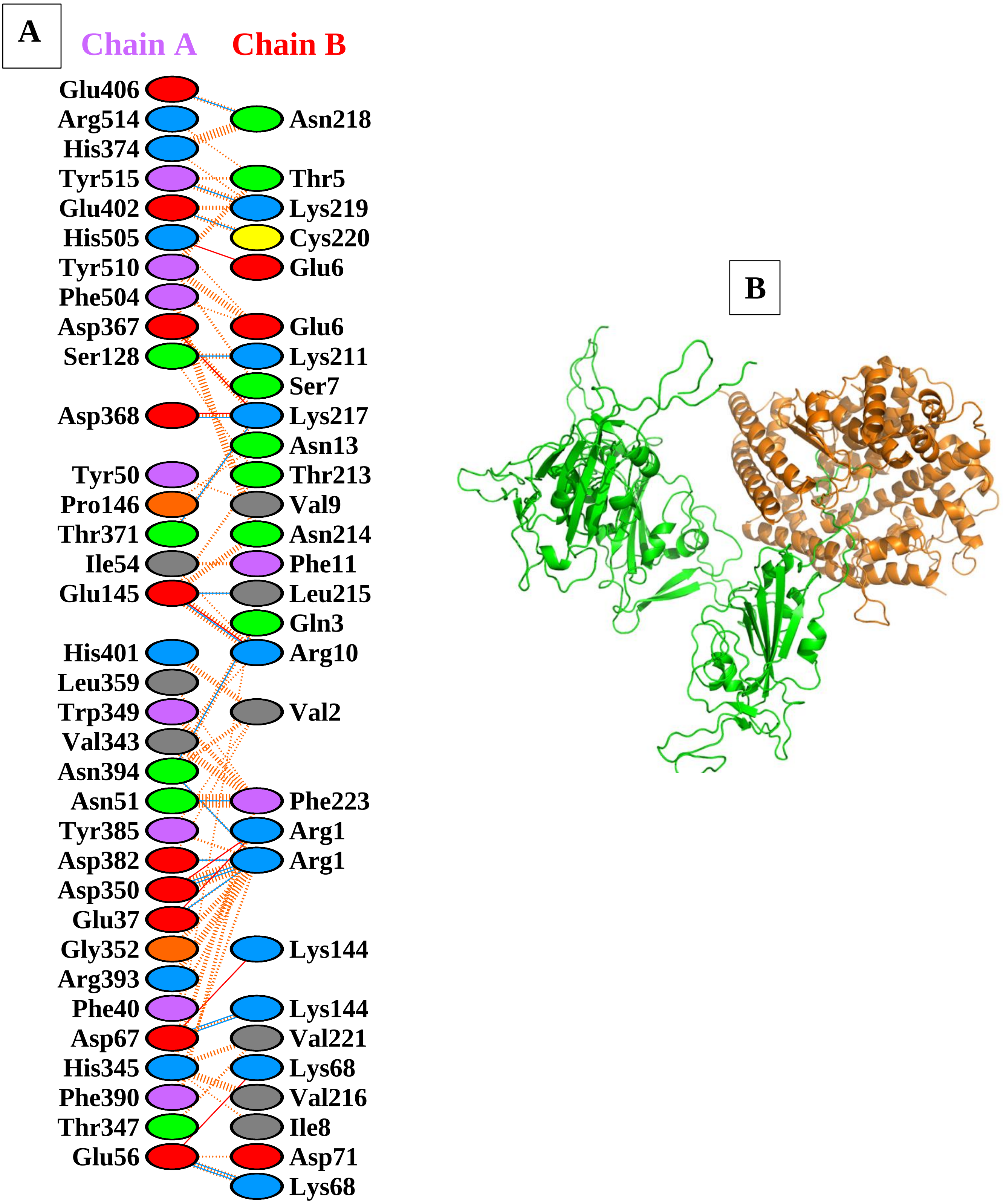

Supplementary Figure 2D: Protein–protein docking representation of hACE2 and RBD of B.1.1.28.1 (Gamma) (EPI_ISL_811149). (**A**) The interaction representation, which includes hydrogen (▬▬), salt bridges (▬▬), and non-bonded (IIIIIII) interactions. Chain A represents hACE2 and chain B represents RBD. Residue colors: positive (blue): H,K,R; negative (red): D,E; neutral (green): S,T,N,Q; aliphatic (grey): A,V,L,I,M; aromatic (purple): F,Y,W; Pro & Gly (orange): P,G. (**B**) The binding interface of hACE2:RBD complex

EXCLI Journal 2022;21:610-620 – ISSN 1611-2156
Received: January 26, 2022, accepted: February 25, 2022, published: March 10, 2022

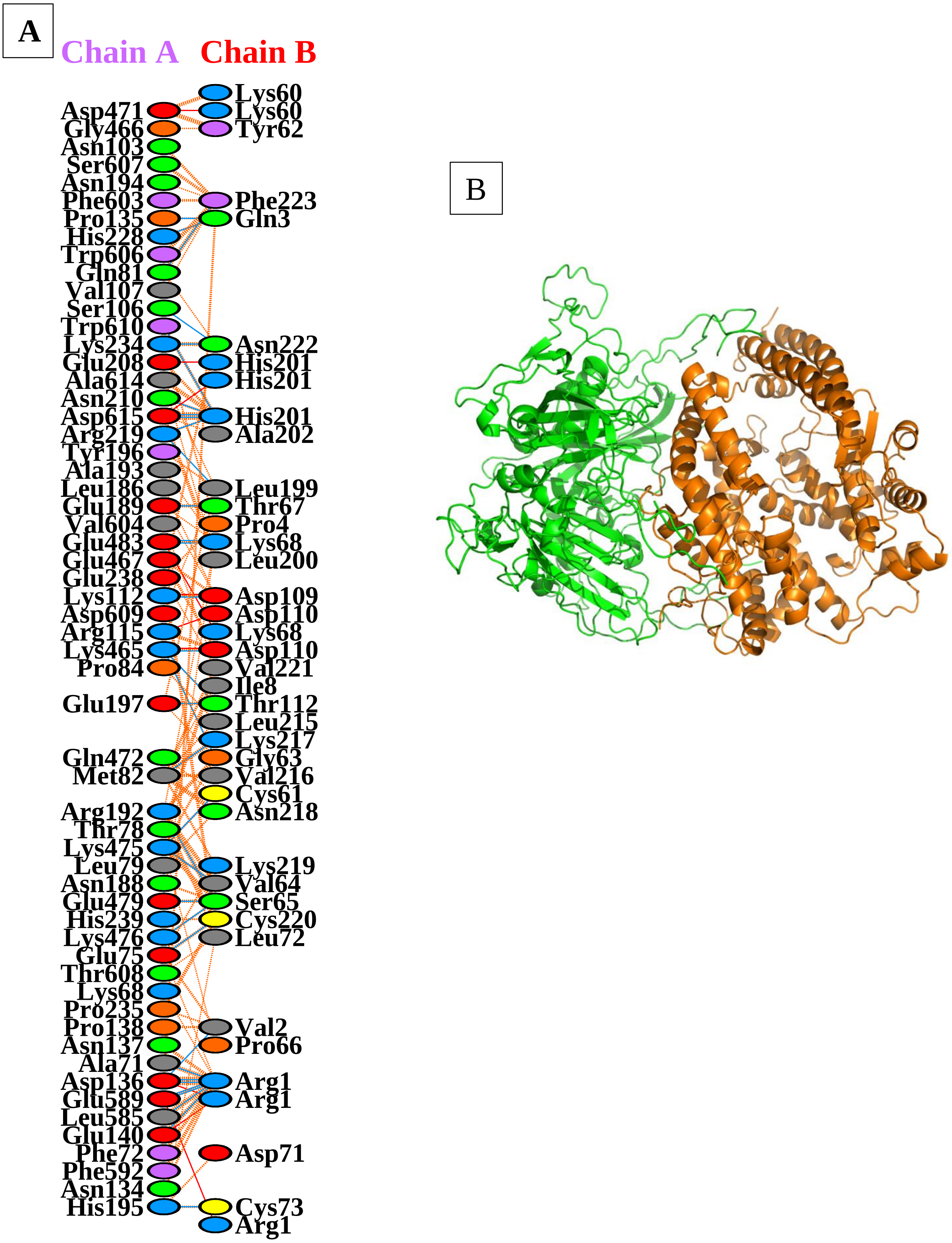

Supplementary Figure 2E: Protein–protein docking representation of hACE2 and RBD of B.1.617(Delta) (EPI_ISL_1360328). (**A**) The interaction representation, which includes hydrogen (▬), salt bridges (▬), and non-bonded (IIIIIII) interactions. Chain A represents hACE2 and chain B represents RBD. Residue colors: positive (blue): H,K,R; negative (red): D,E; neutral (green): S,T,N,Q; aliphatic (grey): A,V,L,I,M; aromatic (purple): F,Y,W; Pro & Gly (orange): P,G. (**B**) The binding interface of hACE2:RBD complex

EXCLI Journal 2022;21:610-620 – ISSN 1611-2156
Received: January 26, 2022, accepted: February 25, 2022, published: March 10, 2022

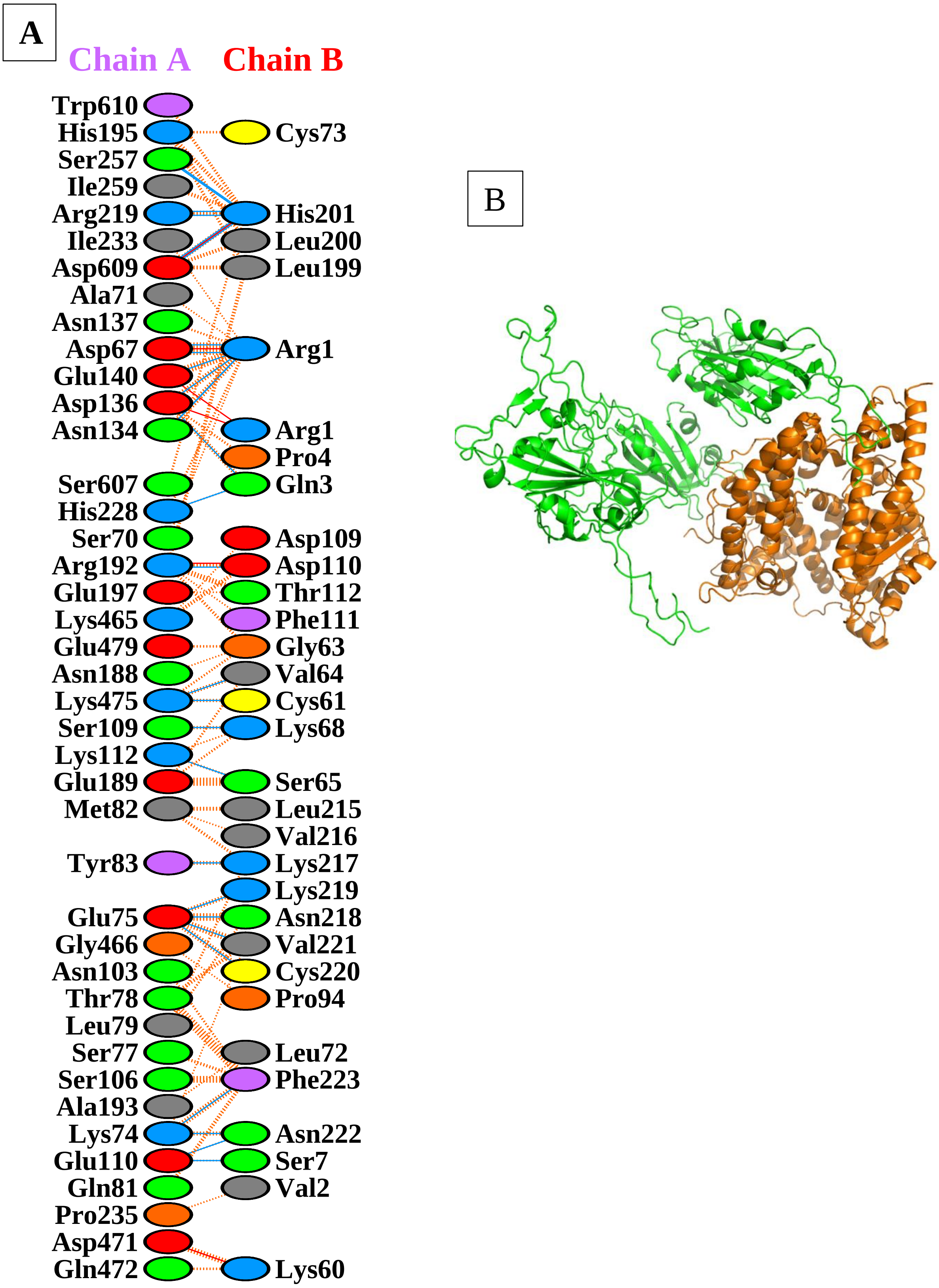

Supplementary Figure 2F: Protein–protein docking representation of hACE2 and RBD of B.1.1.529 (Omicron) (EPI_ISL_6640917). (**A**) The interaction representation, which includes hydrogen (▬▬), salt bridges (▬▬), and non-bonded (||||||) interactions. Chain A represents hACE2 and chain B represents RBD. Residue colors: positive (blue): H,K,R; negative (red): D,E; neutral (green): S,T,N,Q; aliphatic (grey): A,V,L,I,M; aromatic (purple): F,Y,W; Pro & Gly (orange): P,G. (**B**) The binding interface of hACE2:RBD complex

EXCLI Journal 2022;21:610-620 – ISSN 1611-2156
Received: January 26, 2022, accepted: February 25, 2022, published: March 10, 2022

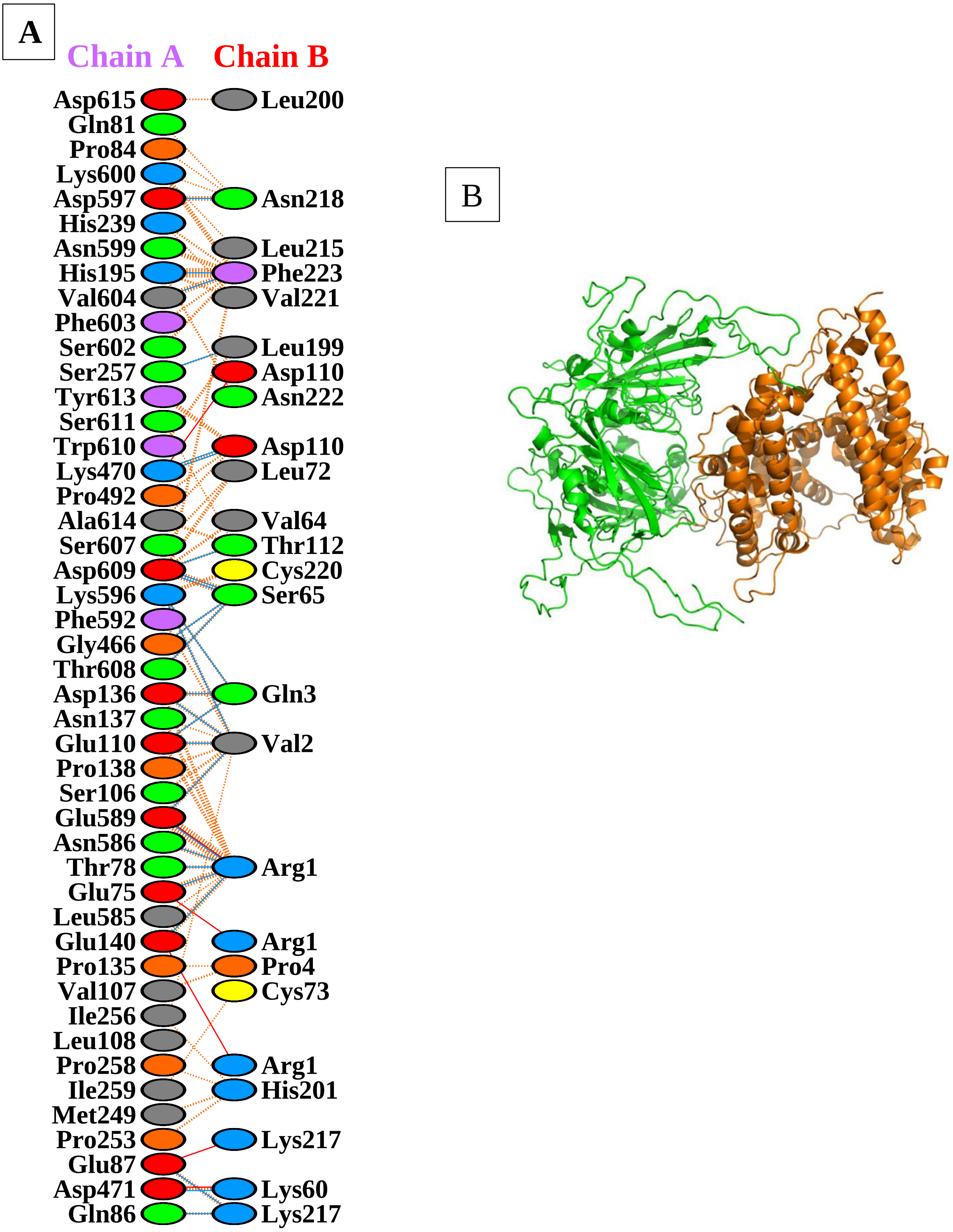

Supplementary Figure 2G: Protein–protein docking representation of hACE2 and RBD of C.37(Lambda) (EPI_ISL_1111334). (**A**) The interaction representation, which includes hydrogen (blue line), salt bridges (red line), and non-bonded (orange dashed line) interactions. Chain A represents hACE2 and chain B represents RBD. Residue colors: positive (blue): H,K,R; negative (red): D,E; neutral (green): S,T,N,Q; aliphatic (grey): A,V,L,I,M; aromatic (purple): F,Y,W; Pro & Gly (orange): P,G. (**B**) The binding interface of hACE2:RBD complex

EXCLI Journal 2022;21:610-620 – ISSN 1611-2156
Received: January 26, 2022, accepted: February 25, 2022, published: March 10, 2022

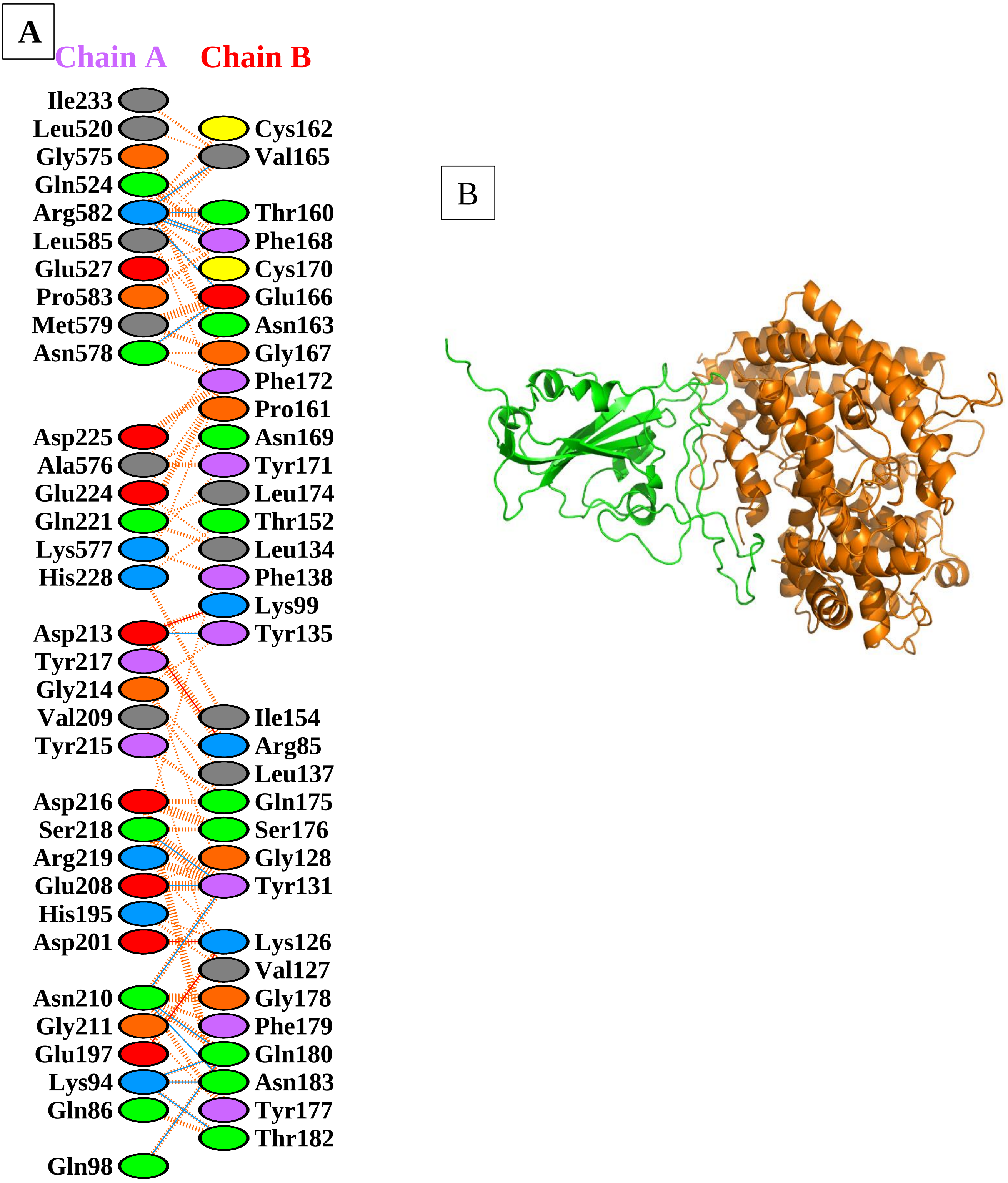

Supplementary Figure 2H: Protein–protein docking representation of hACE2 and RBD of B.1.621(Mu) (EPI_ISL_3369952). (**A**) The interaction representation, which includes hydrogen (▬▬), salt bridges (▬▬), and non-bonded (IIIIIII) interactions. Chain A represents hACE2 and chain B represents RBD. Residue colors: positive (blue): H,K,R; negative (red): D,E; neutral (green): S,T,N,Q; aliphatic (grey): A,V,L,I,M; aromatic (purple): F,Y,W; Pro & Gly (orange): P,G. (**B**) The binding interface of hACE2:RBD complex

Received: January 26, 2022, accepted: February 25, 2022, published: March 10, 2022

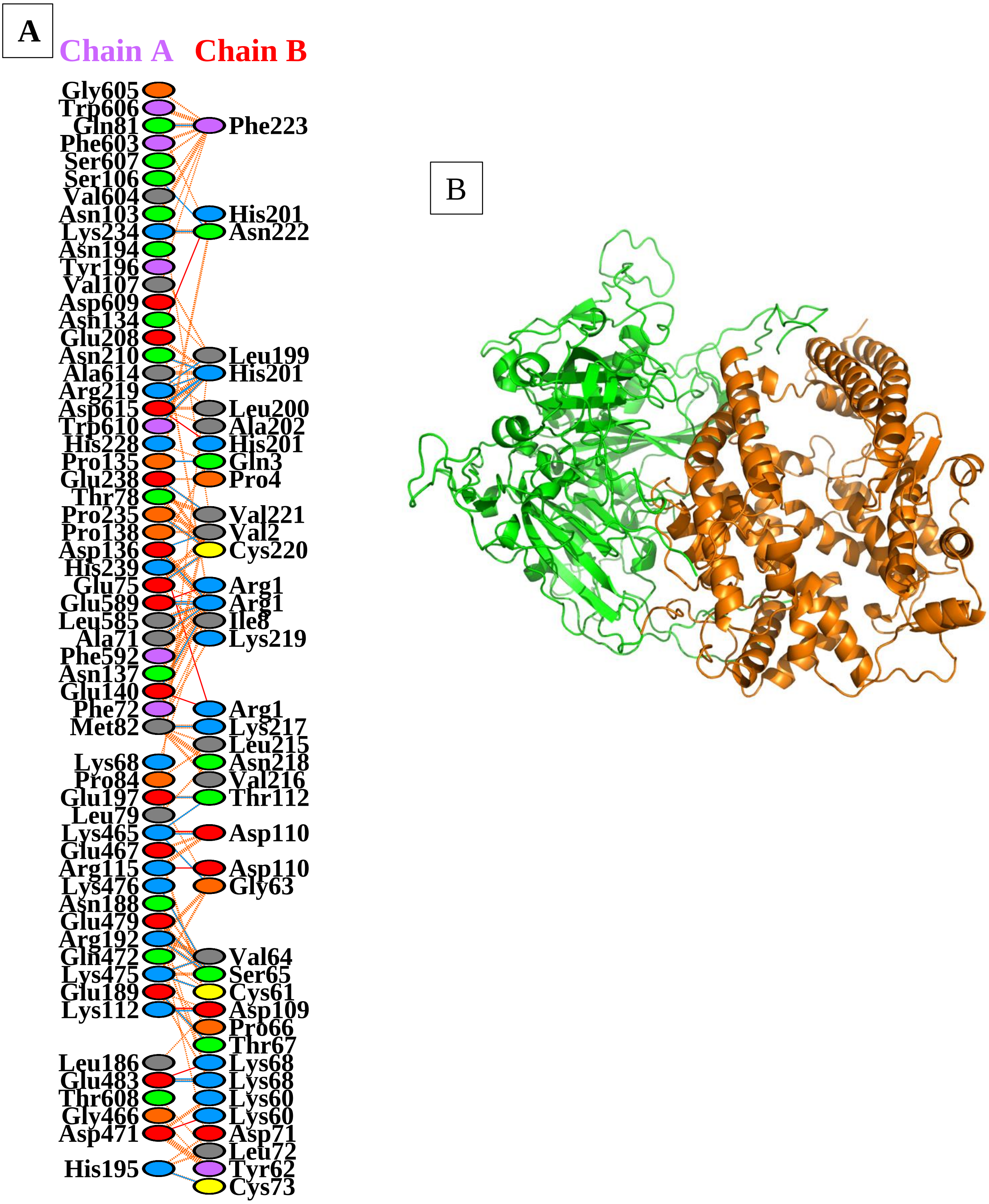

Supplementary Figure 2I: Protein–protein docking representation of hACE2 and RBD of B.1.427/B.1.429 (Epsilon) (EPI_ISL_648527). (**A**) The interaction representation, which includes hydrogen (▬▬), salt bridges (▬▬), and non-bonded (IIIIIII) interactions. Chain A represents hACE2 and chain B represents RBD. Residue colors: positive (blue): H,K,R; negative (red): D,E; neutral (green): S,T,N,Q; aliphatic (grey): A,V,L,I,M; aromatic (purple): F,Y,W; Pro & Gly (orange): P,G. (**B**) The binding interface of hACE2:RBD complex

EXCLI Journal 2022;21:610-620 – ISSN 1611-2156
Received: January 26, 2022, accepted: February 25, 2022, published: March 10, 2022

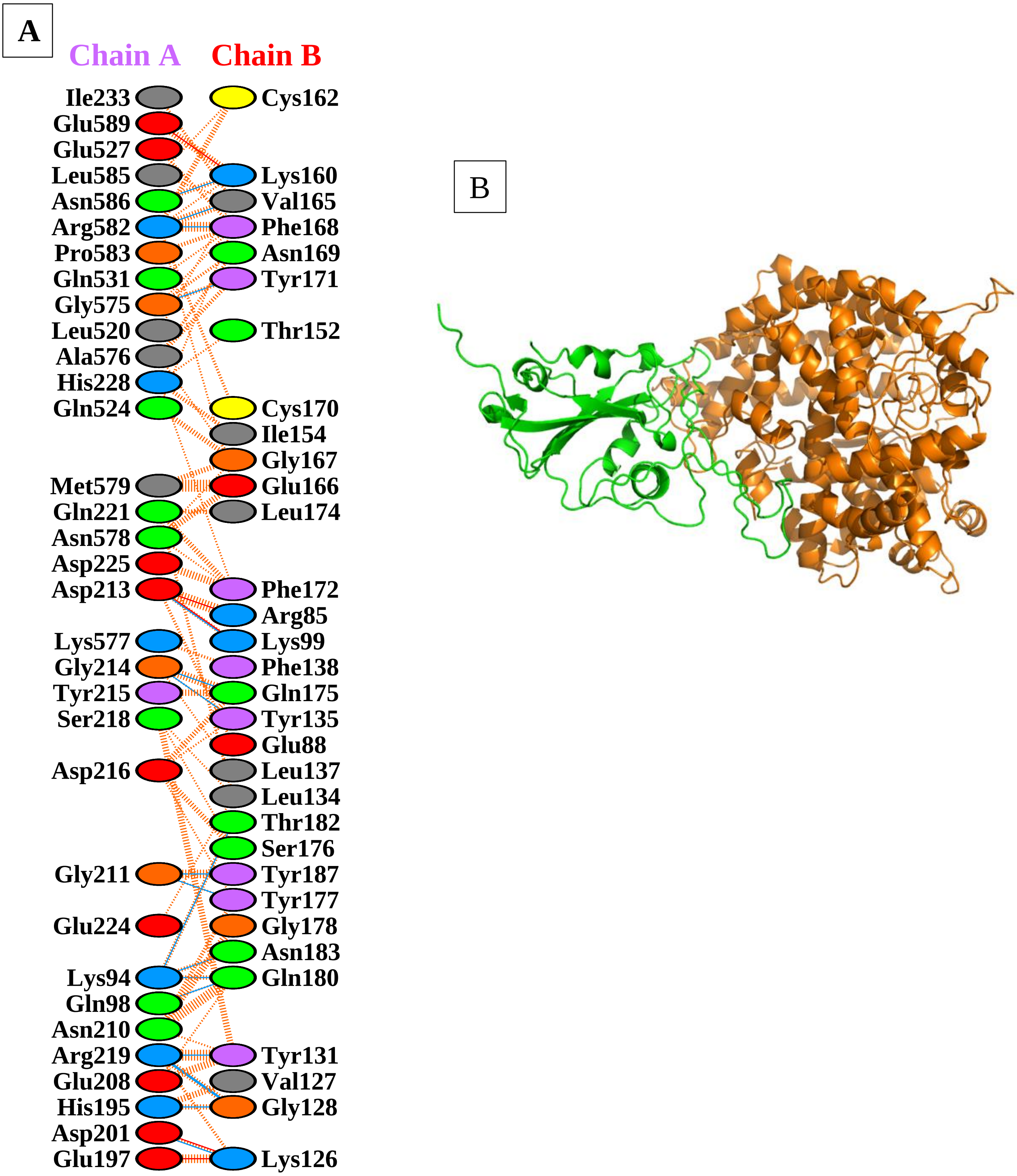

Supplementary Figure 2J: Protein–protein docking representation of hACE2 and RBD of B.1.1.519 (EPI_ISL_721617). (**A**) The interaction representation, which includes hydrogen (▬▬), salt bridges (▬▬), and non-bonded (IIIIIII) interactions. Chain A represents hACE2 and chain B represents RBD. Residue colors: positive (blue): H,K,R; negative (red): D,E; neutral (green): S,T,N,Q; aliphatic (grey): A,V,L,I,M; aromatic (purple): F,Y,W; Pro & Gly (orange): P,G. (**B**) The binding interface of hACE2:RBD complex

Received: January 26, 2022, accepted: February 25, 2022, published: March 10, 2022

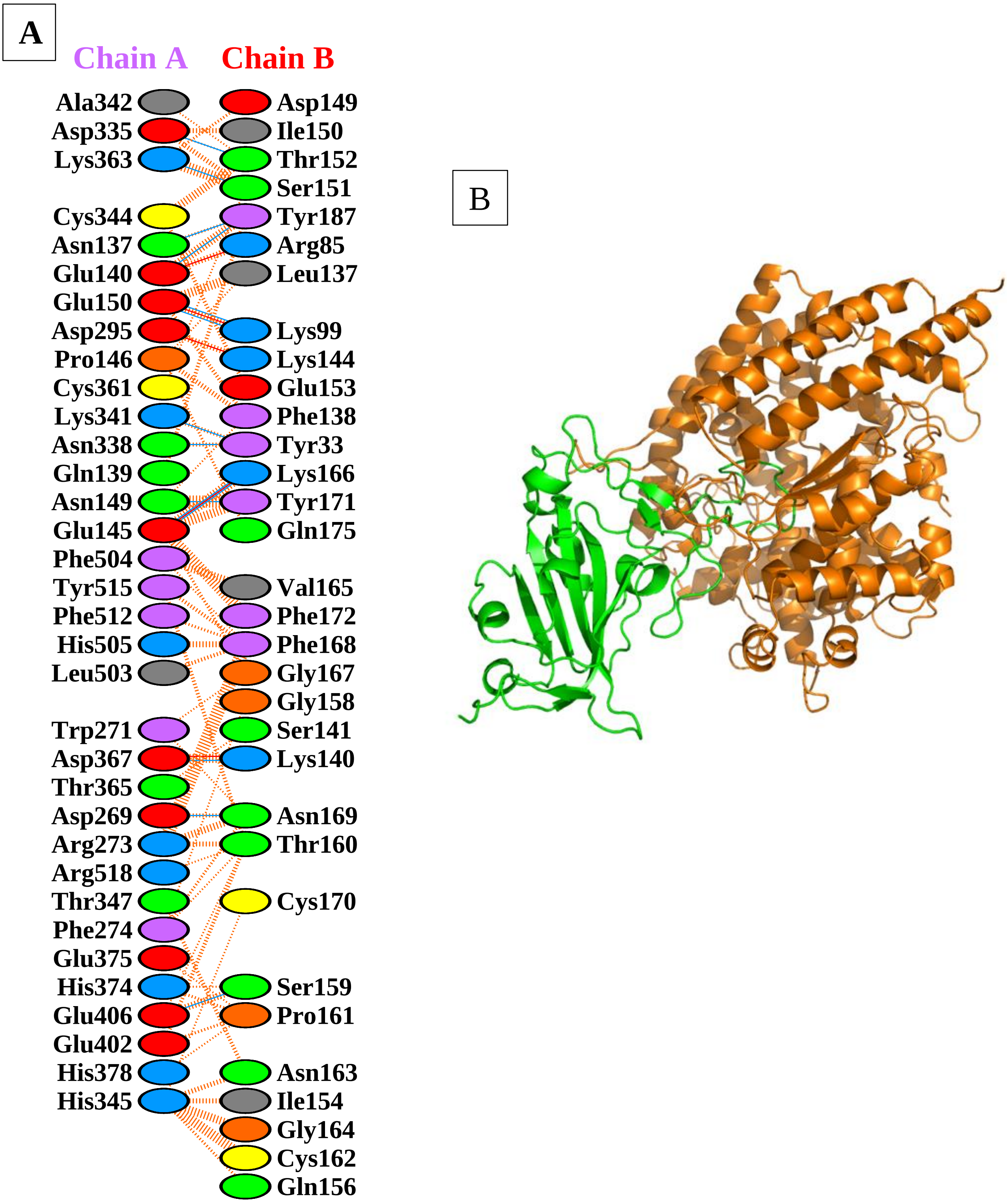

Supplementary Figure 2K: Protein–protein docking representation of hACE2 and RBD of R.1 (EPI_ISL_736897). (**A**) The interaction representation, which includes hydrogen (▬▬), salt bridges (▬▬), and non-bonded (IIIIIII) interactions. Chain A represents hACE2 and chain B represents RBD. Residue colors: positive (blue): H,K,R; negative (red): D,E; neutral (green): S,T,N,Q; aliphatic (grey): A,V,L,I,M; aromatic (purple): F,Y,W; Pro & Gly (orange): P,G. (**B**) The binding interface of hACE2:RBD complex

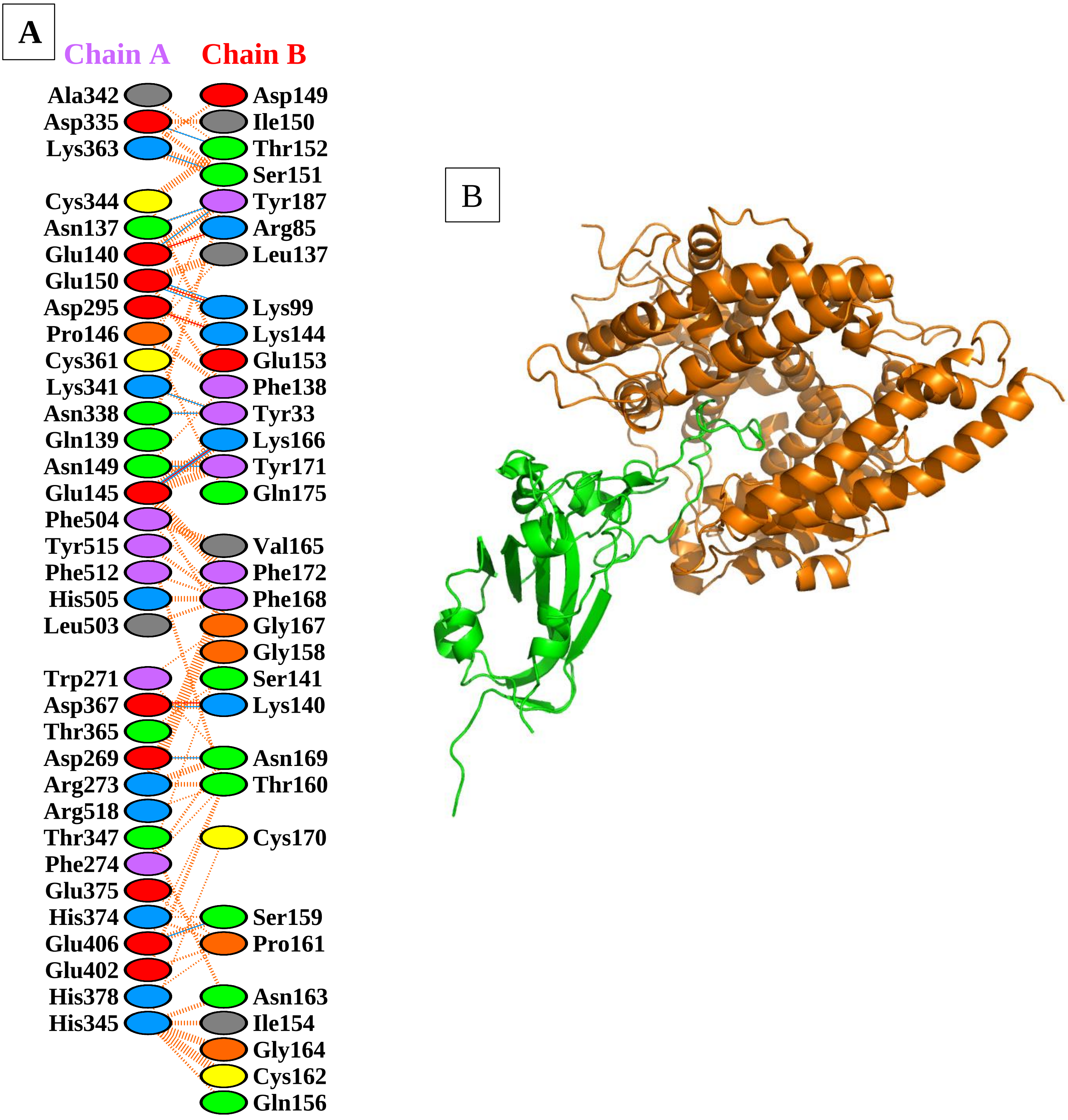

Supplementary Figure 2L: Protein–protein docking representation of hACE2 and RBD of B.1.525(Eta) (EPI_ISL_760883). (**A**) The interaction representation, which includes hydrogen (blue line), salt bridges (red line), and non-bonded (orange dashes) interactions. Chain A represents hACE2 and chain B represents RBD. Residue colors: positive (blue): H,K,R; negative (red): D,E; neutral (green): S,T,N,Q; aliphatic (grey): A,V,L,I,M; aromatic (purple): F,Y,W; Pro & Gly (orange): P,G. (**B**) The binding interface of hACE2:RBD complex

EXCLI Journal 2022;21:610-620 – ISSN 1611-2156
Received: January 26, 2022, accepted: February 25, 2022, published: March 10, 2022

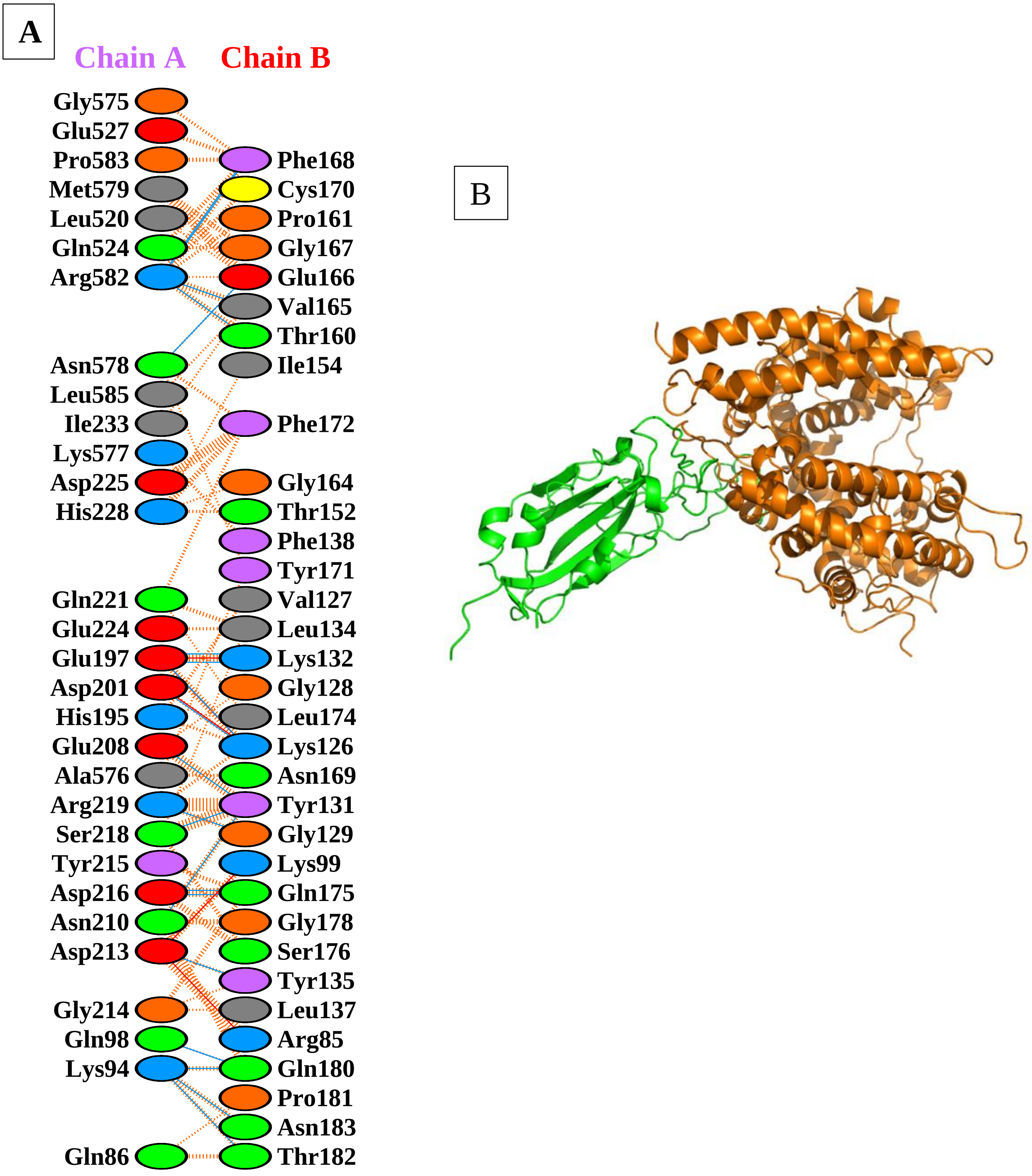

Supplementary Figure 2M: Protein–protein docking representation of hACE2 and RBD of B.1.214.2 (EPI_ISL_760951). (**A**) The interaction representation, which includes hydrogen (blue line), salt bridges (red line), and non-bonded (orange dashes) interactions. Chain A represents hACE2 and chain B represents RBD. Residue colors: positive (blue): H,K,R; negative (red): D,E; neutral (green): S,T,N,Q; aliphatic (grey): A,V,L,I,M; aromatic (purple): F,Y,W; Pro & Gly (orange): P,G. (**B**) The binding interface of hACE2:RBD complex

EXCLI Journal 2022;21:610-620 – ISSN 1611-2156
Received: January 26, 2022, accepted: February 25, 2022, published: March 10, 2022

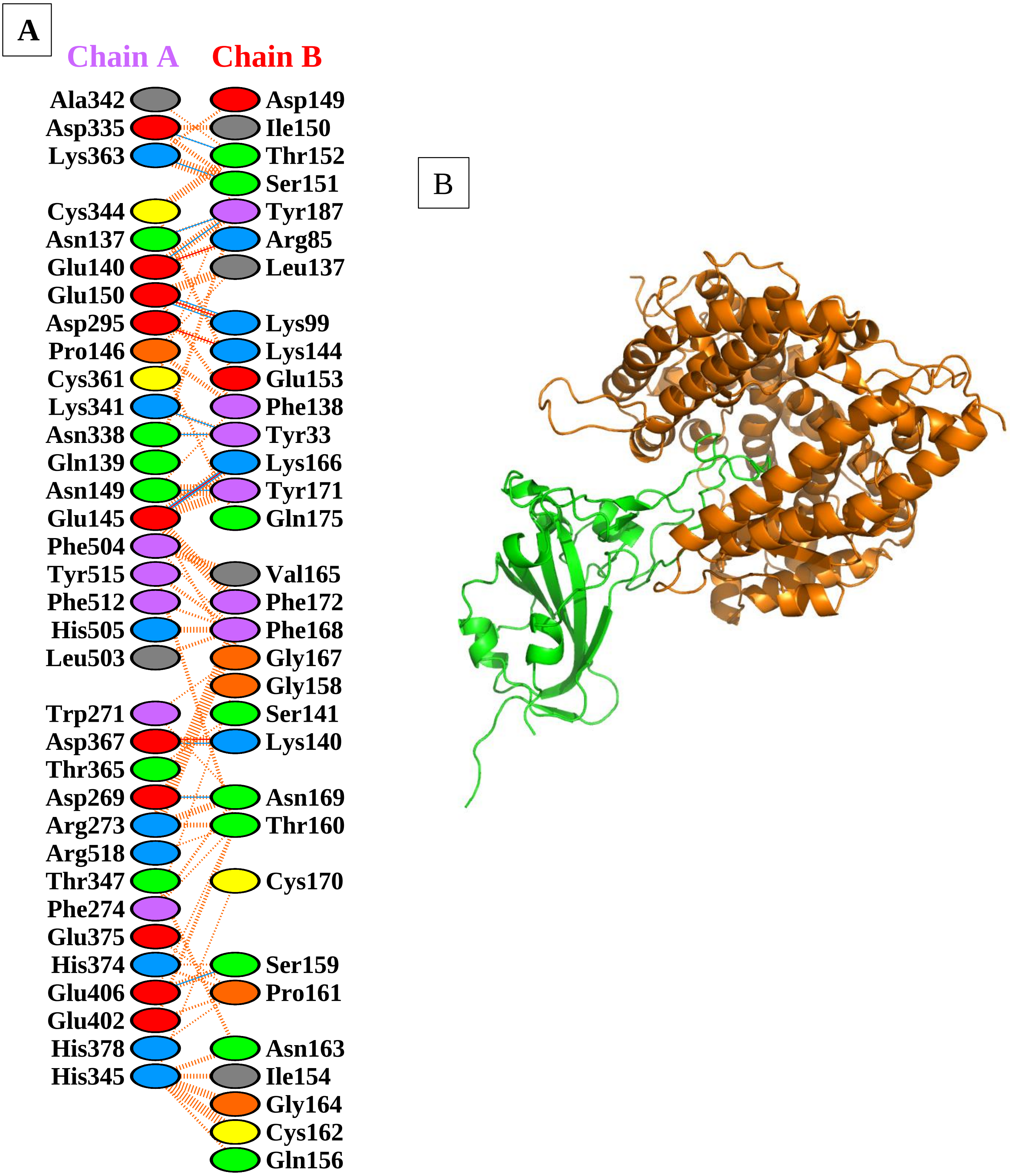

Supplementary Figure 2N: Protein–protein docking representation of hACE2 and RBD of B.1.526(Lota) (EPI_ISL_765494). (**A**) The interaction representation, which includes hydrogen (▬), salt bridges (▬), and non-bonded (❘❘❘❘❘❘) interactions. Chain A represents hACE2 and chain B represents RBD. Residue colors: positive (blue): H,K,R; negative (red): D,E; neutral (green): S,T,N,Q; aliphatic (grey): A,V,L,I,M; aromatic (purple): F,Y,W; Pro & Gly (orange): P,G. (**B**) The binding interface of hACE2:RBD complex

EXCLI Journal 2022;21:610-620 – ISSN 1611-2156
Received: January 26, 2022, accepted: February 25, 2022, published: March 10, 2022

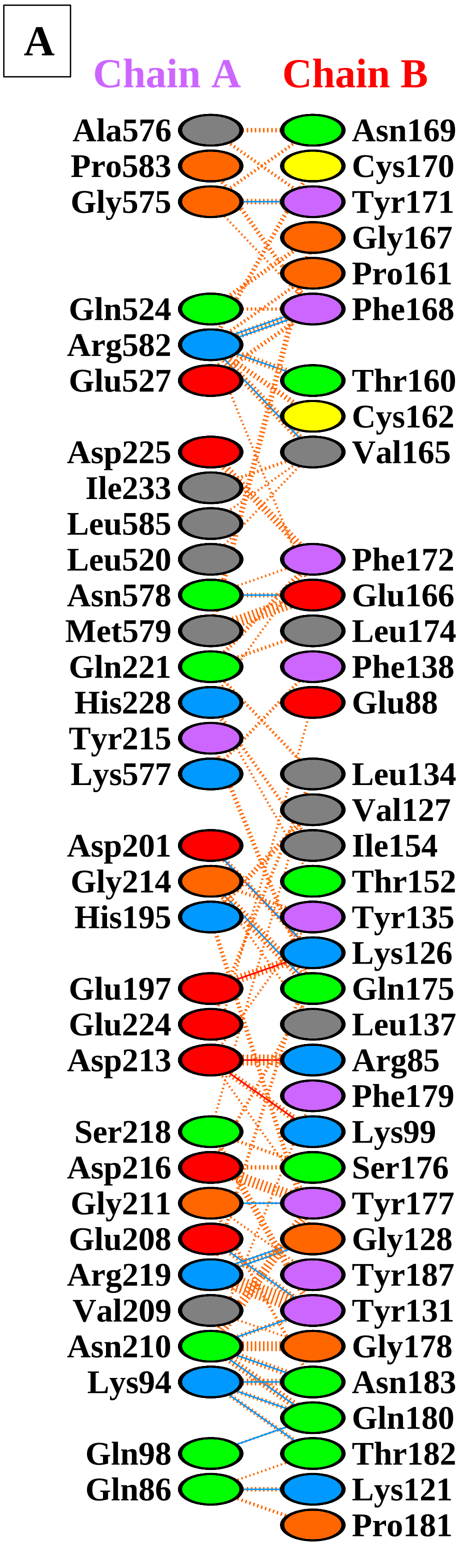

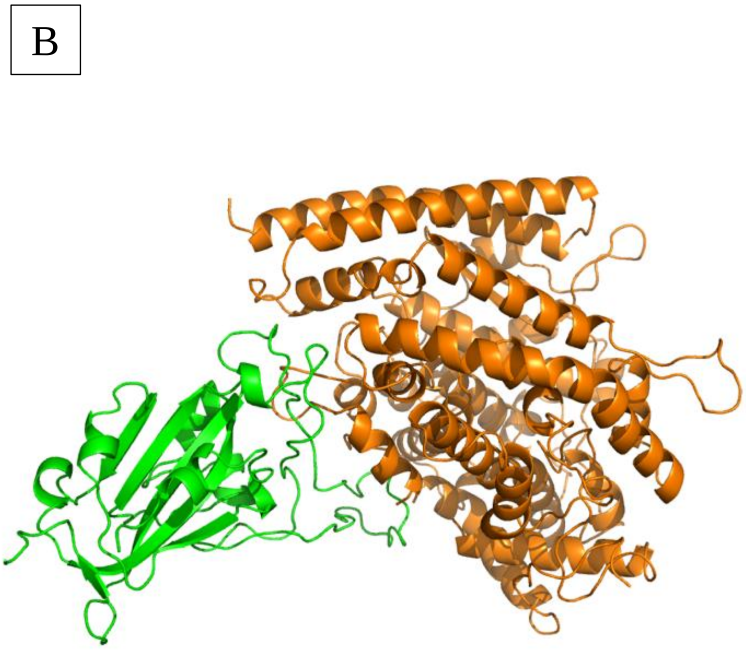

Supplementary Figure 2O: Protein–protein docking representation of hACE2 and RBD of B.1.466.2 (EPI_ISL_877419). (**A**) The interaction representation, which includes hydrogen (▬▬), salt bridges (▬▬), and non-bonded (IIIIII) interactions. Chain A represents hACE2 and chain B represents RBD. Residue colors: positive (blue): H,K,R; negative (red): D,E; neutral (green): S,T,N,Q; aliphatic (grey): A,V,L,I,M; aromatic (purple): F,Y,W; Pro & Gly (orange): P,G. (**B**) The binding interface of hACE2:RBD complex

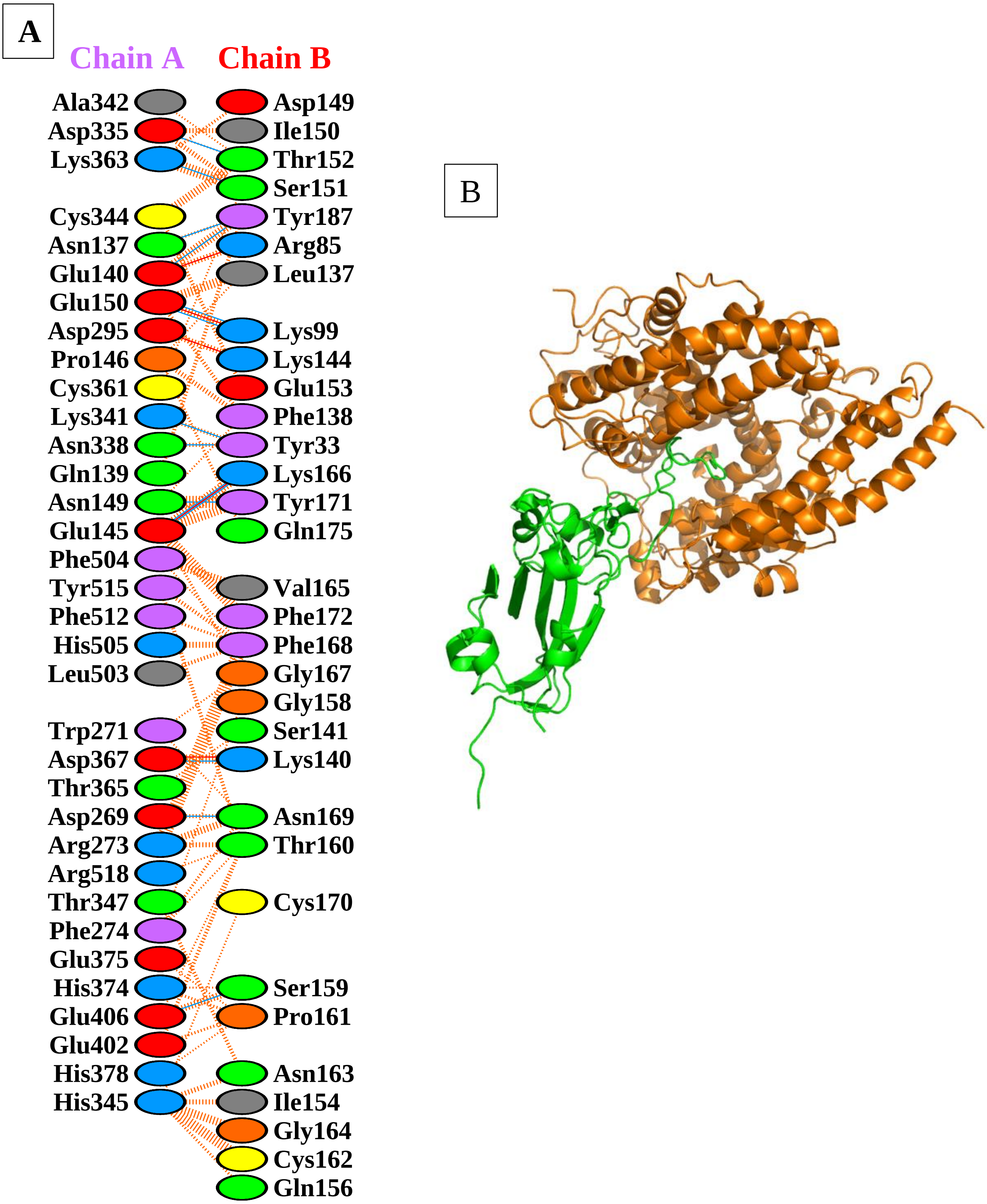

Supplementary Figure 2P: Protein–protein docking representation of hACE2 and RBD of B.1.1.318 (EPI_ISL_937654). (**A**) The interaction representation, which includes hydrogen (▬▬), salt bridges (▬▬), and non-bonded (IIIIIII) interactions. Chain A represents hACE2 and chain B represents RBD. Residue colors: positive (blue): H,K,R; negative (red): D,E; neutral (green): S,T,N,Q; aliphatic (grey): A,V,L,I,M; aromatic (purple): F,Y,W; Pro & Gly (orange): P,G. (**B**) The binding interface of hACE2:RBD complex

EXCLI Journal 2022;21:610-620 – ISSN 1611-2156
Received: January 26, 2022, accepted: February 25, 2022, published: March 10, 2022

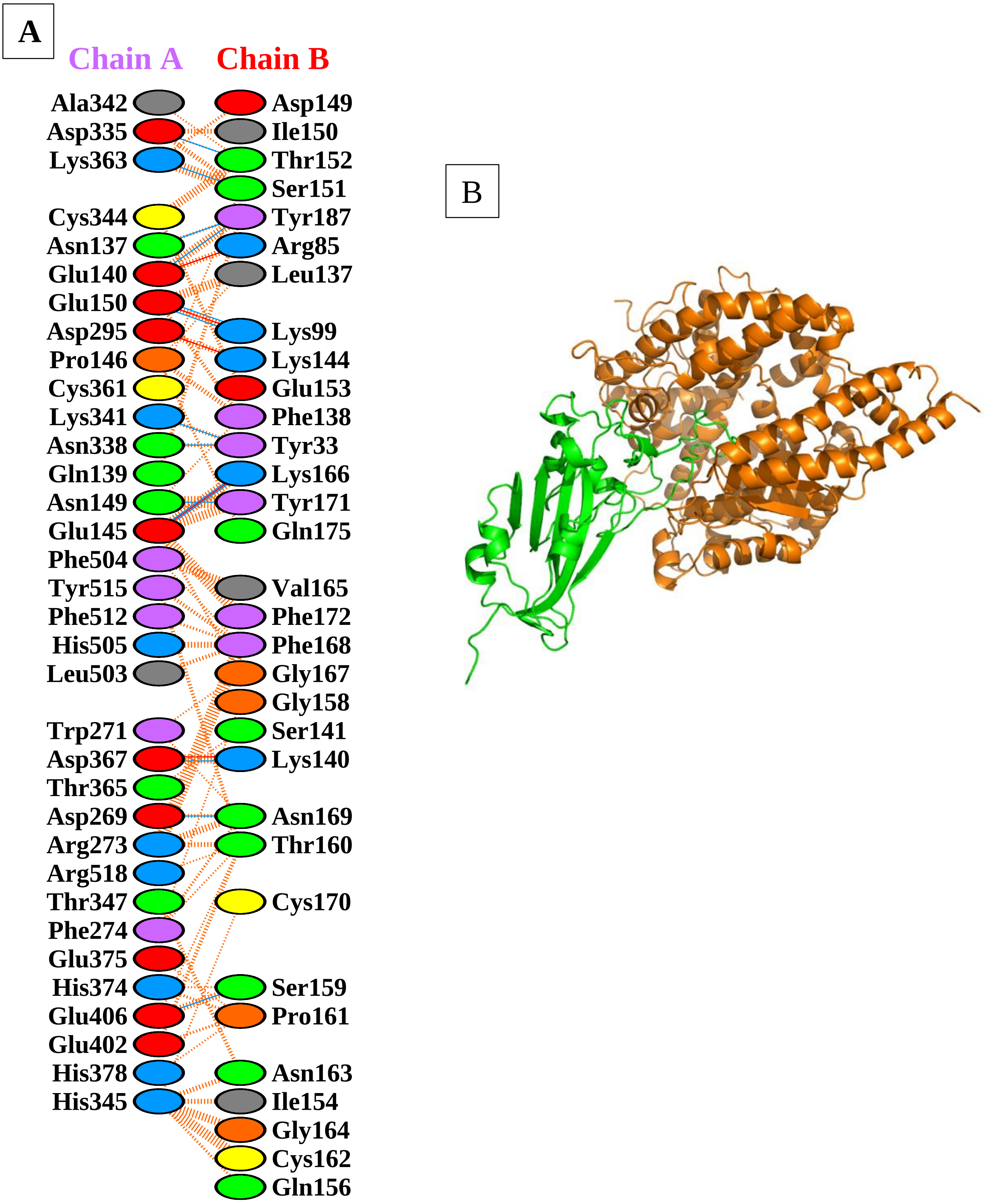

Supplementary Figure 2Q: Protein–protein docking representation of hACE2 and RBD of B.1.619 (EPI_ISL_1150929). (**A**) The interaction representation, which includes hydrogen (▬), salt bridges (▬), and non-bonded (IIIIII) interactions. Chain A represents hACE2 and chain B represents RBD . Residue colors: positive (blue): H,K,R; negative (red): D,E; neutral (green): S,T,N,Q; aliphatic (grey): A,V,L,I,M; aromatic (purple): F,Y,W; Pro & Gly (orange): P,G. (**B**) The binding interface of hACE2:RBD complex

EXCLI Journal 2022;21:610-620 – ISSN 1611-2156
Received: January 26, 2022, accepted: February 25, 2022, published: March 10, 2022

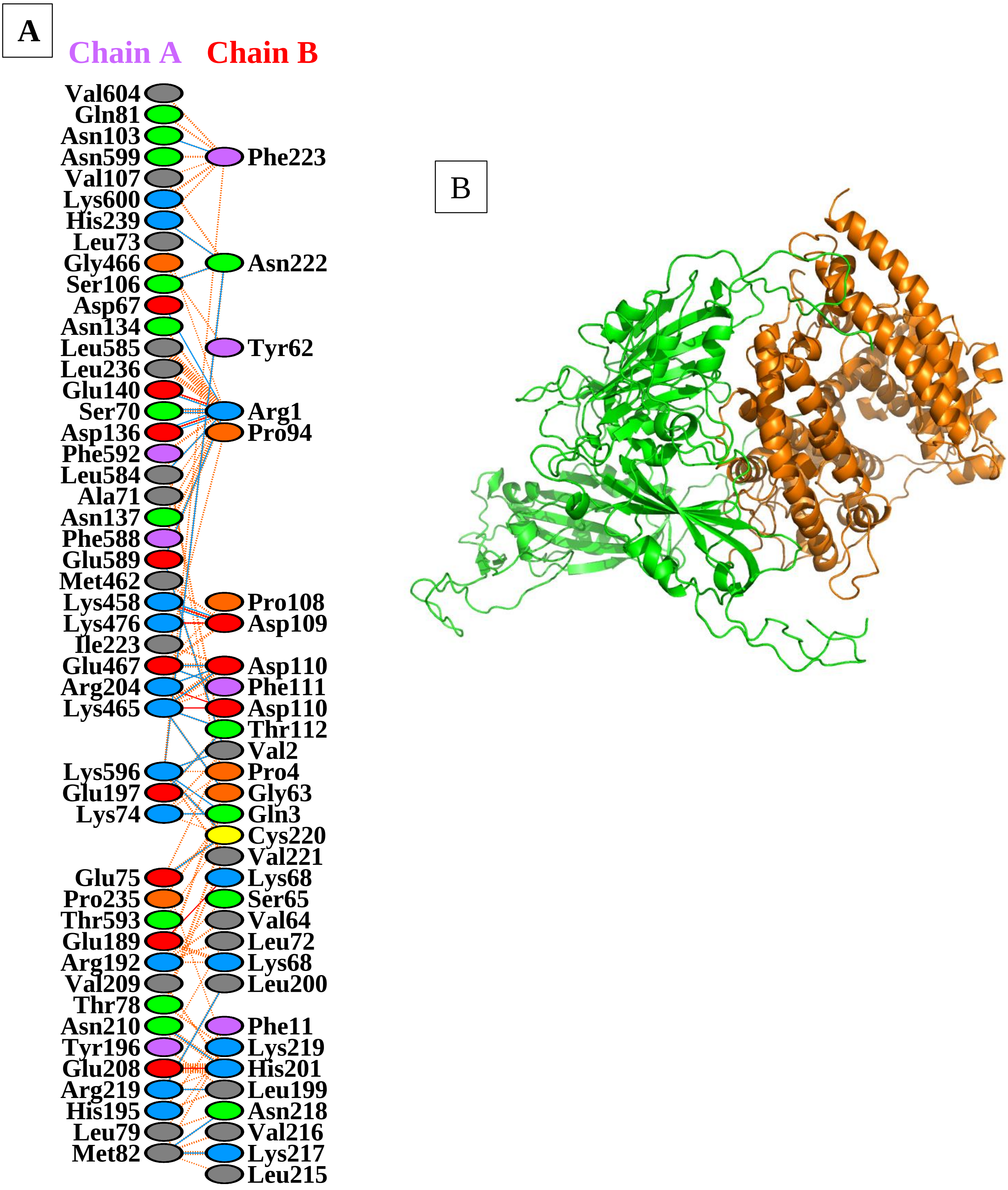

Supplementary Figure 2R: Protein–protein docking representation of hACE2 and RBD of C.36.3 (EPI_ISL_1237137). (**A**) The interaction representation, which includes hydrogen (▬▬), salt bridges (▬▬), and non-bonded (IIIIII) interactions. Chain A represents hACE2 and chain B represents RBD. Residue colors: positive (blue): H,K,R; negative (red): D,E; neutral (green): S,T,N,Q; aliphatic (grey): A,V,L,I,M; aromatic (purple): F,Y,W; Pro & Gly (orange): P,G. (**B**) The binding interface of hACE2:RBD complex

EXCLI Journal 2022;21:610-620 – ISSN 1611-2156
Received: January 26, 2022, accepted: February 25, 2022, published: March 10, 2022

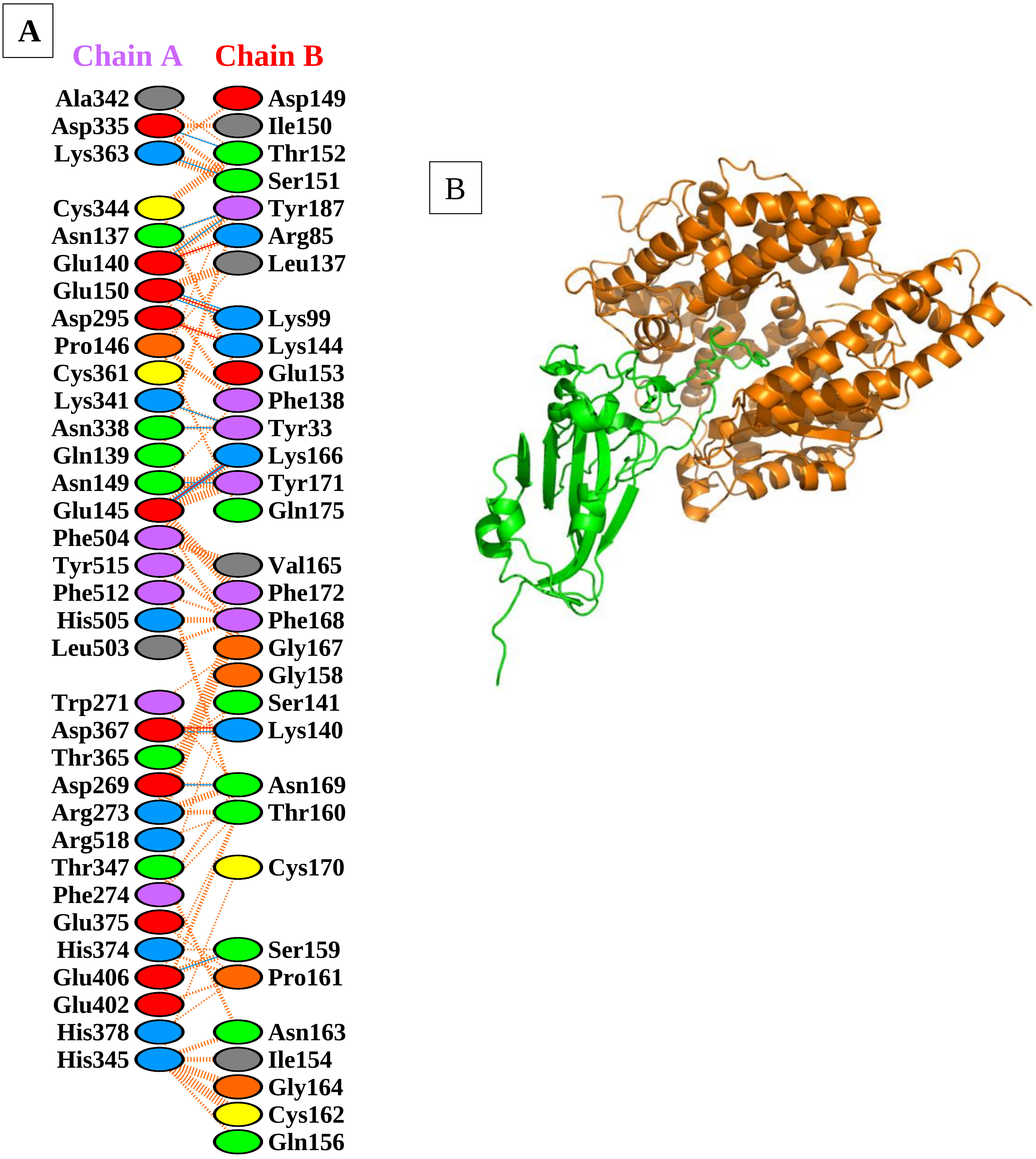

Supplementary Figure 2S: Protein–protein docking representation of hACE2 and RBD of AT.1 (EPI_ISL_1259283). (**A**) The interaction representation, which includes hydrogen (blue line), salt bridges (red line), and non-bonded (orange dashed) interactions. Chain A represents hACE2 and chain B represents RBD. Residue colors: positive (blue): H,K,R; negative (red): D,E; neutral (green): S,T,N,Q; aliphatic (grey): A,V,L,I,M; aromatic (purple): F,Y,W; Pro & Gly (orange): P,G. (**B**) The binding interface of hACE2:RBD complex

EXCLI Journal 2022;21:610-620 – ISSN 1611-2156
Received: January 26, 2022, accepted: February 25, 2022, published: March 10, 2022

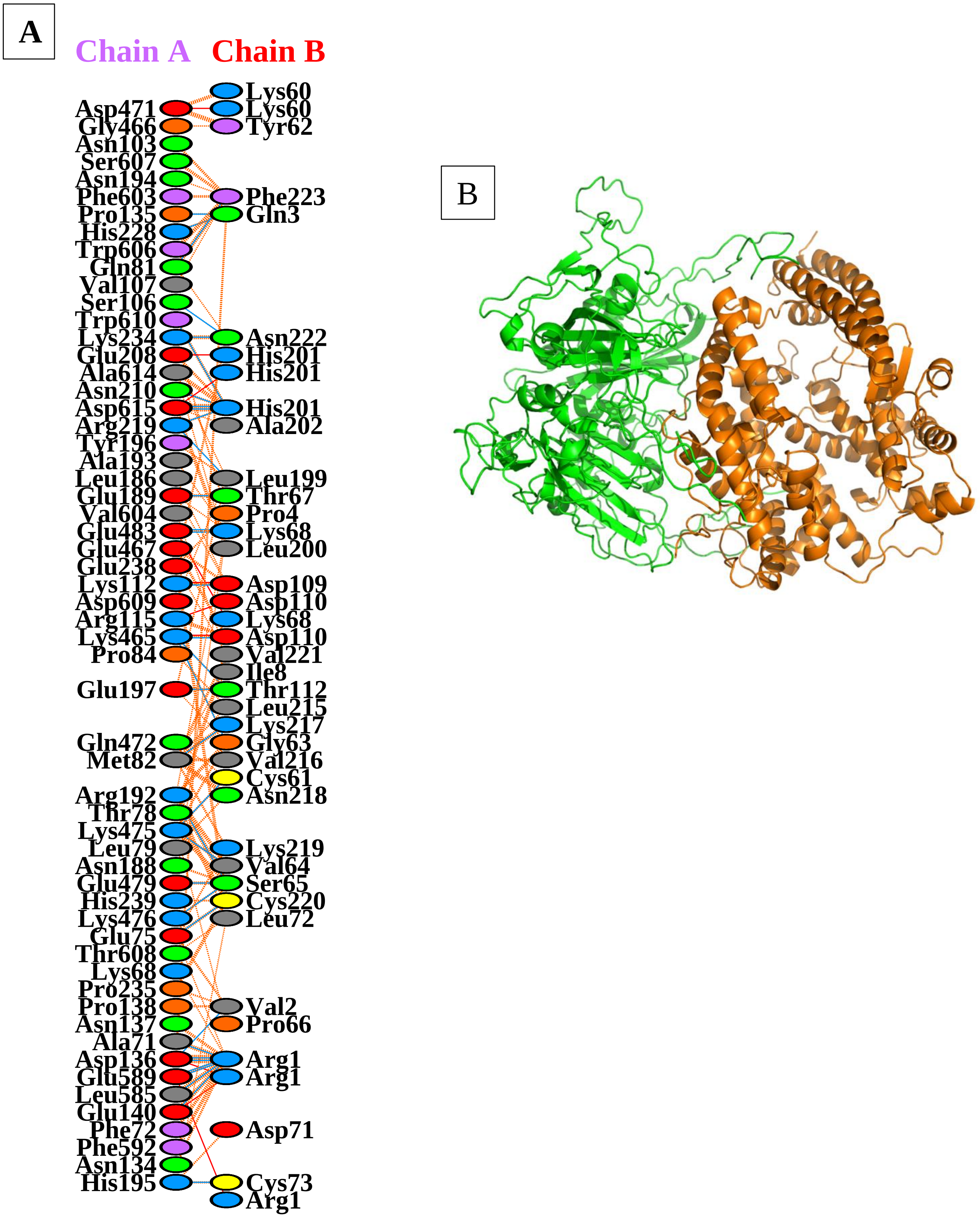

Supplementary Figure 2T: Protein–protein docking representation of hACE2 and RBD of B.1.617.1(Kappa) (EPI_ISL_1357699). (**A**) The interaction representation, which includes hydrogen (▬▬), salt bridges (▬▬), and non-bonded (|||||||) interactions. Chain A represents hACE2 and chain B represents RBD. Residue colors: positive (blue): H,K,R; negative (red): D,E; neutral (green): S,T,N,Q; aliphatic (grey): A,V,L,I,M; aromatic (purple): F,Y,W; Pro & Gly (orange): P,G. (**B**) The binding interface of hACE2:RBD complex

Received: January 26, 2022, accepted: February 25, 2022, published: March 10, 2022

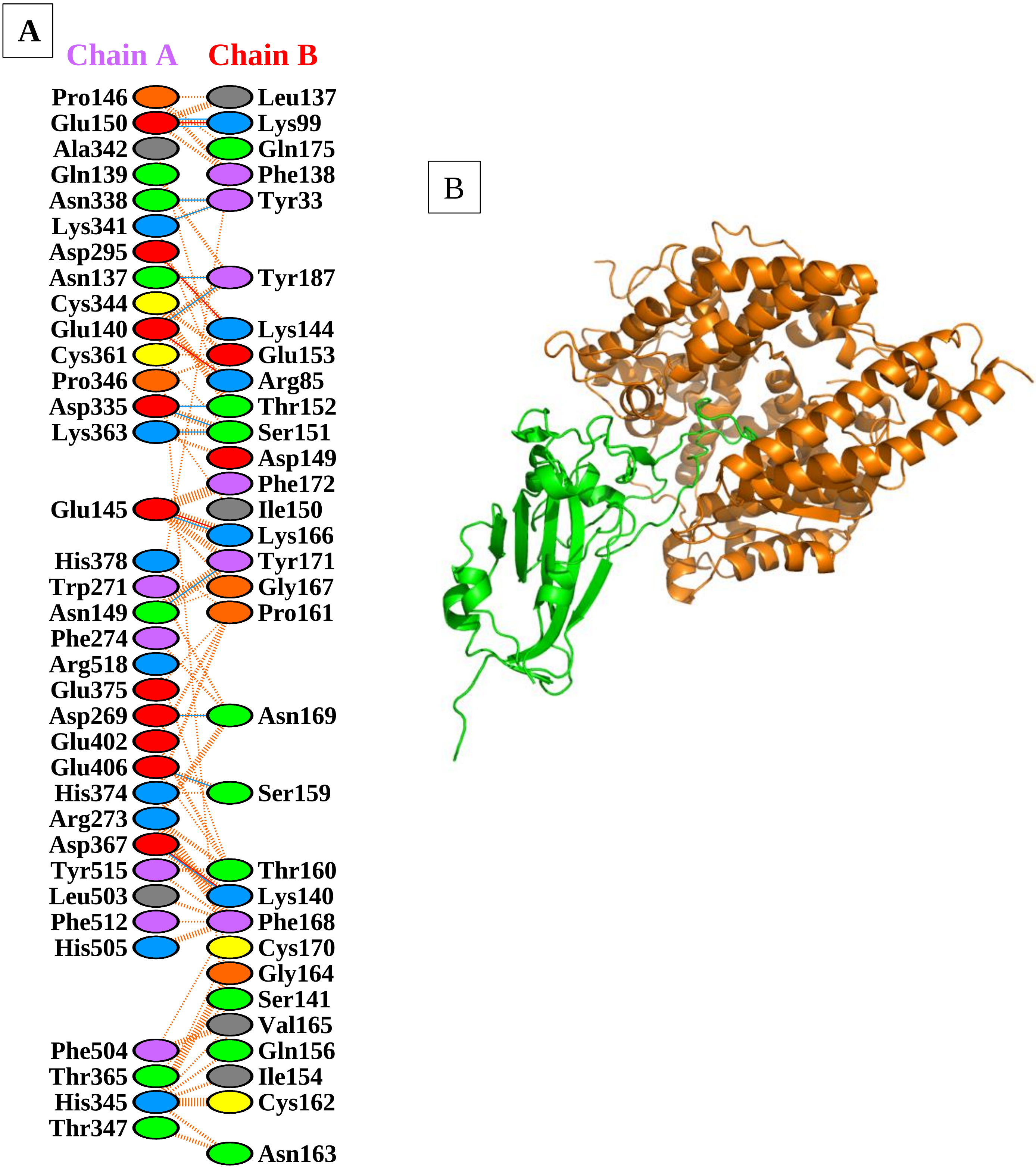

Supplementary Figure 2U: Protein–protein docking representation of hACE2 and RBD of B.1.1.523 (EPI_ISL_1448584) . (**A**) The interaction representation, which includes hydrogen (▬▬), salt bridges (▬▬), and non-bonded (IIIIIII) interactions. Chain A represents hACE2 and chain B represents RBD. Residue colors: positive (blue): H,K,R; negative (red): D,E; neutral (green): S,T,N,Q; aliphatic (grey): A,V,L,I,M; aromatic (purple): F,Y,W; Pro & Gly (orange): P,G. (**B**) The binding interface of hACE2:RBD complex

EXCLI Journal 2022;21:610-620 – ISSN 1611-2156
Received: January 26, 2022, accepted: February 25, 2022, published: March 10, 2022

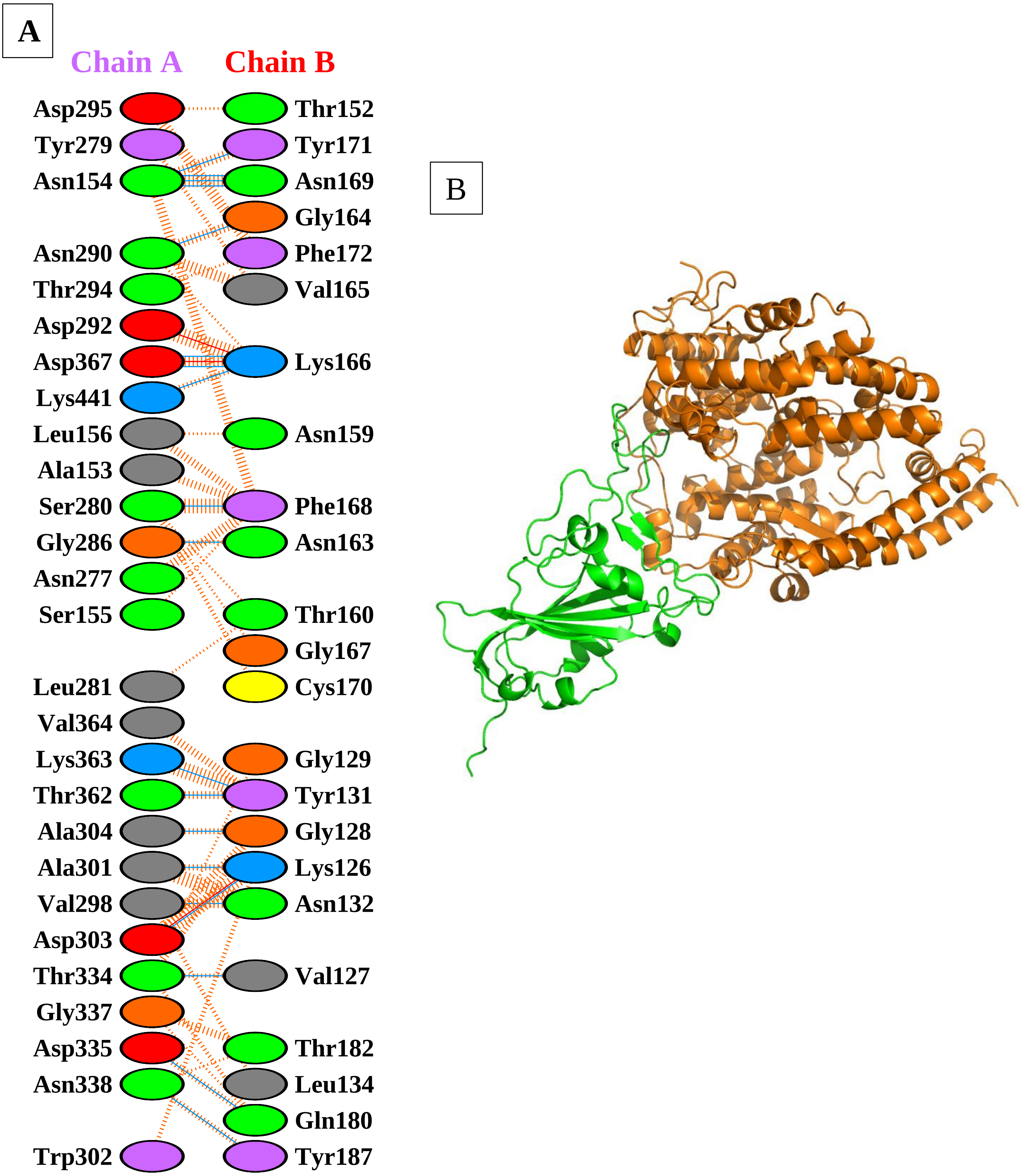

Supplementary Figure 2V: Protein–protein docking representation of hACE2 and RBD of B.1.620 (EPI_ISL_1579527). (**A**) The interaction representation, which includes hydrogen (▬), salt bridges (▬), and non-bonded (IIIIII) interactions. Chain A represents hACE2 and chain B represents RBD. Residue colors: positive (blue): H,K,R; negative (red): D,E; neutral (green): S,T,N,Q; aliphatic (grey): A,V,L,I,M; aromatic (purple): F,Y,W; Pro & Gly (orange): P,G. (**B**) The binding interface of hACE2:RBD complex

EXCLI Journal 2022;21:610-620 – ISSN 1611-2156
Received: January 26, 2022, accepted: February 25, 2022, published: March 10, 2022

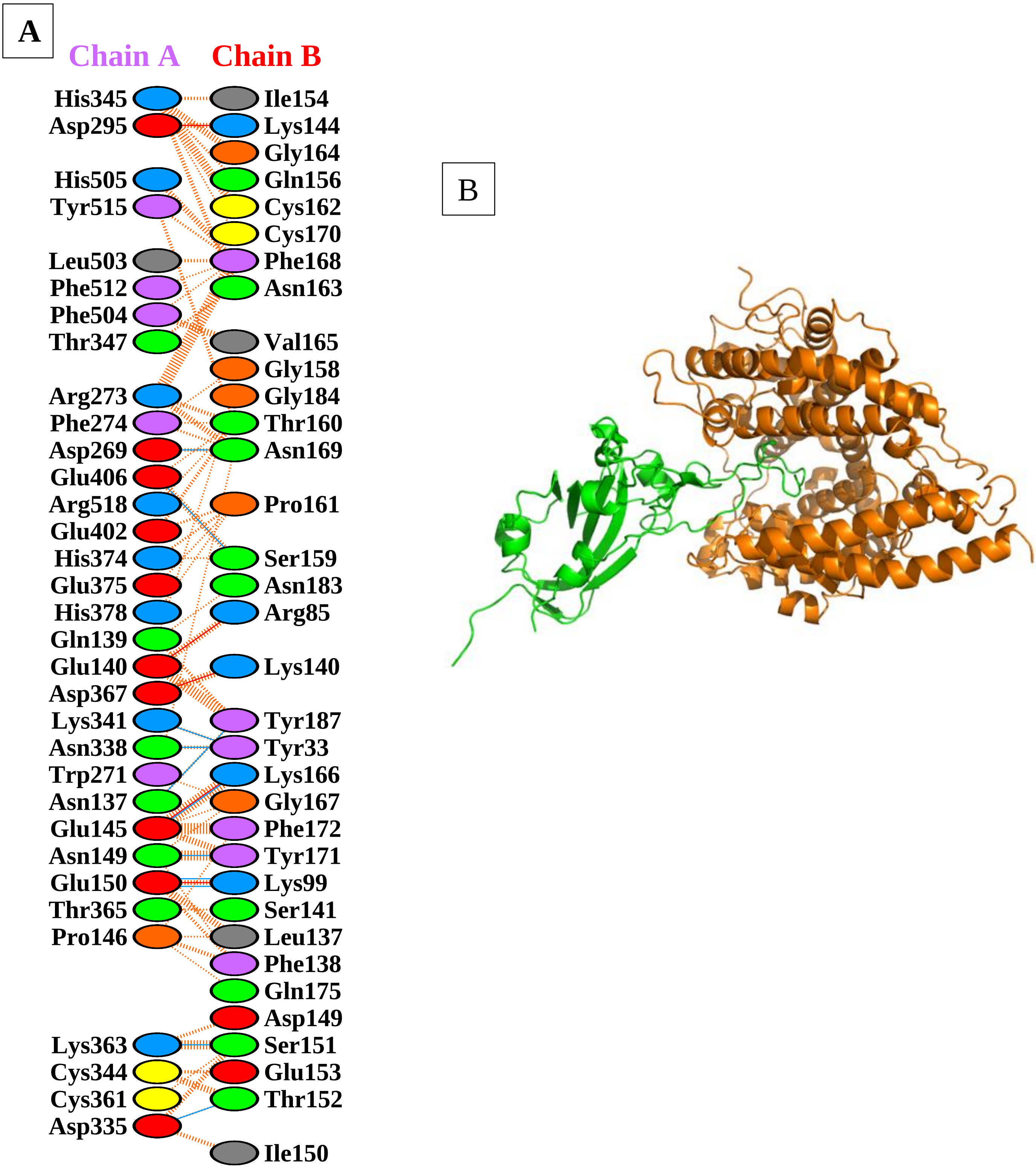

Supplementary Figure 2W: Protein–protein docking representation of hACE2 and RBD of AV.1 (EPI_ISL_1595332). (**A**) The interaction representation, which includes hydrogen (▬), salt bridges (▬), and non-bonded (||||||) interactions. Chain A represents hACE2 and chain B represents RBD. Residue colors: positive (blue): H,K,R; negative (red): D,E; neutral (green): S,T,N,Q; aliphatic (grey): A,V,L,I,M; aromatic (purple): F,Y,W; Pro & Gly (orange): P,G. (**B**) The binding interface of hACE2:RBD complex

EXCLI Journal 2022;21:610-620 – ISSN 1611-2156
Received: January 26, 2022, accepted: February 25, 2022, published: March 10, 2022

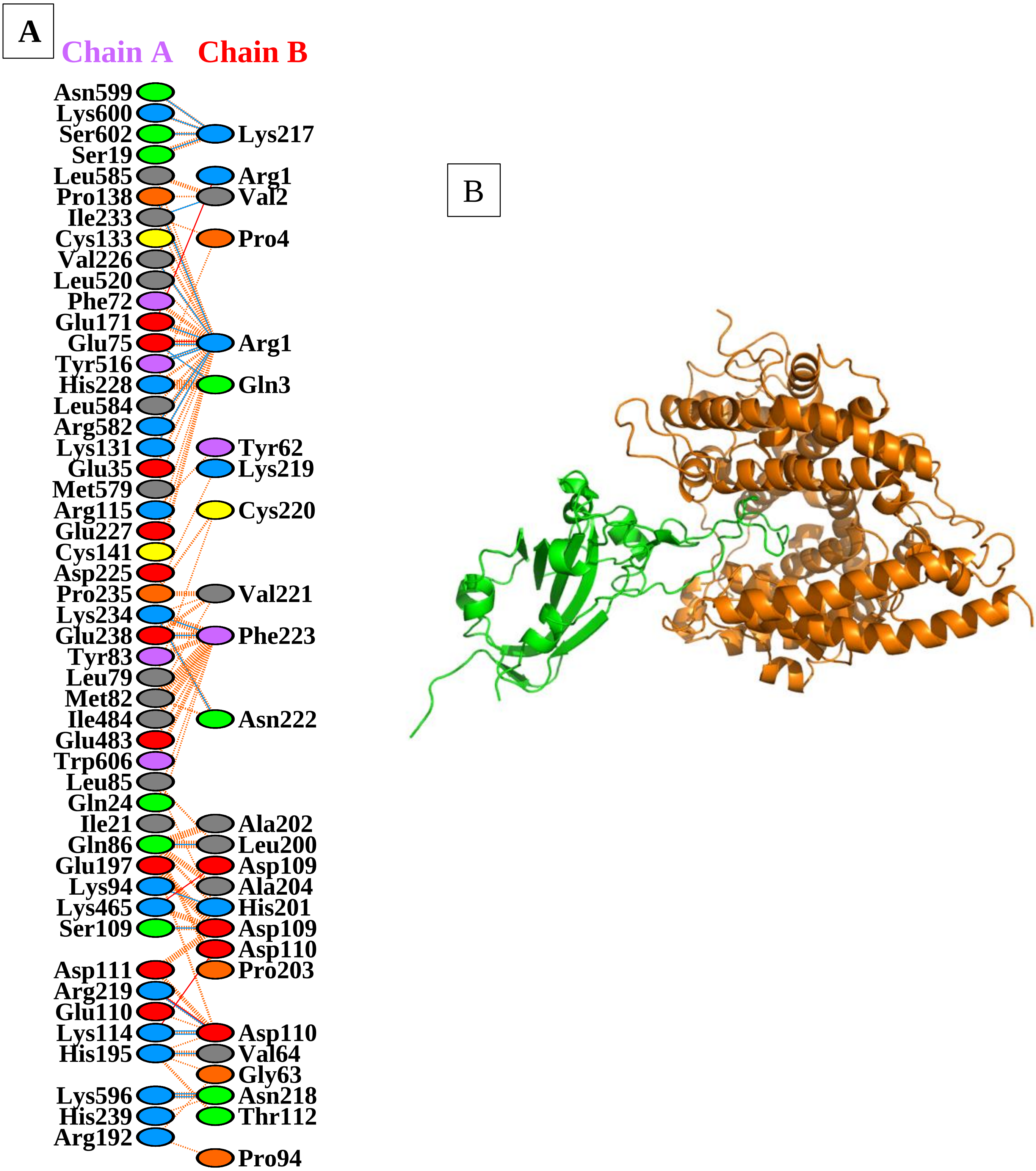

Supplementary Figure 2X: Protein–protein docking representation of hACE2 and RBD of B.1.630 (EPI_ISL_3045385). (**A**) The interaction representation, which includes hydrogen (blue line), salt bridges (red line), and non-bonded (orange dashes) interactions. Chain A represents hACE2 and chain B represents RBD. Residue colors: positive (blue): H,K,R; negative (red): D,E; neutral (green): S,T,N,Q; aliphatic (grey): A,V,L,I,M; aromatic (purple): F,Y,W; Pro & Gly (orange): P,G. (**B**) The binding interface of hACE2:RBD complex

EXCLI Journal 2022;21:610-620 – ISSN 1611-2156
Received: January 26, 2022, accepted: February 25, 2022, published: March 10, 2022

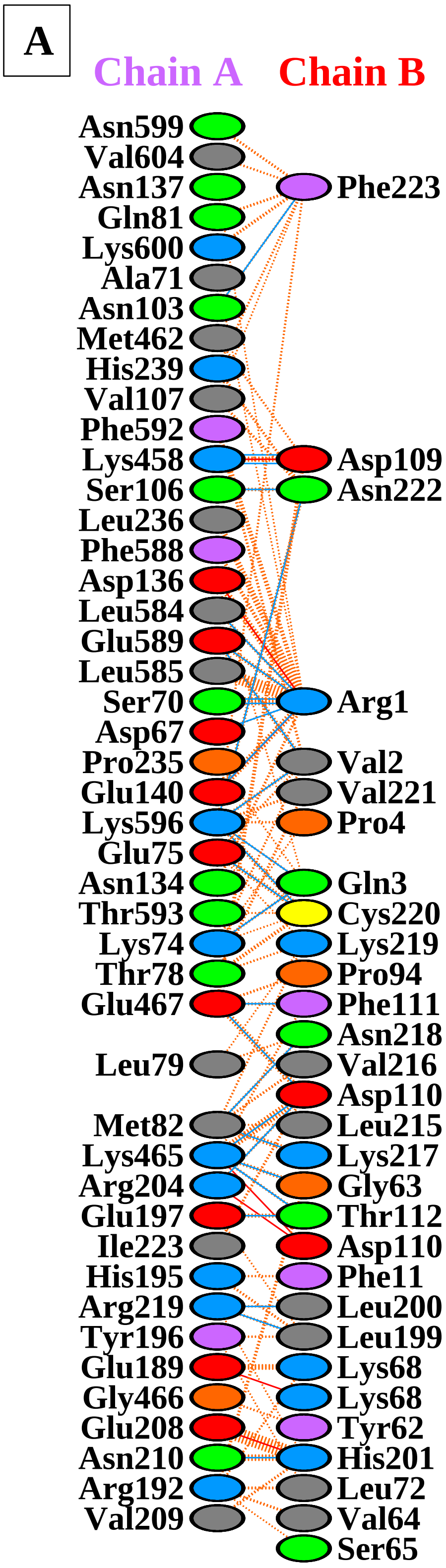

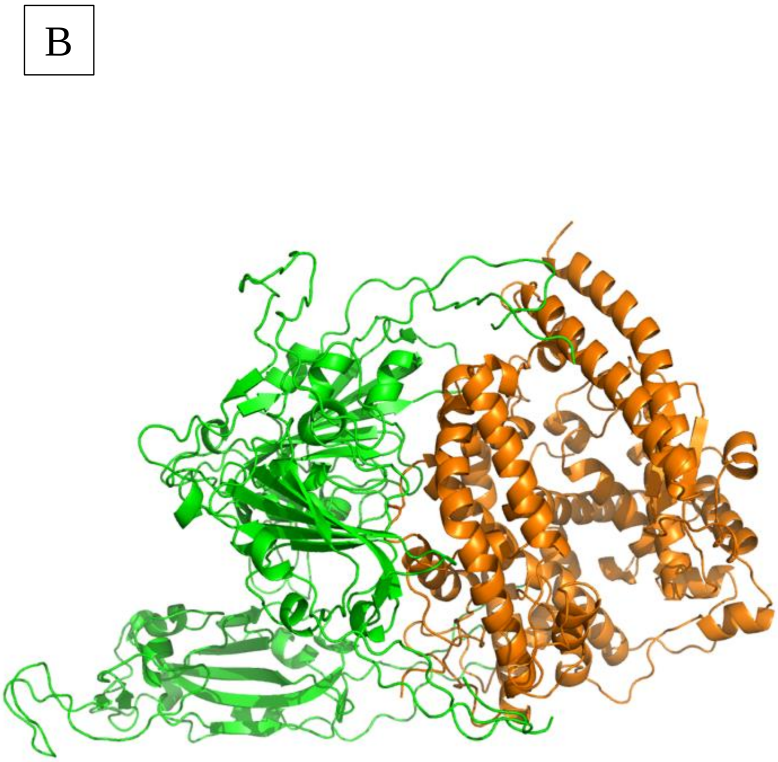

Supplementary Figure 2Y: Protein–protein docking representation of hACE2 and RBD of C.1.2 (EPI_ISL_3447714) . (**A**) The interaction representation, which includes hydrogen (▬), salt bridges (▬), and non-bonded (IIIIIII) interactions. Chain A represents hACE2 and chain B represents RBD. Residue colors: positive (blue): H,K,R; negative (red): D,E; neutral (green): S,T,N,Q; aliphatic (grey): A,V,L,I,M; aromatic (purple): F,Y,W; Pro & Gly (orange): P,G. (**B**) The binding interface of hACE2:RBD complex

EXCLI Journal 2022;21:610-620 – ISSN 1611-2156
Received: January 26, 2022, accepted: February 25, 2022, published: March 10, 2022

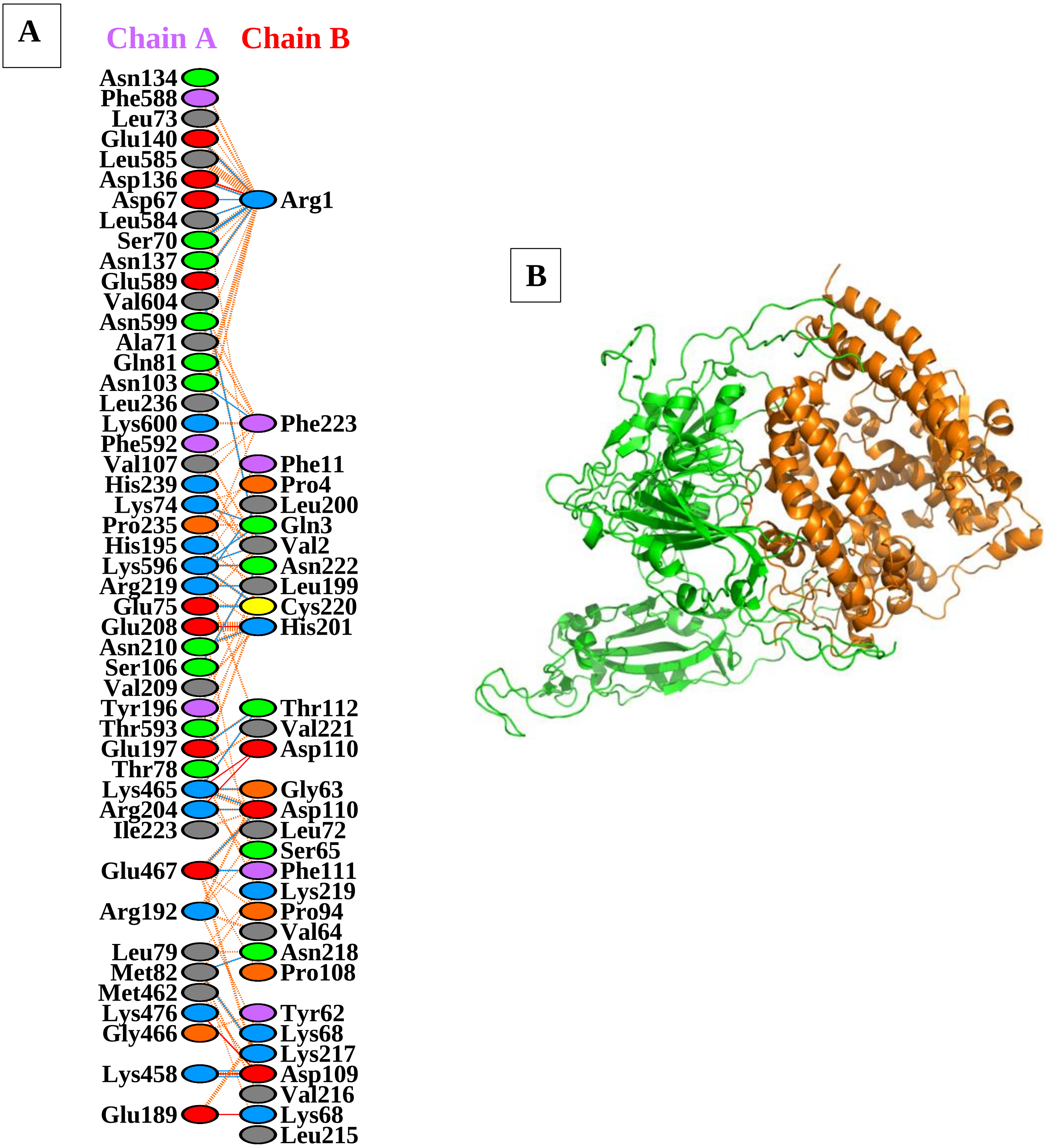

Supplementary Figure 2Z: Protein–protein docking representation of hACE2 and RBD of B.1.640 (EPI_ISL_5592661). (**A**) The interaction representation, which includes hydrogen (▬), salt bridges (▬), and non-bonded (IIIIII) interactions. Chain A represents hACE2 and chain B represents RBD. Residue colors: positive (blue): H,K,R; negative (red): D,E; neutral (green): S,T,N,Q; aliphatic (grey): A,V,L,I,M; aromatic (purple): F,Y,W; Pro & Gly (orange): P,G. (**B**) The binding interface of hACE2:RBD complex
